# A Novel Immune-Related Prognostic Signature Predicting Survival in Patients with Pancreatic Adenocarcinoma

**DOI:** 10.1155/2022/8909631

**Published:** 2022-03-18

**Authors:** Xiao Zhang, Xiaomian Li, Jun Xie, Qian Zhu, Yufeng Yuan

**Affiliations:** ^1^Department of Hepatobiliary and Pancreatic Surgery, Zhongnan Hospital of Wuhan University, Wuhan 430071, China; ^2^Department of Gastrointestinal Surgery, Zhongnan Hospital of Wuhan University, School of Medicine, Xiamen 361004, China

## Abstract

Pancreatic adenocarcinoma (PAAD) carries the lowest survival rate of all major organ cancers, which is of dismal prognosis and high mortality rate. Thus, the present study attempted to identify a few novel prognostic biomarkers and establish an immune-related prognostic signature which could predict the prognosis of PAAD. Four prognostic immune-related genes (IRGs) including S100A6, S100A10, S100A16, and SDC1 were screened by differentially expressed gene (DEG) identification and weighted gene coexpression network analysis (WGCNA). Subsequent analysis proved the high expression of these IRGs in PAAD tissues, suggested by TCGA-PAAD data, merged microarray-acquired dataset (MMD), GEPIA, and Oncomine webtool. By using MMD and TCGA-PAAD data, S100A6 (MMD: AUC = 0.897; TCGA: AUC = 0.843), S100A10 (MMD: AUC = 0.880; TCGA: AUC = 0.780), S100A16 (MMD: AUC = 0.878; TCGA: AUC = 0.838), and SDC1 (MMD: AUC = 0.885; TCGA: AUC = 0.812) exhibited excellent diagnostic efficiency for PAAD. By conducting connectivity map (CMap) analysis, we concluded that three molecule drugs (sulpiride, famotidine, and nalidixic acid) might have worked in the treatment of PAAD. Then, an immune-related prognostic index was constructed, which was validated as an independent prognostic factor for PAAD patients (*P*=0.004). We further constructed a nomogram by using this immune-related signature and age, the prognostic value of which was validated by using concordance index (C-index = 0.780) and area under curve (AUC = 0.909). Moreover, the immune-related prognostic signature was associated with response to anti-PD-1/L1 immunotherapy. To sum up, four IRGs were screened out and verified to be novel immune-related prognostic biomarkers in PAAD. Besides, sulpiride, famotidine, and nalidixic acid might be potential choices in the treatment of PAAD. An immune-related signature was established to show great potential for prognosis prediction for PAAD, independently, which might guide more effective immunotherapy strategies. A nomogram is further established by using this immune-related prognostic index, which might contribute to more effective prognosis prediction in PAAD patients.

## 1. Introduction

Pancreatic adenocarcinoma (PAAD) mainly refers to a malignant tumor derived from pancreatic duct epithelial cells and follicular cells, which is a common malignant tumor of digestive system [1]. According to the data of the National Comprehensive Cancer Network Center in 2020, about 540,000 people were diagnosed with pancreatic cancer in the United States in 2019, and the number of deaths due to pancreatic cancer was about 430,000 [2]. The incidence rate of pancreatic cancer is the top ten among the leading cancers in the United States, ranking third in the cancer-related death causes, as the American Cancer Society reported [2]. Pancreatic ductal adenocarcinoma (PDAC) is the most common type of pancreatic adenocarcinoma, which comes from pancreatic duct epithelial cells, accounting for 80%∼90% of all pancreatic cancer patients [3]. Some studies indicate that the mortality of PDAC is tightly related to incidence [4]. And what is worse is the mortality of PDAC ranks the fourth commonest reason of cancer-related death worldwide [5]. Nowadays, the fundamental treatment principle is still based on surgery, combining with comprehensive treat methods such as radiotherapy and chemotherapy. Despite great efforts, PDAC is still of poor survival, 5-year survival of which is less than seven percent [6, 7]. Because of the poor prognosis of PAAD, there might be a great need of novel prognostic biomarker exploring.

As a kind of cancer therapy, immunotherapy could fight cancer based on immune system [8]. At present, there is a growing realization that immunotherapy might show potential to treat tumors effectively and safely [9–11]. Bioinformatics have made significant progress recent years, which promoted the increasing use of mining of public databases in cancer biomarkers identification. With theory researches going deep, immune-related genes (IRGs) have shown potential among development and immunotherapy of several cancers [12–14]. Chen et al. have developed a prognostic signature for head and neck squamous cell carcinoma via IRGs, which could effectively distinguish the prognosis, the molecular and immune characteristics, and immune benefit from immune checkpoint inhibitor therapy [15]. These researches determined that immune-related predictive biomarkers could enhance immunotherapy efficacy [16, 17]. Screening of prognostic biomarkers via IRGs has without question been immune therapy of cancer research hotspot. Lack of prognostic biomarkers related to the tumor immune microenvironment for PAAD patients stimulates us to explore some immune-related biomarkers in PAAD, which might guide appropriate therapy tips to improve the therapeutic efficacy in PAAD.

So far as we know, this research might be the first one to screen immune-related prognostic biomarkers for PAADs straight by bioinformatics. We firstly collected nine datasets from Gene Expression Omnibus (GEO) and The Cancer Genome Atlas (TCGA) database. Then conducting WGCNA via immune-related genes in PAAD. Hub genes reaching the standards have been screened out from the key module. Furthermore, 22 overlapped DEGs were selected, four of which were common with hub genes. Thus, the four IRGs were considered to be initial prognostic factors and further validated using other methods. After that, a risk signature via the four IRGs was constructed, which might be a novel prognosis prediction tool for PAAD and guide more effective immunotherapy strategies. In addition, we developed a nomogram comprehensively considering the risk signature, clinical characters. The nomogram established for forecasting the survival rate of PAAD could give some beneficial guidance for clinical application.

## 2. Materials and Methods

### 2.1. Dataset and Immune-Related Gene Collection

**Figure 1:**
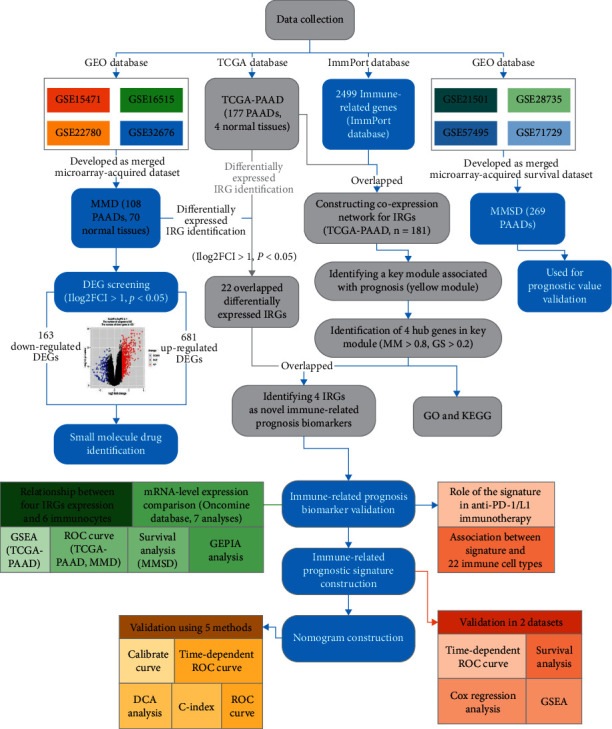
The flow diagram of this study. Data preparing, analysis and validation was shown in the flow diagram.

The research steps in this study are described in Figure 1, showing the identification and validation of immune-related prognostic biomarkers in PAAD. PAAD microarray data were firstly downloaded from TCGA database (https://genomecancer.ucsc.edu/). The microarray matrix were displayed as count number. We removed tumor samples without complete clinical information from subsequent analysis. In other words, all the samples left contained completed survival information and essential clinical factors (age, gender, tumor grade, and tumor stage). TCGA-PAAD data standardization including normalization and log2 transformation were in process having the aid of R package “DEseq.2.” 4 normal samples and 177 PAADs were used for subsequent process.

Then four independent GEO datasets (GSE15471 [18], GSE16515 [19], GSE22780, and GSE32676 [20]) were collected through GEO database (http://www.ncbi.nlm.nih.gov/geo/). Because these datasets used the same annotation platform (GPL570), we merged them to develop a merged microarray-acquired dataset (MMD). We firstly downloaded the raw data for the four datasets. Secondly, we normalized the raw data using RMA-normalization method, based on package “affy” [21] in the R software. Then, we preprocessed, merged, and ComBat-adjusted the four datasets based on R package insilico-Merging. A PAAD-specific, MMD was generated though the above steps. Finally, we used the GPL570 annotation files for annotation of probes. After finishing these procedures, 70 normal tissues and 108 PAAD tissues were collected and further used in this research.

Besides, with the aim of validation of immune-related prognostic biomarkers, three independent GEO datasets (GSE21501 [22], GSE28735 [23], and GSE71729 [24]) with complete survival information were retrieved from GEO database. Raw expression data of GSE28735 was firstly retrieved from GEO database and further normalized and transformed via “affy” package, as previous done. For GSE21501, we used locally weighted linear regression (Lowess) for normalization. For GSE71729, nonnegative normalization was conducted. Then we merged the three datasets to develop a merged microarray-acquired survival dataset (MMSD) as we previous did. Totally 269 PAADs were included in MMSD. The detail information of the eight datasets were showed in Table 1.

IRGs were retrieved from ImmPort database (https://www.immport.org). We obtained 2,499 IRGs from this database. The 1,650 overlapped genes with the TCGA-PAAD gene list were selected for next-step study.

### 2.2. WGCNA to Screen Key Module

Firstly, the expression matrix of the 1,650 IRGs was checked via two approaches (goodSamplesGenes and sample-network-method). Outliers were further identified with this cut-off criterion of *Z*.Ku < −2.5 (*Z*.ku *=* (ku-mean(*k*))/(sqrt(var(*k*)))). We removed the substandard samples from further analysis. Then a coexpression network was established via “WGCNA” [25]. Branch cutting methods was conducted to classify IRGs into gene modules [26]. Some important parameters in branch cutting methods were set including minClusterSize = 30, and deepSplit = 2. After splitting IRGs into gene modules, a cut line (correlation ≥ 0.75) for combining high related modules was selected, which was realized by measuring the dissimilarity of module eigengenes (MEs). With the aim of screening hub modules associated with disease status (PAAD or normal, the trait interested us most), we firstly calculated the Gene Significance (GS). Furthermore, module significance (MS) was calculated based on GS. MS was measured as the average GS of all the genes in a module. After finishing the above-mentioned processes, the most correlated module was selected, which might be a key module. Furthermore, we regarded genes with the cut-off criterion (|cor.geneModuleMembership| >0.8 and |cor.geneTraitSignificance| >0.2) as key genes in WGCNA, which were included for next-step analysis.

### 2.3. Differentially Expressed Immune-Related Gene Identification

Based on TCGA-PAAD data (1,650 IRGs), we firstly screened out differentially expressed genes (DEGs) between normal tissues and PAADs by R package “edgeR” [27]. Similarly, based on “limma” [28], DEGs were identified by using the expression matrix of 1,242 IRGs in MMD. Genes with adjusted *P* value <0.05 and |log2FC| ≥ 1.0 were regarded significantly different expressed. Finally, DEGs overlapping between DEGs from TCGA-PAAD and DEGs from MMD were chosen for further analysis.

### 2.4. Hub Gene Identification

Combining WGCNA and DEG, hub gene were identified. Genes which could be found both in key biomarkers in WGCNA and DEGs were considered to be hub genes for further analysis. Gene Ontology (GO) enrichment analysis and Kyoto Encyclopedia of Genes and Genomes (KEGG) pathway analysis were performed via R package “clusterProfiler” [29] for functional annotation of hub genes. For the GO part, we just obtained the biological process (BP). We selected *P* < 0.05 as the standards to define significant BPs and KEGG pathway terms.

### 2.5. Small Molecule Drug Exploring

We also attempted to explore some small molecule drugs which might be novel choices for PAAD therapy. Based on R package “edgeR”, DEGs were firstly obtained based on the expression data profile of TCGA-PAAD data. DEGs were screened out using the same cut-off criterion we set before. Based on these DEGs, we conducted connectivity map (Cmap) (https://portals.broadinstitute.org/cmap/) [30] analysis to explore small molecule drugs. A small molecule drug with *P* value <0.05 and |mean ≥ 0.50 was concluded to show strong potential in the treatment of PAAD.

### 2.6. Internal Validation of Hub Genes

To explore and verify the prognostic value of key biomarkers, Gene Expression Profiling Interactive Analysis (GEPIA) webtool [31] was used to perform overall survival (OS) and disease-free survival (DFS) analyses (http://gepia.cancer-pku.cn/). Furthermore, the expression difference between normal samples and PAADs was also obtained via this webtool (to validate the result of DEG screening).

### 2.7. Verification of Expression of Hub Genes

Besides exploration via GEPIA, the mRNA-level expression difference of hub IRGs was verified with the help of Oncomine database (https://www.oncomine.org/) [32]. Seven analysis were retrieved from Oncomine database and used for expression exploration. The mRNA-level expression of hub genes between normal tissues and PAAD tissues were also obtained via The Human Protein Atlas (HPA) database (https://www.proteinatlas.org/).

### 2.8. Prognostic Role of Hub Genes Exploration

To validate the prognostic value of hub genes, survival analysis and receiver operating characteristic (ROC) analysis were conducted. Based on GSE21501 (*n* = 102), GSE28735 (*n* = 42), GSE57495 (*n* = 63), and GSE71729 (*n* = 125), PAADs were divided into high- and low-expression groups respectively according to the best calculated cut-off by R package “maxstat” from the beginning. OS analysis was next performed via R package “survival” [33]. Secondly, by using TCGA-PAAD and MMD large datasets, we plotted ROC curves to see if hub genes could distinguish PAADs and normal samples. By R package “pROC” [34], we did this analysis. Moreover, the area under curve (AUC) was also calculated. We considered a hub gene to have strong prognostic value when AUC > 0.75 in both the datasets.

### 2.9. Association between Hub Gene Expression and Immunocytes Exploring

There is a realization that immunocytes might act as independent predictors of survival in cancers. Therefore, the association between IRGs and immunocytes were explored via TIMER (https://cistrome.shinyapps.io/timer/) [35]. A hub gene with |correlation coefficient (cor) | ≥0.2 and *P* value <0.05 was thought to strongly relate to an infiltrating level of an immune cell type as previous did.

### 2.10. Functional Exploration of Hub Genes

Gene set enrichment analysis (GSEA) might have researchers to understand the role of genes in biological behaviors. To explore the biological functions of hub IRGs, the median value of each hub gene was calculated via TCGA-PAAD data. 177 PAADs were divided into two groups immediately (high- and low-expression groups). We selected “c2.cp.kegg.v7.4.symbols.gmt” as the annotated gene-set. After finishing these steps, we performed GSEA (http://software.broadinstitute.org/gsea/index.jsp) [36], biological pathways of nominal *P* < 0.05, |ES| > 0.6, gene size (*n*) ≥ 20 and FDR <25% were considered significant.

### 2.11. Construction of an Immune-Related Prognostic Signature

To comprehensively consider the prognostic value of hub genes, univariate Cox analysis of OS was firstly obtained to identify prognostic IRGs. *P* < 0.05 was set as the cut-off criteria to screen prognostic biomarkers. Then relying on the expression levels and regression coefficient (Coef) of prognostic biomarkers, we established an immune-related prognostic signature. Furthermore, we evaluated risk scores (RS) of PAADs as follows:

(1) $$\begin{array}{c} \text{Risk score}=\sum_{i=1}^{n}{\text{Coef}}_{i}\;\times {\text{Exp}}_{i}.\; \end{array}$$

Coef is defined as the regression coefficient of a prognostic biomarker and Exp betokens a prognostic biomarker expression level. We further evaluated the RSs of PAAD samples retrieved from TCGA-PAAD data and MMSD using the equation, in order to measure the prognostic value of the signature. PAADs were classified into two series (high- and low-risk) in all datasets, respectively, according to the median RS in each dataset. Furthermore, we obtained OS analysis with the help of R package “survival”. In addition, time-independent (1-, 3-, and 5-year) receiver operating characteristic (ROC) curves were also plotted by using R package “survivalROC” [37].

### 2.12. Cox Proportional Hazards Regression Analysis

With the aim of prognostic value of hub genes validation, the risk score assessed by this immune-related signature and other essential clinical features (gender, age, tumor grade, and pathologic stage) from TCGA-PAAD data were selected for OS univariable Cox analysis. A factor of *P* value <0.05 was identified and further selected for multivariate Cox analysis. It will be sure if the immune-related signature was independent from the rest clinical factors for predicting OS of PAADs via this analysis. Then package “forestplot” [38] in R software was used for visualization.

### 2.13. Establishing and Validating of a Nomogram

With the aim of avoiding the overfitting problem, we conducted cross-validation before nomogram construction. Then based on the immune-related prognostic index, a nomogram was constructed via R package “rms”. Calibrate curves were drawn to test the nomogram, the 45° line in which was defined as the best prediction. In addition, we calculated the consistency index (C-index) between actual probability and predicted probability to further measure the prediction effectiveness of the nomogram. Via “pROC” in R software, we also conducted ROC analysis. Furthermore, time-dependent (1-, 3-, 5-year) ROC analysis was conducted. We immediately obtained decision curve analysis (DCA) via R package “rmda” [39] to explore the clinical application value of the nomogram. Survival analysis and GSEA were also performed to explore the survival difference and lucking function of the prognostic signature.

### 2.14. Immune-Related Prognostic Signature in the Role of Anti-PD-1/L1 Immunotherapy

Two immunotherapeutic cohorts including their clinical information were collected and included in this step. For the IMvigor210 cohort which contained advanced urothelial carcinoma, we retrieved the expression data displayed as count number via http://research-pub.gene.com/imvigor210corebiologies. Relying on R package “DEseq2” [40], the count-number-based matrix was transformed into TPM values. Then totally 298 samples with intervention of atezolizumab (an anti-PD-L1 antibody) were collected and used for further exploration. Moreover, a dataset (GSE78220) [41] including metastatic melanoma samples treated with pembrolizumab (an anti-PD-1 antibody) was retrieved from GEO database. R package “limma” was used for the normalization, the FPKM value was also transformed into the TPM value. In total, 27 samples were collected and used for further exploration. The survival curves for prognostic analysis were generated by using R package “survival”. And receiver operating characteristic (ROC) curves were also drawn and the area under the curve (AUC) were further calculated by using R package “pROC”. Moreover, we also explored the association between the immune-related prognostic signature and immunocytes by using the Cell type Identification by Estimating Relative Subsets ff RNA Transcripts (CIBERSORT) (https://cibersort.stanford.edu/). In addition, by using PACA-CA data with tumor status information (primary or metastatic) retrieved from ICGC data portal (https://dcc.icgc.org/), we also explored the relationship between the immune-related signature and tumor status.

## 3. Results

### 3.1. Key Module Identification

**Figure 2:**
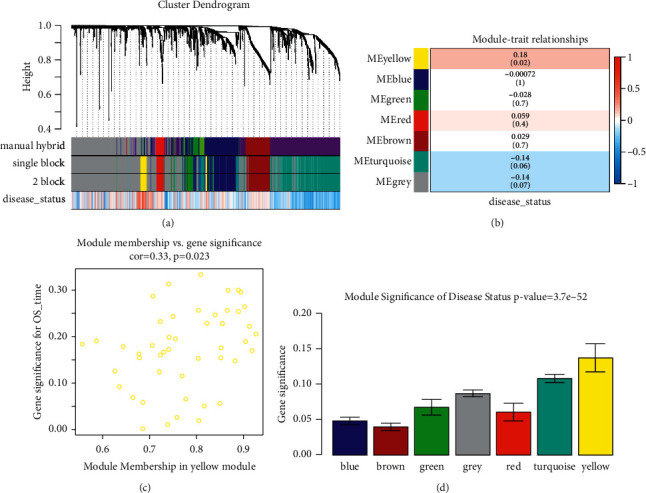
Relevant module associated with clinical information identification. (a) Dendrogram of all differentially expressed genes clustered based on a dissimilarity measure (1-TOM). (b) Heatmap of the correlation between module eigen genes and different clinical information of ccRCC (disease status). (c) Scatter plot for correlation between gene module membership in the yellow module (disease status) and gene significance. (d) Distribution of average gene significance and errors in the modules associated with disease status of PAAD.

After removing 8 outliers, totally 173 PAADs were used for WGCNA (Figure S1). In order to evaluate adjacencies, beta (*β*) = 9 (scale free *R*^2^ = 0.84) was set as the soft-thresholding power (Figure S2). IRGs were identified and further assigned to gene modules. In total, six modules were identified, which were showed in Figure 2(a). Genes with weak association with clinical trait were incorporated into the grey module and further removed in the present study. Furthermore, yellow module was chosen among the six modules because of the most positive correlation with disease status (*P*=0.02, *R*^2^ = 0.18, Figure 2(b)). As Figure 2(c) suggested, MM and GS of the yellow module (cor = 0.33, *P*=0.023) showed significant relationship. We also found that the MS of the yellow module was the highest compared with the rest modules (Figure 2(d)). Therefore, we considered yellow modules as key module.  Figure S3(a) showed the network heatmap based on these IRGs. In addition, the classical MDS plot (Figure S3(b)) suggested that the six modules were independent from each other.

### 3.2. DEG Screening

**Figure 3:**
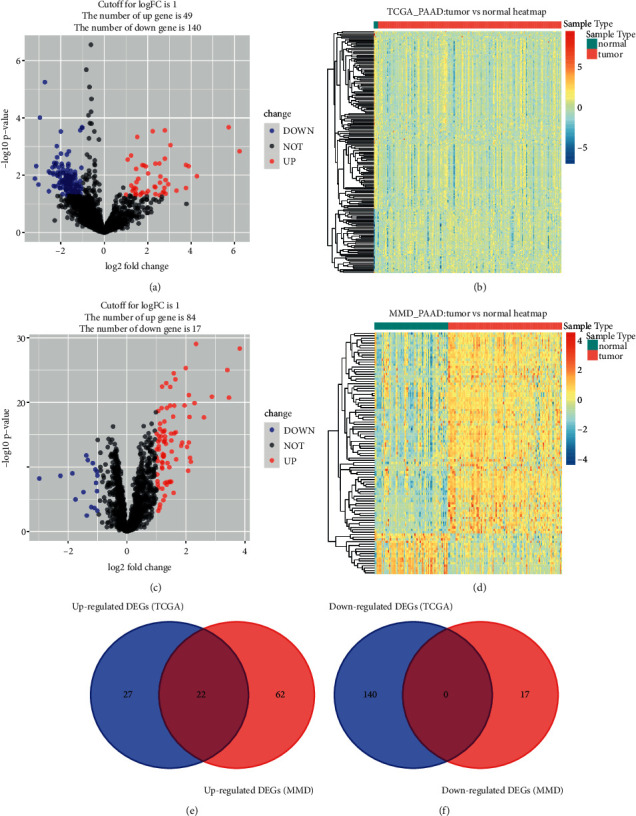
Identification of common differentially expressed IRGs. (a) Volcano plot visualizing differentially expressed IRGs in TCGA-PAAD data. (b) Heatmap of differentially expressed IRGs between tumor samples vs normal samples (*P* < 0.05, fold change >1, TCGA-PAAD). (c) Volcano plot visualizing differentially expressed IRGs in MMD. (d) Heatmap of differentially expressed IRGs between tumor samples vs normal samples (*P* < 0.05, fold change >1, MMD). (e) Identification of common upregulated differentially expressed IRGs. (f) Identification of common downregulated differentially expressed IRGs.

**Figure 4:**
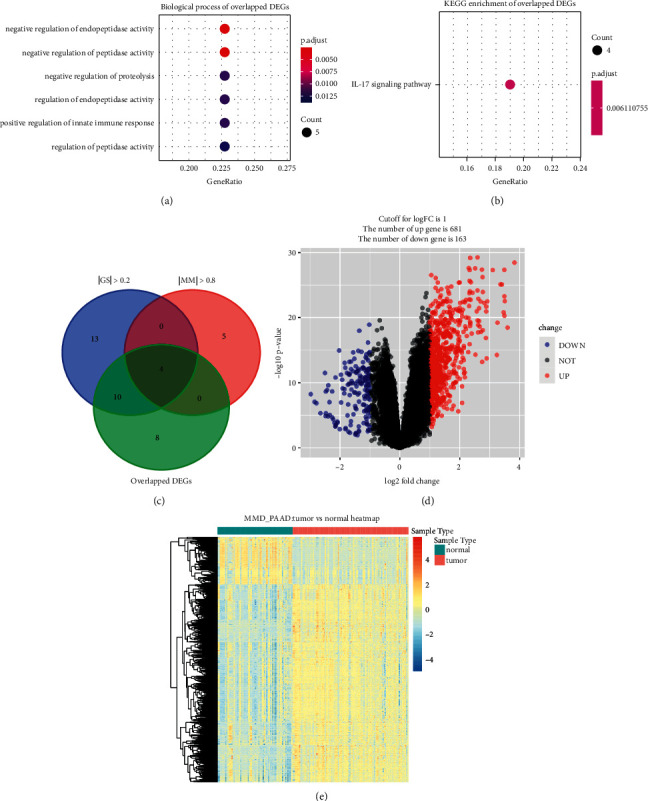
Bioinformatics analysis of genes based on 22 IRGs and identification of differentially expressed genes (DEGs). (a) GO biological processes analysis. (b) KEGG pathway enrichment. (c) Identification of hub genes by overlapping hub genes in the coexpression network and differentially expressed IRGs. (d) Volcano plot visualizing DEGs in MMD. (e) Heatmap of DEGs between tumor samples vs normal samples (*P* < 0.05, fold change >1).

Based on the cut-off criterion we set, totally 189 DEGs (49 up-regulated IRGs and 140 down-regulated IRGs) were screened which were abnormally expressed in TCGA-PAAD data (Figures 3(a) and 3(b)). In addition, 101 DEGs (84 overexpressed and 17 low-expressed) were screened out using MMD (Figures 3(c) and 3(d)). Finally, 22 DEGs overlapped between TCGA-PAAD based DEGs and MMD based DEGs were screened out for subsequent analysis (Figures 3(e) and 3(f)).  Table S1 showed the detail information of different expressed IRGs. We further conducted GO and KEGG pathway analysis for functional exploration. As shown in Figure 4(a), GO analysis indicated that IRGs were involved in negative regulation of endopeptidase activity, negative regulation of peptidase activity, negative regulation of proteolysis, regulation of endopeptidase activity, positive regulation of innate immune response, and regulation of peptidase activity. Furthermore, the selected IRGs were significantly associated with IL-17 signaling pathway, suggested by KEGG enrichment analysis (Figure 4(b)).

### 3.3. Hub Gene Identification

Four hub genes with |cor.geneModuleMembership| >0.8 and |cor.geneTraitSignificance| >0.2 were identified. All this biomarkers in WGCNA belonged to the 22 overlapped DEGs. Thus, the four genes including S100 calcium binding protein A6 (S100A6), S100 calcium binding protein A10 (S100A10), S100 calcium binding protein A16 (S100A16), and syndecan 1 (SDC1) were concluded to be hub genes in this study, which were regarded as candidate prognostic biomarkers for further validation (Figure 4(c)).

### 3.4. Novel Choices for PAAD Treatment

In order to provide some drugs to treat PAAD, we also performed CMap analysis. DEGs were firstly screened out by using MMD. Totally, 844 DEGs (681 up-regulated and 163 down-regulated) were screened out (Figures 4(d) and 4(e)). The detail information of each DEG was detailed in Table S2. We subsequently conducted CMap via these DEGs and further screened out seven molecule drugs (Table 2). Among them, sulpiride (*P*=0.00008), famotidine (*P*=0.00008), and nalidixic acid (*P*=0.0118) showed strong potential to treat PAAD.

### 3.5. Multilayered Validation of Hub Genes

**Figure 5:**
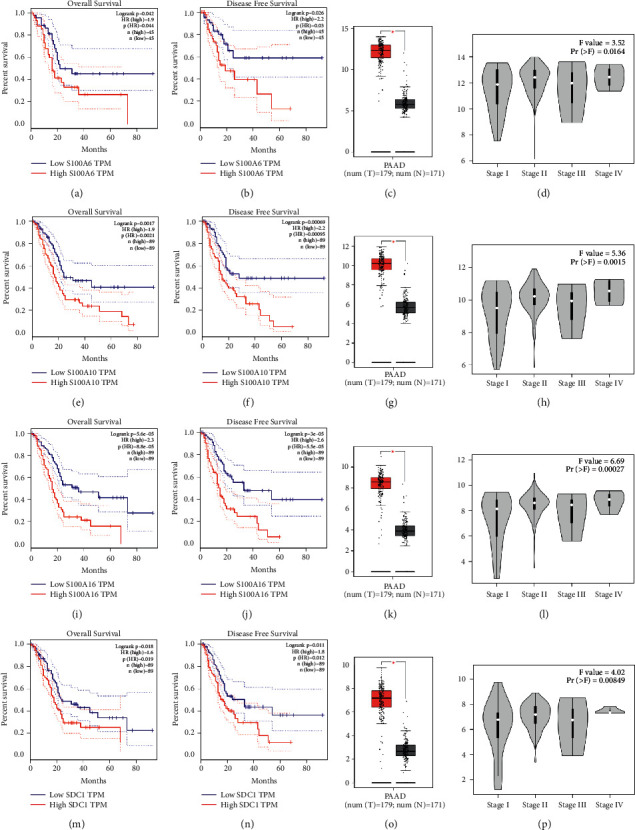
Validation of hub genes. Kaplan–Meier survival curve based on GEPIA database revealed that PAAD patients with higher expression of hub genes had a significantly shorter overall survival time (S100A6: A; S100A10: E; S100A16: I; SDC1: (m)) and disease free survival time (S100A6: B; S100A10: F; S100A16: J; SDC1: (n)). Expressions of S100A6 (c), S100A10 (g), S100A16 (k), and SDC1 (o) in PAAD were significantly higher than these in normal tissues based on TCGA-PAAD database (*P* < 0.05). High expression of S100A6 (d), S100A10 (h), S100A16 (l), and SDC1 (p) related to higher tumor stage.

**Figure 6:**
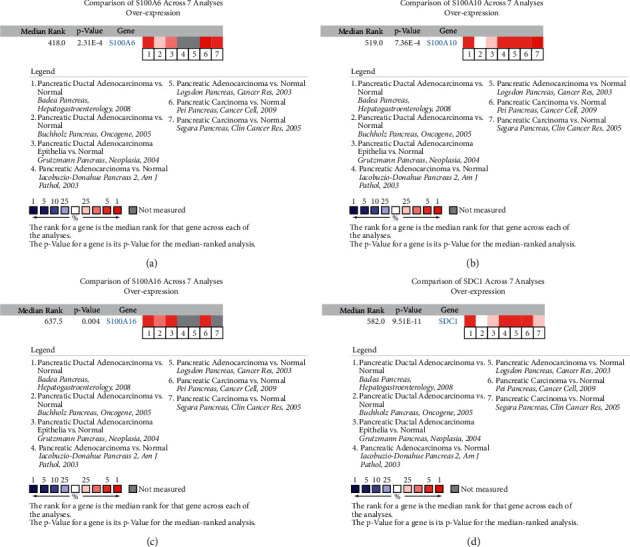
Oncomine database analyses. (a) Comparison of S100A6 mRNA expression across 7 analyses of PAAD based on Oncomine database. (b) Comparison of S100A10 mRNA expression across 7 analyses of PAAD based on Oncomine database. (c) Comparison of S100A16 mRNA expression across 7 analyses of PAAD based on Oncomine database. (d) Comparison of SDC1 mRNA expression across 7 analyses of PAAD based on Oncomine database.

**Figure 7:**
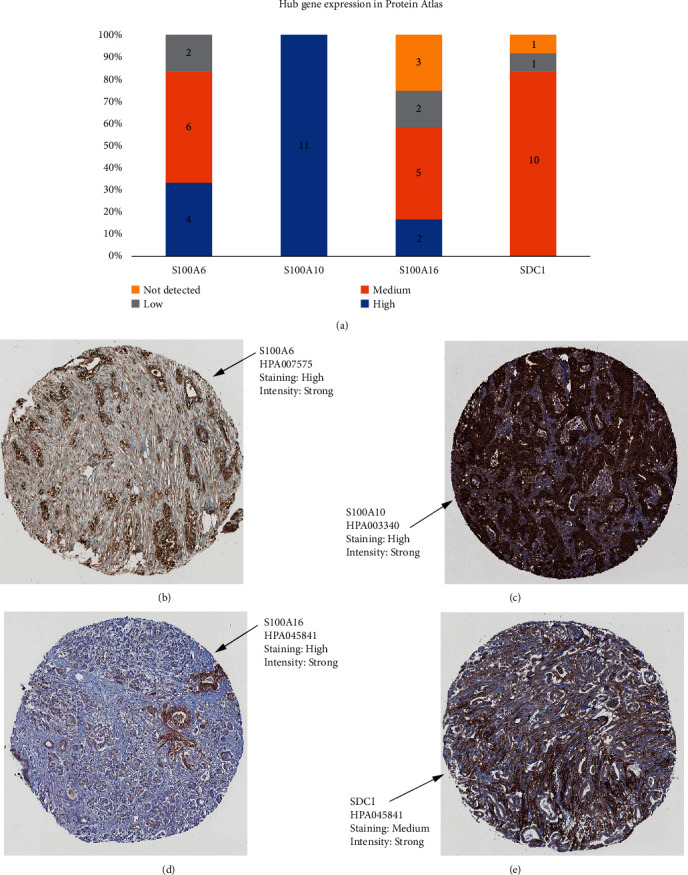
Detection of hub gene expression by immunochemistry in human pancreatic cancer human tissue. (a) Histogram of mucin expression in PDAC samples from Protein Atlas (http://www.proteinatlas.org/). In total, 11–12 samples were analyzed for S100A6, S100A10, S100A16, and SDC1. Immunohistochemistry (IHC) staining was evaluated as high/medium/low staining or not detected. (b) Representative IHC stainings for S100A6. (c) Representative IHC stainings for S100A10. (d) Representative IHC stainings for S100A16. (e) Representative IHC stainings for SDC1. All hub genes showed a membrane and/or cytoplasmic staining in tumor cells.

**Figure 8:**
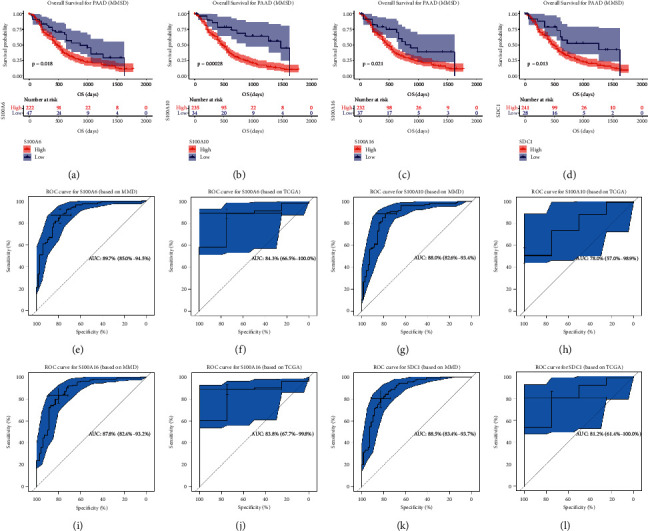
Validation of survival and prognostic value of hub genes. Kaplan–Meier overall survival (OS) curves for PAAD patients assigned to groups of high and low-expression level of based on the S100A6 (a), S100A10 (b), S100A16 (c), and SDC1 (d), respectively. (e) ROC curve for S100A6 based on TCGA-PAAD (AUC = 0.897). (f) ROC curve for S100A6 based on MMD (AUC = 0.843). (g) ROC curve for S100A10 based on TCGA-PAAD (AUC = 0.826). (h) ROC curve for S100A10 based on MMD (AUC = 0.780). (i) ROC curve for S100A16 based on TCGA-PAAD (AUC = 0.878). (j) ROC curve for S100A16 based on MMD (AUC = 0.838). (k) ROC curve for SDC1 based on TCGA-PAAD (AUC = 0.885). (l) ROC curve for SDC1 based on MMD (AUC = 0.812).

In order to make these results more reliable, we conducted comprehensive validation for the 4 hub genes. First of all, expression of S100A6 could effectively affect the OS (hazard ratio (HR) = 1.9, *P*=0.0142) and DFS (HR = 2.2, *P*=0.026) of PAAD (Figures 5(a) and 5(b)) via GEPIA. Moreover, higher expression of S100A10 was concluded to show association with short OS time (HR = 1.9, *P*=0.0017) and DFS time (HR = 2.2, *P*=0.00069), suggested by Figures 5(e) and 5(f). Besides, survival analysis indicated that S100A16 expression of PAAD patients was negatively associated with OS time (HR = 2.3, *P*=5.6E − 05, Figure 5(i)) and DFS time (HR = 2.6, *P*=3E − 05, Figure 5(j)), significantly. Also, we concluded that PAAD patients with higher SDC1 expression occupied poorer OS (HR = 1.6, *P*=0.018) and DFS (HR = 1.8, *P*=0.012), accurately (Figures 5(m) and 5(n)). The expression differences of the 4 hub genes were further explored. As we expected, S100A6 (Figure 5(c)), S100A10 (Figure 5(g)), S100A16 (Figure 5(k)), and SDC1 (Figure 5(o)) were upregulated in PAADs comparing with normal tissues. Furthermore, we also concluded that down-regulated of S100A6 (*F* = 3.52, *P*=0.0164; Figure 5(d)), S100A10 (*F* = 5.36, *P*=0.0015; Figure 5(h)), S100A16 (*F* = 6.69, *P*=0.00027; Figure 5(l)), and SDC1 (*F* = 4.02, *P*=0.00849; Figure 5(p)) were effectively related to lower tumor stage. Furthermore, we compared the expression differences of hub genes by using Oncomine database. The results suggested the same conclusion that S100A6 (*P*=2.31E − 04, Figure 6(a)), S100A10 (*P*=7.36E − 04, Figure 6(b)), S100A16 (*P*=0.004, Figure 6(c)), and SDC1 (*P*=9.51E − 11, Figure 6(d)) were higher expressed in PAAD tissues compared with normal samples. With the help of HPA database, the translational-level expression of hub genes were explored, which contained 11 to 12 different PAAD samples (Figure 7(a)). We observed strong or medium staining for hub genes (Figures 7(b)–7(e)), which meant that hub genes were all higher expressed in PAAD samples. The prognostic value of hub genes was further validated after verification of expression level. As the results suggested, higher expression of S100A6 was significantly correlated to worse OS, suggested by Figure 8(a) (*P*=0.018, MMSD). In addition, PAAD patients of lower S100A10 expression occupied longer OS time (*P*=0.00028, MMSD, Figure 8(b)). Furthermore, S100A16 also showed the similar relationship with OS as S100A10 did (Figure 8(c), *P*=0.021, MMSD). Similarly, the results concluded that PAAD patients of higher SDC1 expression was significantly related to short OS time (*P*=0.013, MMSD, Figure 8(d)).

Besides, we also conducted ROC analysis. S100A6 exhibited excellent diagnostic efficiency for PAAD (MMD: AUC = 0.897, Figure 8(e); TCGA: AUC = 0.843, Figure 8(f)). S100A10 could also screen out PAAD samples from normal samples, as Figure 8(g) (AUC = 0.880) and Figure 8(h) (AUC = 0.780) showed. S100A16 also showed excellent diagnostic efficiency for PAAD (MMD: AUC = 0.878, Figure 8(i); TCGA: AUC = 0.838, Figure 8(j)). Moreover, Figure 8(k) (AUC = 0.885) and 8(l) (AUC = 0.812) indicated that SDC1 showed great potential for diagnosis. All the results above indicated that the four hub genes might be novel immune-related prognostic biomarkers.

### 3.6. Hub Genes Were Correlated to Immune-Related Pathways

GSEA concluded that S100A6 was obviously associated with three KEGG signaling pathways containing Homologous recombination (*P*=0.002, |ES| = 0.630, *n* = 26, FDR = 13.548%), Pentose and glucuronate interconversions (*P*=0.029, |ES| = 0.652, *n* = 28, FDR = 13.965%), and Ascorbate and aldarate metabolism (*P*=0.042, |ES| = 0.659, *n* = 25, FDR = 14.815%) (Table 3). Meanwhile, we found that S100A10 was significantly associated with DNA replication, Pentose phosphate pathway, and Homologous recombination (Table 3). Furthermore, S100A16 was significantly enriched in Pentose and glucuronate interconversions, Homologous recombination, DNA replication, and Mismatch repair (Table 3). As for SDC1, this hub gene was associated with P53 signaling pathway, significantly (Table 3).

### 3.7. Correlation of Hub Gene Expression with Immune Infiltration Level in PAAD

**Figure 9:**
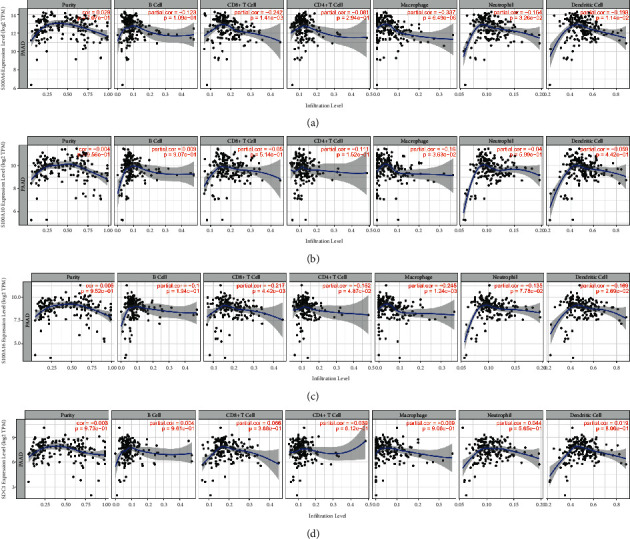
(a) Correlation of S100A6 expression with immune infiltration level in PAAD. (b) Correlation of S100A10 expression with immune infiltration level in PAAD. (c) Correlation of S100A16 expression with immune infiltration level in PAAD. (d) Correlation of SDC1 expression with immune infiltration level in PAAD.

Immune infiltration was reported to be associated with survival and progression of cancers. Thus, by using TIMER (a webtool), the association between hub genes and immune infiltration level was obtained. S100A6 was negatively related to CD8+ T cells (cor = −0.242, *P*=1.41*E* − 03, Figure 9(a)) meanwhile negatively correlated to macrophage (cor = −0.337, *P*=6.49*E* − 06, Figure 9(a)). S100A16 was negatively correlated to CD8+ T cells (cor = −0.217, *P*=4.42*E* − 03) and macrophage (cor = −0.245, *P*=1.24*E* − 03), suggested by Figure 9(c). Unfortunately, we found expressions of S100A10 (Figure 9(b)) and SDC1 (Figure 9(d)) had no significant relationship with immune infiltration level of the six immune cell types.

### 3.8. Establishing an Immune-Related Prognostic Signature

**Figure 10:**
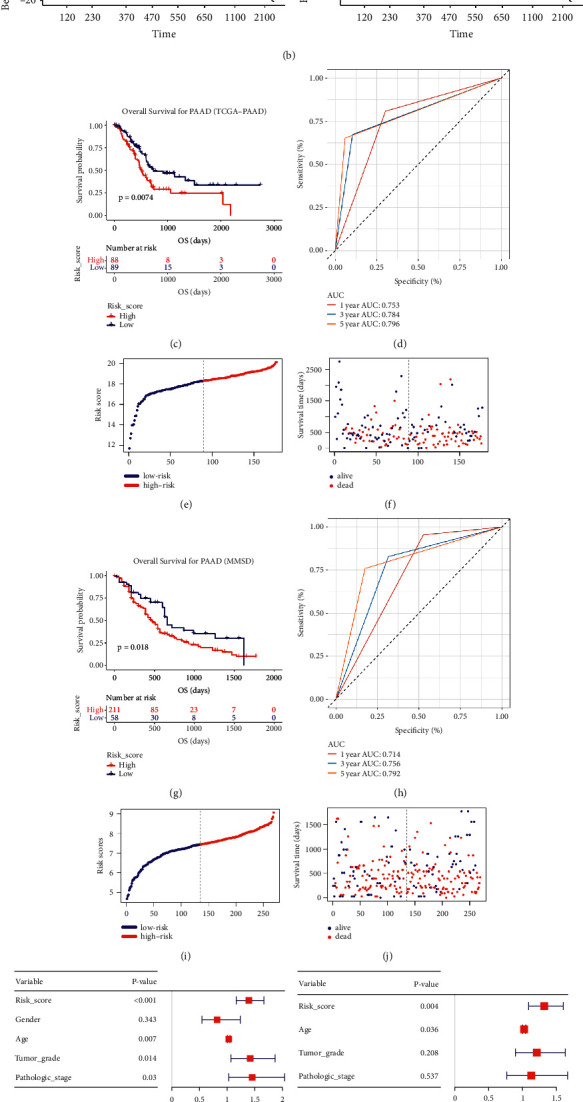
Construction of a novel immune-related prognostic signature and Cox regression analysis. (a) Univariate Cox regression analysis of the four hub genes. (b) Schoenfeld individual test for investigating the proportional hazards assumption in Cox model (S100A6, S100A10, S100A16, and SDC1). (c) Kaplan–Meier OS curves for the high- and low-risk groups by using TCGA-PAAD data. (d) ROC curve indicating the predictive accuracy of the immune-related prognostic signature for OS by using TCGA-PAAD data. (e) Distribution of the risk scores of BC patients based on TCGA-PAAD data. (f) The number of survivors and nonsurvivors with different risk scores based on TCGA-PAAD data; red represents the number of nonsurvivors, and blue represents the number of survivors. (g) Kaplan–Meier OS curves for the high- and low-risk groups by using MMSD data. (h) ROC curve indicating the predictive accuracy of the immune-related prognostic signature for OS by using MMSD. (i) Distribution of the risk scores of PAAD patients based on MMSD. (j) The number of survivors and nonsurvivors with different risk scores based on MMSD; red represents the number of nonsurvivors, and blue represents the number of survivors. (k) Forest plot summary of analyses of OS univariate analysis of Risk score, gender, age, tumor grade and pathologic stage by using TCGA-PAAD data. (l) Forest plot summary of analyses of OS multivariate analysis of Risk score, age, tumor grade and pathologic stage by using TCGA-PAAD data. (m) Schoenfeld individual test for investigating the proportional hazards assumption in Cox model (age, tumor grade, pathologic stage, and risk score).

Univariate Cox analysis of OS of these four IRGs was preliminary conducted (Figure 10(a)). By using Coxph function in R package “survival”, we conducted Schoenfeld individual test for investigating the proportional hazards assumption. The global Schoenfeld test showed no significance (*P*=0.8454, Figure 10(b)). Also, each variable including S100A6 (*P*=0.3039), S100A10 (*P*=0.5016), S100A16 (*P*=0.6219), and SDC1 (*P*=0.6655) owned no significance (*P* > 0.05, Figure 10(b)). Thus, this Cox model was conformed to the proportional hazards assumption. After this, all the prognostic biomarkers including S100A6, S100A10, S100A16, and SDC1 were used to establish the risk signature. The risk score of a sample was evaluated using the following equation:

Risk score = 0.248 × Exp_S100A6_ + 0.355 × Exp_S100A10_ + 0.404 × Exp_S100A16_ + 0.240 × Exp_SDC1_.  Table S3 contained the risk scores of all TCGA-PAAD samples. 177 PAADs were split into two groups (high-risk group (*n* = 88), low-risk group (*n* = 89)) by setting the median risk score as cut-off standards. Next process concluded that PAAD patients with low-risk scores owned longer OS (Figure 10(c), *P*=0.0074). Furthermore, the ROC values of this risk system were shown in Figure 10(d) (1 year: 0.753, 3 years: 0.784, 5 years: 0.796). By visualizing the distribution of patients in the two different groups (Figures 10(e) and 10(f)), we found that patients in high-risk groups were more likely to die comparing with these in low-risk group. With the aim of validating the repeatability and applicability of this signature, we repeated what we did in TCGA data via MMSD data, Table S4 contained the RSs of all PAADs. We also classified PAADs into high- (*n* = 134) and low-risk group (*n* = 135). PAADs who had higher-risk scores owned obviously poorer OS (Figure 10(g), *P*=0.018), which was consistent with the previous result. The predictive values of this signature for 1-, 3- and 5-years were quantified as 0.714, 0.756 and 0.792 via MMSD, accurately (Figure 10(h)). Figures 10(i) and 10(j) concluded the similar conclusion as TCGA-PAAD told.

### 3.9. A Nomogram with Clinical Utility Was Constructed Based on the Immune-Related Prognostic Signature

**Figure 11:**
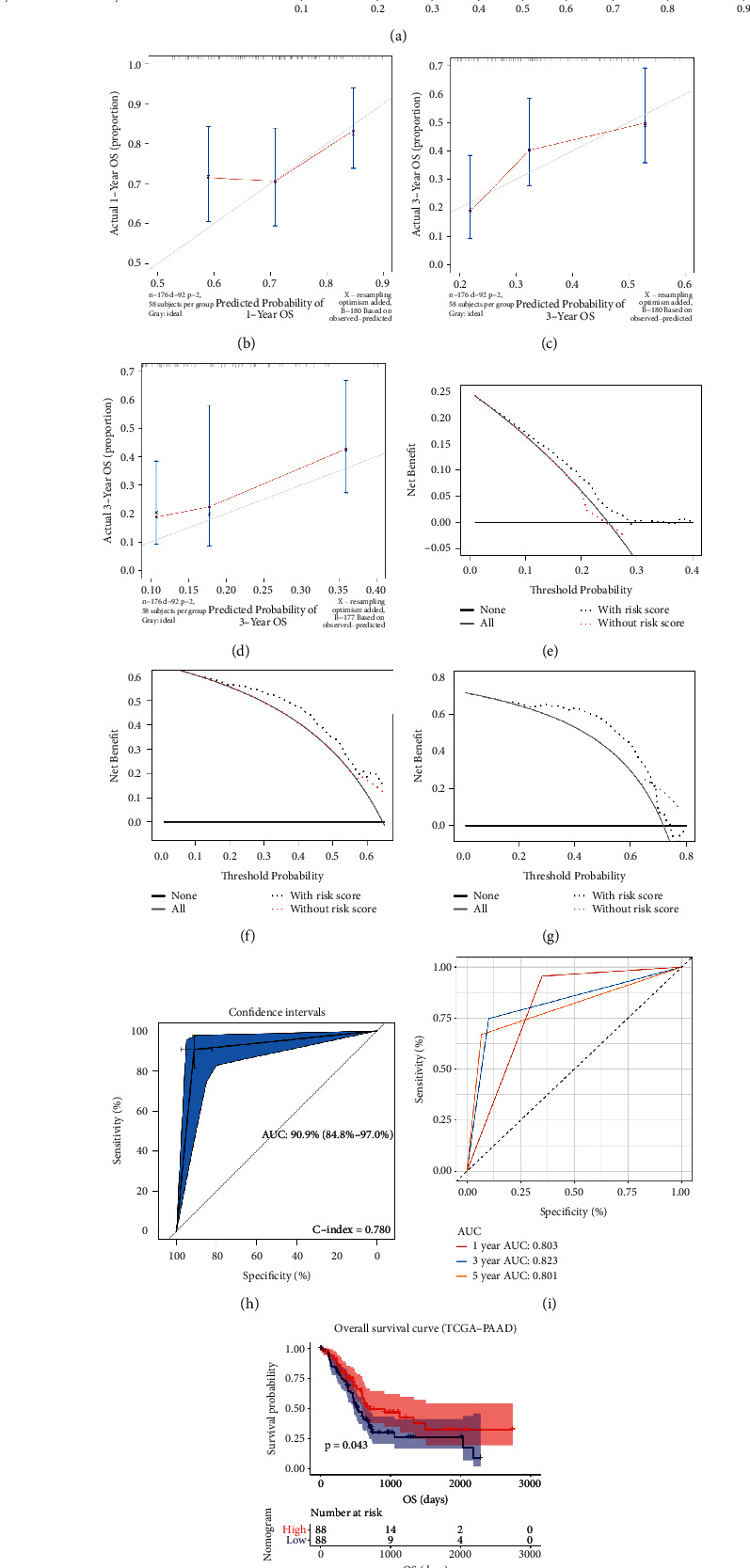
(a) The nomogram constructed with the immune-related prognostic signature for predicting proportion of patients with 1-, 3- or 5-year OS. The calibration plots for predicting 1- (b), 3- (c), or 5- (d) year OS. DCA for assessment of the clinical utility for 1- (e), 3- (f), or 5- (g) year OS of the immune-related prognostic signature, the *x*‐axis represents the percentage of threshold probability, and the *y*‐axis represents the net benefit. OS: overall survival; DCA: decision curve analysis. (h) Receiver operating characteristic (ROC) curves and area under the curve (AUC) statistics to evaluate the diagnostic efficiency of the nomogram based on the immune-related prognostic signature in TCGA-PAAD data. (i) Time-dependent ROC curves indicating the predictive accuracy of the nomogram based on the immune-related prognostic signature for 1-, 3-, or 5- year OS based on TCGA-PAAD data. (j) Survival analysis of the association between risk score calculated by the immune-related prognostic signature and overall survival time in PAAD using TCGA-PAAD data.

As the univariable Cox analysis suggested, risk score (*P* < 0.001), age (*P* < 0.007), tumor grade (*P* < 0.014), and stage (*P*=0.030) were significantly associated with OS (Figure 10(k)). Subsequent multivariate Cox analysis confirmed that the risk score could predict the prognosis of PAAD patients as individual (Figure 10(l)). Schoenfeld individual test was further performed for investigating the proportional hazards assumption as previous did. The global Schoenfeld test showed no significance (*P*=0.107, Figure 10(m)). Also, each variable including age (*P*=0.6687), tumor grade (*P*=0.2792), pathologic stage (*P*=0.0706), and risk score (*P*=0.3409) was not statistically significant (*P* > 0.05, Figure 10(m)). Thus, this Cox model was conformed to the proportional hazards assumption. With the aim of applying clinical practice of this signature, we further constructed a nomogram based on risk score and age (Figure 11(a)), which showed significance in multivariate Cox analysis (Figure 10(k)). As the calibrate curve concluded, our nomogram could effectively predicate the survival rate of PAAD patient (Figures 11(b)–11(d)), least of all from long-term mortality (3-year OS (Figure 11(c)); 5-year OS (Figure 11(d)). Clinical net benefit of this nomogram was measured by DCA. The results suggested that the risk-signature-based nomogram owned better net benefit comparing to the no-risk-score nomogram when predicting 1- (0.10 < Pt < 0.30, Figure 11(e)), 3- (0.20 < Pt < 0.65, Figure 11(f)), 5-year (0.20 < Pt < 0.65, Figure 11(g)) survival probability. By drawing ROC curves, the nomogram was validated to show great potential for PAAD patients OS prediction (C-index: 0.780; AUC: 0.909; Figure 11(h)). Subsequent analysis concluded that this nomogram with immune-related prognostic index revealed great steadiness across five years. The AUCs of 1-, 3-, and 5-year were 0.803, 0.823, 0.801, respectively (Figure 11(i)). As the survival analysis told, a PAAD patient who owned higher nomogram would get better OS (*P*=0.043, Figure 11(j)).

### 3.10. Identification of the Risk Signature Associated KEGG Signaling Pathways

**Figure 12:**
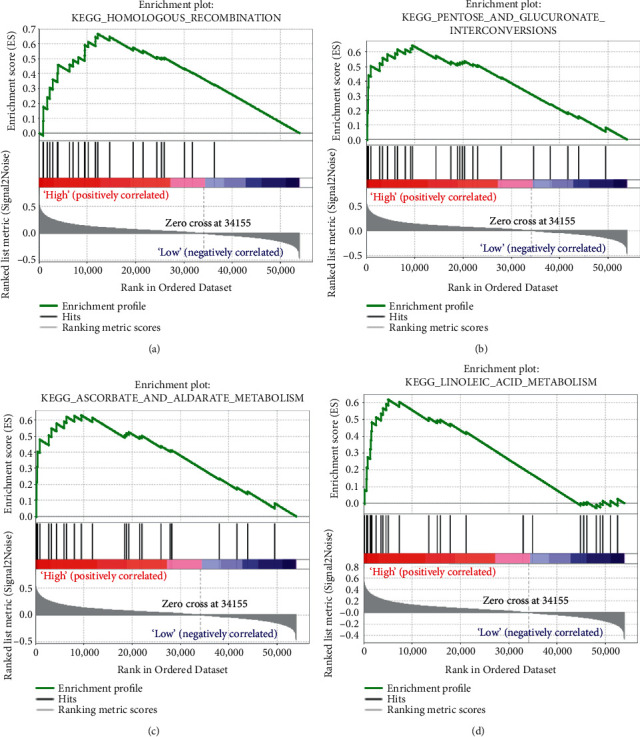
Geneset enrichment analysis (GSEA) of the immune-related prognostic signature. (a) Homologous recombination. (b) Pentose and glucuronate interconversions. (c) Ascorbate and aldarate metabolism. (d) Linoleic acid metabolism.

We further conducted GSEA to screen the abilities of the established index. Based on the standards we set in methods part, the risk score played significant role in Homologous recombination (Figure 12(a)), Pentose and glucuronate interconversions (Figure 12(b)), Ascorbate and aldarate metabolism (Figure 12(c)), and Linoleic acid metabolism (Figure 12(d)). The detail information for these pathways were shown in Figures 12(a)–12(d).

### 3.11. Role of the Immune-Related Prognostic Signature in Immunotherapeutic Benefit Prediction

**Figure 13:**
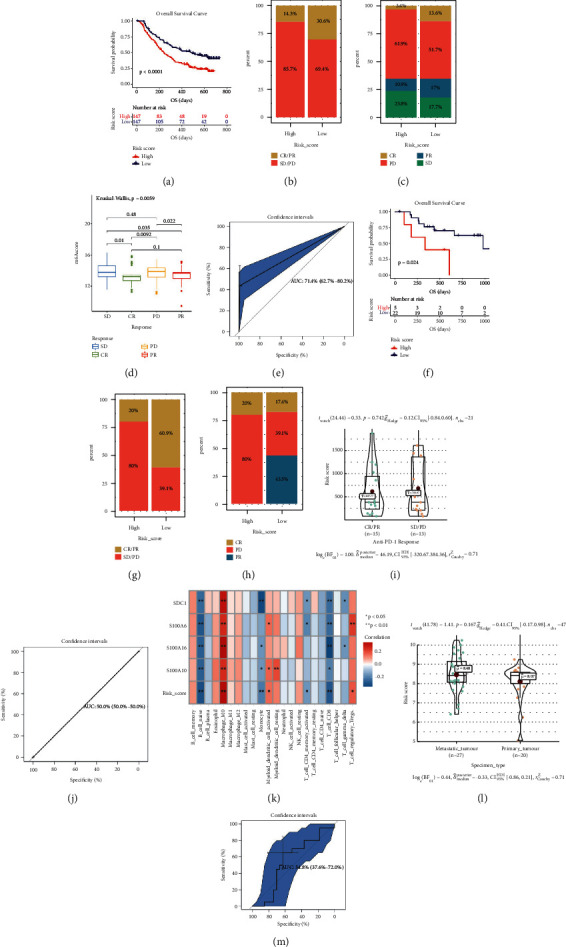
Risk score is a prognostic biomarker and predicts immunotherapeutic benefit. (a) Kaplan–Meier curves for patients with high (*n* = 147) and low (*n* = 147) risk score in the IMvigor210 cohort. (b) Rate of clinical response (complete response (CR)/ partial response (PR) and stable disease (SD)/progressive disease (PD)) to anti-PD-L1 immunotherapy in high or low-risk score groups in the IMvigor210 cohort. (B) Rate of clinical response (complete response (CR), partial response (PR), stable disease (SD), and progressive disease (PD)) to anti-PD-L1 immunotherapy in high or low-risk score groups in the IMvigor210 cohort. (d) Distribution of risk score in groups with different anti-PD-L1 clinical response statuses. (e) ROC curve measuring the predictive value of the risk score. (f) Kaplan–Meier curves for patients with high (*n* = 5) and low (*n* = 22) risk score in the GSE78220 cohort. (g) Rate of clinical response (complete response (CR)/ partial response (PR) and stable disease (SD)/progressive disease (PD)) to anti-PD-1 immunotherapy in high or low-risk score groups in the GSE78220 cohort. (h) Rate of clinical response (complete response (CR), partial response (PR), stable disease (SD), and progressive disease (PD)) to anti-PD-1 immunotherapy in high or low-risk score groups in the GSE78220 cohort. (i) Distribution of risk score in groups with different anti-PD-1 clinical response statuses. (j) ROC curve measuring the predictive value of the risk score. (k) The relationships between risk score and 22 immune cell types. (l) Risk score level comparison between metastatic PAAD and primary PAAD. (m) ROC curve measuring the predictive value of the risk score.

Immunotherapies (including PD-L1, PD-1 blockade, and etc) has undoubtedly emerged a major breakthrough in tumor treatment. In IMvigor210 (Table S5 showed the RSs of patients from IMvigor210), PAAD patients classified into high-risk score group (*n* = 147) owned significantly shorter survival (*P* < 0.001, Figure 13(a)), compared with patients in low-risk score group (*n* = 147). The risk score was further proved to show good potential for anti-PD-L1 immunotherapy predication by using IMvigor210 (Figures 13(b)–13(d)). Patients in lower risk score group were more likely to benefit from anti-PD-L1 treatment (Figures 13(b) and 13(c)), which was validated by Kruskal-Wallis test (*P*=0.0059, Figure 13(d)). Next ROC analysis (based on IMvigor210) was used to evaluate the efficacy of anti-PD-L1 treatment by risk score, which recommended that risk score was a predictive biomarker of immunotherapeutic benefits (AUC: 0.714, Figure 13(e)). In addition, we also tried to find some relationship between risk score and anti-PD-1 treatment via GSE78220 cohort. The results suggested that PAAD patients in low-risk group exhibited significantly clinical benefits and an obviously prolonged survival (*P*=0.024, Figure 13(f)). Also, patients in lower risk score group were more likely to benefit from anti-PD-1 treatment (Figures 13(g) and 13(h)). There was a trend that patients with response (CR/PR) to anti-PD-1 treatment occupied lower risk score (*P*=0.742, Figure 13(i)). But unfortunately, further ROC analysis also demonstrated that risk score might not be an appropriate predictive tool to anti-PD-1 treatment benefits (AUC: 0.500, Figure 13(j)).

### 3.12. Correlation of Immune-Related Prognostic Signature with Immune Infiltration Level, And Tumor Status in PAAD

Hopkins et al. proved T cell receptor repertoire features might act as a novel biomarker for PAAD immunotherapy response [42]. Thus, we also tried to explore the association among our index and immune cells. According to Figure 13(k), the signature was correlated to B cell naïve, monocyte, CD4+ T cell memory activated and CD8+ T cell, negatively. Meanwhile positively correlated to macrophage M0, myeloid dendritic cell activated, and T cell regulatory (Tregs). The *P* values were shown in Figure 13(k). Moreover, because of the present situation that the uncertainty of prediction of T cell receptor (TCR) might to immunotherapy in metastatic cancer patients [43], we also attempted to explore whether the immune-related signature could predict it was a metastatic tumor or not. As the results showed (Figure 13(l)), metastatic PAAD occupied higher-risk score compared with primary PAAD. But the result must be validated by using datasets with larger samples because the *P* value was greater than 0.05. Further ROC analysis also demonstrated that the immune-related prognostic signature might not effectively distinguish metastatic PAAD from primary PAAD (AUC: 0.548, Figure 13(m)).

## 4. Discussion

Pancreatic adenocarcinoma carries the lowest survival rate of all major organ cancers, which is of dismal prognosis and high mortality rate [44]. Most PAAD patients have no symptoms even in the advanced stage. Surgical management is the only treatment possible to cure PAAD, but its results is not satisfactory by now [44]. The 5-year survival rate of PAAD patients after receiving complete surgical resection is still less than 25% [45]. PAAD, with an insidious and atypical clinical symptoms, is a malignant neoplasm of the digestive tract that is difficult to diagnose and treat. PAAD in its early stages is not associated with a high rate of surgical mortality, whereas cure rates are low. Obviously, there was of great need of effective therapy target and prognostic biomarkers. Therefore, screening novel prognostic biomarkers for PAAD and providing some novel choices for PAAD therapy are of urgent need.

Cancer immunotherapy is an important tumor therapy potion for prevention and treatment of tumors and has attracted tremendous interests [46]. Nowadays, more and more researchers focus on screening out novel prognostic biomarkers related to immune microenvironment. However, similar studies regarding PAAD remains scarce. Thus, the aiming of the present study is to identify some biomarkers associated with prognosis of PAAD and explore some molecule drugs with therapeutic effect for PAAD.

Angelo et al. firstly investigated the correlation between PAAD patient prognosis and expression of 521 immune system genes [47]. Twenty immune system genes were carried out, which might influence PAAD prognosis. But there was a lack of validation for the 20 genes, and no further breakthrough had been made. Wu et al. had established a 3 IRG-based index, which showed definitive predictability for PAAD [48]. But not as we expected, the AUC for this model was less than 0.75, which might not be an effective tool for survival prediction. Chen et al. developed a two DEIRG-based signature, which could be used as an independent tool for the prognostic prediction of PAAD and to provide potential novel immunotherapy targets [49]. The similar question was that the AUC not as high as we expected (OS: 0.736) [49]. To learn from advantages and avoid disadvantages of these studies, we attempted to explored an immune-related prognostic signature via several authoritative methods and strict thresholds, based on multiple datasets and databases.

For the first time, we conducted WGCNA by using IRGs collected from ImmPort database. Four hub genes including S100A6, S100A10, S100A16, and SDC1 were screened out from the genes in the key module (yellow module). In addition, 22 overlapped DEGs between DEGs identified by MMD and EDGs identified by TCGA-PAAD were also selected. It was coincidence that the four hub genes identified by WGCNA were all the genes in the 22 DEGs. Thus, we regarded the four IRGs as potential prognostic biomarkers in PAAD and further verified them at different levels. All the four IRGs were overexpressed in PAAD tissues, compared with normal tissues, suggested by TCGA-PAAD data, MMD, GEPIA, and Oncomine database. Previous studies also indicated that the four IRGs were higher expressed in some cancer types. S100A6 has been proved to be highly expressed in epithelial cells, fibroblasts, and some cancer cell types, function of which was still unclear [50, 51]. Serum S100A6 level was significantly increased in some solid carcinomas, including gastric carcinoma [52], bladder carcinoma [53] and ovarian carcinoma [54]. S100A10 was highly expressed in gastric cancer compared with normal gastric mucosa tissues, which was involved in the occurrence and development of gastric cancer [55]. S100A16 was overexpressed in lung cancer [56], and colorectal cancer [57], which might play role in promoting the proliferation and migration of tumors. SDC1 was a kind of heparan sulphate proteoglycan, which was an important cell surface adhesion molecule to maintain cell morphology [58]. As reported, the disorder of SDC1 expression could effectively influence tumor cells invasion and metastasis [59]. S100A6, S100A10, and S100A16 are members of the S100 family of proteins containing 2 EF-hand calcium-binding motifs [60]. Some protein types encoded by S100 family have been used as tumor markers in clinical [60]. In conclusion, this study suggested that the expressions of S100A6, S100A10, S100A16, and SDC1 were upregulated in PAAD, which might be crucial biomarkers in the progression of PAAD.

Furthermore, we validated the prognostic value of the four IRGs by using TCGA-PAAD data and MMSD. The results conducted that higher expression of the four IRGs were related to worse survival (OS and DFS) of PAADs. To learn from advantages and avoid disadvantages of these studies, we attempted to explored an immune-related prognostic signature via several authoritative methods and strict thresholds, based on multiple datasets and databases. Previous studies also indicated that over expression of serum S100A6 level was closely associated with the occurrence, development, prognosis and treatment of tumors [52]. It also had been reported that the up-regulation of S100A10 in renal cell carcinoma and bladder carcinoma was closely related to the poor prognosis, which might be correlated with the promotion of tumor cell proliferation, migration and invasion by S100A10 [61]. The disorder of SDC1 expression was also significantly correlated to poor prognosis of cancers [62]. As a conclusion, the present study supported the view that the four IRGs were closely associated with prognosis of tumors and might be novel immune-related prognosis biomarkers in PAAD.

Moreover, we established an immune-related prognostic signature base on the four prognostic biomarkers. It was worth mention that our immune-related signature might be the first one constructing (by combing DEG and WGCNA) for prognosis of PAAD patient prediction. This signature was validated to perform as an independent prognostic index with excellent potential of prognosis prediction of PAAD patients. With the aim of making the risk signature to become a clinical reality, we constructed a nomogram relying on the prognostic signature and age. The risk-signature-based nomogram was immediately validated to serve as a predictor for OS possibility of PAADs.

Argentiero et al. revealed that immune treatment might be effectively strategy through WNT pathway [63]. These findings hold the promise to identify novel immune-based therapeutic strategies targeting WNT to enhance PDAC cytotoxicity and restore anti-PDAC immunity in node-positive disease. Russano et al. discussed the current and possibly future applications of blood-based liquid biopsy in oncology, its advantages and its limitations in clinical practice [43]. Moreover, they specifically revealed its role as a tool to capture tumor heterogeneity in metastatic cancer patients. In another study, Hopkins et al. proved T cell receptor repertoire features associated with survival in immunotherapy-treated PAAD [42]. Whether these repertoires might predict response to immunotherapy in metastatic cancer patients is still unclear. Thus, we tried to explore the role of our signature in immunotherapeutic benefit prediction. The results obviously demonstrated that this signature was related to response to anti-PD-1/L1 immunotherapy.

Some small molecule drugs were also identified, which could be useful in the treatment of PAAD. 844 DEGs were firstly identified by using MMD developed by four independent datasets. We then performed CMap analysis and the results demonstrated that three molecule drugs including sulpiride, famotidine, and nalidixic acid might be potential choices in the treatment of PAAD.

Some limitations might exist in the present study. As a multiple-dataset-based study, though we tried our best to design this bioinformatic study reasonably and logically, this research was lack of external experimental verification. Some experiments must be conducted in our further research to measure the relayed mechanisms of hub genes for PAAD. Besides, we will also validate the therapy potential of the three drugs in the subsequent analysis. In addition, clinical trials based on the extensive genomic technologies made the findings more reliable.

## 5. Conclusions

All in all, hub genes in WGCNA was firstly screened. Then DEGs were further identified by using TCGA-PAAD data and MMD. Furthermore, four IRGs were screened out by overlapping core genes identified by WGCNA and abnormally expressed IRGs. All the four IRGs were screened out and verified to be correlated to dismal prognosis of PAAD. Moreover, 3 molecule drugs (sulpiride, famotidine, and nalidixic acid) were chosen and validated to have potential to treat PAAD. In conclusion, the present study indicated that four IRGs might be novel immune-related prognostic biomarkers of PAAD. Besides, three molecule drugs might have worked in the treatment of PAAD. An immune-related prognostic signature was constructed and validated as an independent prognostic biomarker with excellent potential of PAAD prognosis prediction. The nomogram based on the risk signature could act as a visual tool for OS probability prediction of PAADs.

## Figures and Tables

**Table 1:** Detail information of the eight datasets.

Source	Dataset	Platform	Preprocessing method	# of patient samples	# of tumor samples	# of normal samples	Clinical information
TCGA-PAAD	TCGA-PAAD	RNASeqV2	Deseq.2	181	177	4	√
GSE15471	MMD	GPL570	Affy-RMA	78	39	39	
GSE16515		GPL570	Affy-RMA	52	16	36	
GSE22780		GPL570	Affy-RMA	16	8	8	
GSE32676		GPL570	Affy-RMA	32	25	7	
GSE21501	MMSD	GPL4133	Lowess normalized	102	102	0	√
GSE28735		GPL6244	Affy-RMA	84	42	42	√
GSE71729		GPL20769	Nonnegative normalized	213	125	88	√

**Table 2:** Results of Cmap analysis based on DEGs in PAAD.

Cmap name	Mean	*n*	Enrichment	*P*	Specificity	Percent nonnull
Sulpiride	−0.569	5	−0.864	0.00008	0	100
Famotidine	−0.566	5	−0.859	0.00008	0	100
6-Bromoindirubin-3′-oxime	−0.465	7	−0.727	0.00026	0.014	71
Nalidixic acid	−0.51	5	−0.656	0.0118	0.0108	80
Picotamide	−0.463	5	−0.642	0.01466	0.0167	80
Sulfaguanidine	−0.326	5	−0.632	0.01746	0.043	60
Guaifenesin	−0.346	6	−0.621	0.00955	0.0206	66

**Table 3:** Gene-set enrichment analysis (GSEA) of hub genes.

	Name	Size	ES	NES	NOM *P* val	FDR
S100A6	KEGG_HOMOLOGOUS_RECOMBINATION	26	0.62996	1.781231	0.002028	0.13548
	KEGG_PENTOSE_AND_GLUCURONATE_INTERCONVERSIONS	28	0.651928	1.652798	0.02924	0.139645
	KEGG_ASCORBATE_AND_ALDARATE_METABOLISM	25	0.658601	1.573314	0.042424	0.148149

S100A10	KEGG_DNA_REPLICATION	36	0.60398	1.67332	0.005906	0.073644
	KEGG_PENTOSE_PHOSPHATE_PATHWAY	27	0.617573	2.127046	0	0.006019
	KEGG_HOMOLOGOUS_RECOMBINATION	26	0.61573	1.753158	0	0.060837

S100A16	KEGG_PENTOSE_AND_GLUCURONATE_INTERCONVERSIONS	28	0.637038	1.628691	0.044922	0.102498
	KEGG_HOMOLOGOUS_RECOMBINATION	26	0.700146	1.999763	0	0.014572
	KEGG_DNA_REPLICATION	36	0.679325	1.905039	0	0.023962
	KEGG_MISMATCH_REPAIR	23	0.660125	1.851964	0	0.0311

SDC1	KEGG_P53_SIGNALING_PATHWAY	66	0.54107	2.013819	0	0.071246

## Data Availability

The data that support the findings of this study are openly available in Gene Expression Omnibus (GEO) database at http://www.ncbi.nlm.nih.gov/geo/, and The Cancer Genome Atlas (TCGA) database at https://genomecancer.ucsc.edu/.

## Supplementary Materials

Supplementary Materials Figure S1: (a) sample clustering to detect outliers. (b) Sample dendrogram and trait heatmap. The color intensity was proportional to disease status. Figure S2: determination of soft-thresholding power in the weighted gene coexpression network analysis (WGCNA). (a) Analysis of the scale-free fit index for various soft-thresholding powers (*β*). (b) Analysis of the mean connectivity for various soft-thresholding powers. (c) Histogram of connectivity distribution when *β* = 9. (d) Checking the scale free topology when *β* = 9. Figure S3: interaction relationship analysis of coexpression genes and construction of a classical MDS plot. (a) Different colors of horizontal axis and vertical axis represent different modules. The brightness of yellow in the middle represents the degree of connectivity of different modules. There was no significant difference in interactions among different modules, indicating a high-scale independence degree among these modules. (b) Classical MDS plot whose input is the TOM dissimilarity. Each dot (gene) is colored by the module assignment. Table S1: differentially expressed immune-related genes between normal samples and PAAD samples (based on MMD and TCGA). Table S2: differentially expressed genes (DEGs) between normal samples and PAAD samples (based on MMD). Table S3: risk score of each PAAD sample (TCGA-PAAD). Table S4: risk score of each PAAD sample (MMSD). Table S5: risk score of each PAAD sample (IMvigor210). Table S6: risk score of each PAAD sample (GSE78220).
