# Supplementary material for: A Novel Immune-Related Prognostic Signature Predicting Survival in Patients with Pancreatic Adenocarcinoma
Source: J Oncol. 2022 Mar 18;2022:8909631. doi: 10.1155/2022/8909631 (PMC8956421; doi:10.1155/2022/8909631)
Supplement: Supplementary Materials — Figure S1: (a) sample clustering to detect outliers. (b) Sample dendrogram and trait heatmap. The color intensity was proportional to disease status. Figure S2: determination of soft-thresholding power in the weighted gene coexpression network analysis (WGCNA). (a) Analysis of the scale-free fit index for various soft-thresholding powers (β). (b) Analysis of the mean connectivity for various soft-thresholding powers. (c) Histogram of connectivity distribution when β = 9. (d) Checking the scale free topology when β = 9. Figure S3: interaction relationship analysis of coexpression genes and construction of a classical MDS plot. (a) Different colors of horizontal axis and vertical axis represent different modules. The brightness of yellow in the middle represents the degree of connectivity of different modules. There was no significant difference in interactions among different modules, indicating a high-scale independence degree among these modules. (b) Classical MDS plot whose input is the TOM dissimilarity. Each dot (gene) is colored by the module assignment. Table S1: differentially expressed immune-related genes between normal samples and PAAD samples (based on MMD and TCGA). Table S2: differentially expressed genes (DEGs) between normal samples and PAAD samples (based on MMD). Table S3: risk score of each PAAD sample (TCGA-PAAD). Table S4: risk score of each PAAD sample (MMSD). Table S5: risk score of each PAAD sample (IMvigor210). Table S6: risk score of each PAAD sample (GSE78220). [file 8909631.f1.docx]

**Supplementary materials:**

Figure S1: (A) Sample clustering to detect outliers. (B) Sample dendrogram and trait heatmap. The color intensity was proportional to disease status.


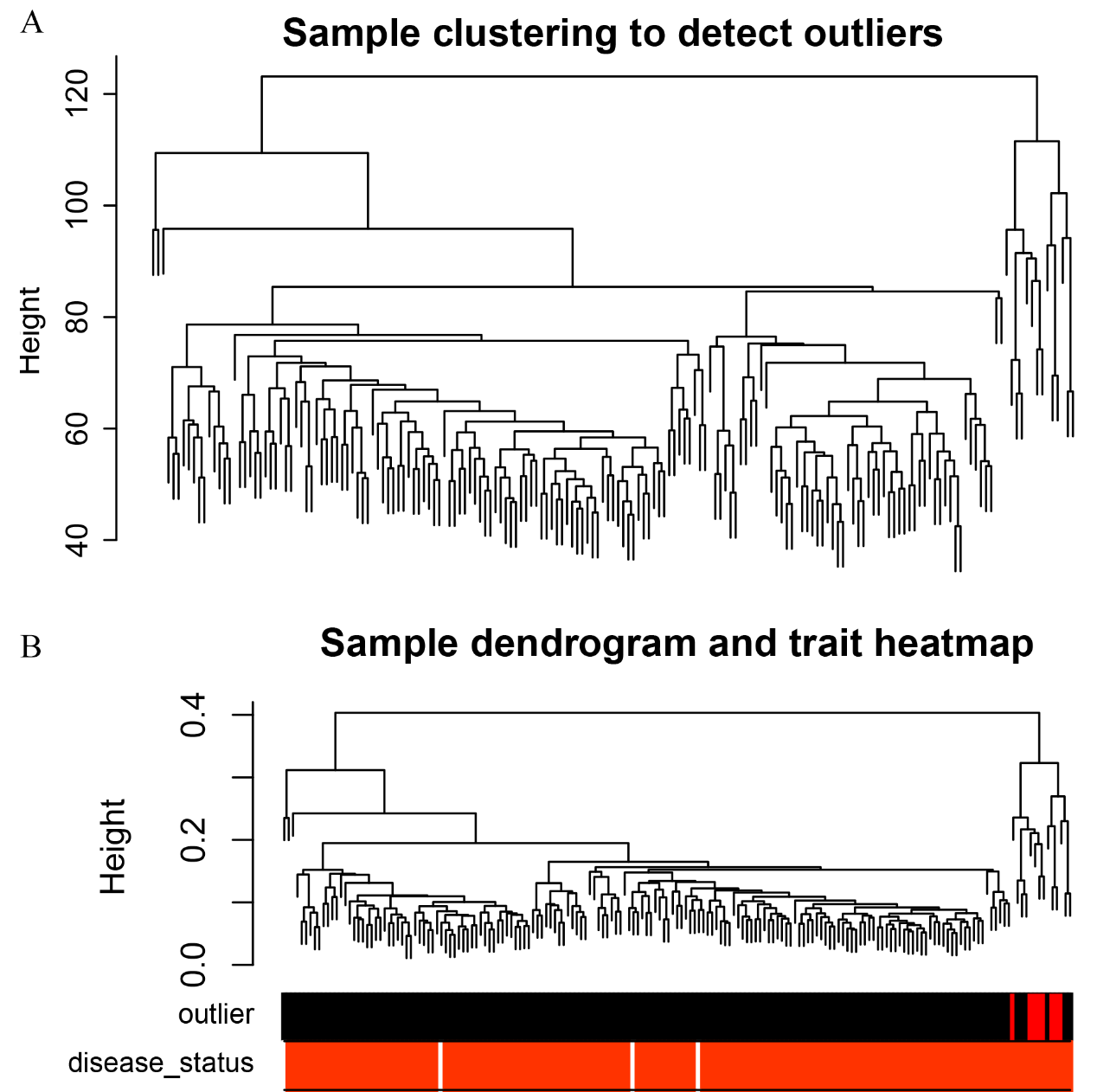


Figure S2: Determination of soft-thresholding power in the weighted gene co-expression network analysis (WGCNA). (A) Analysis of the scale-free fit index for various soft-thresholding powers (β). (B) Analysis of the mean connectivity for various soft-thresholding powers. (C) Histogram of connectivity distribution when β = 9. (D) Checking the scale free topology when β = 9.


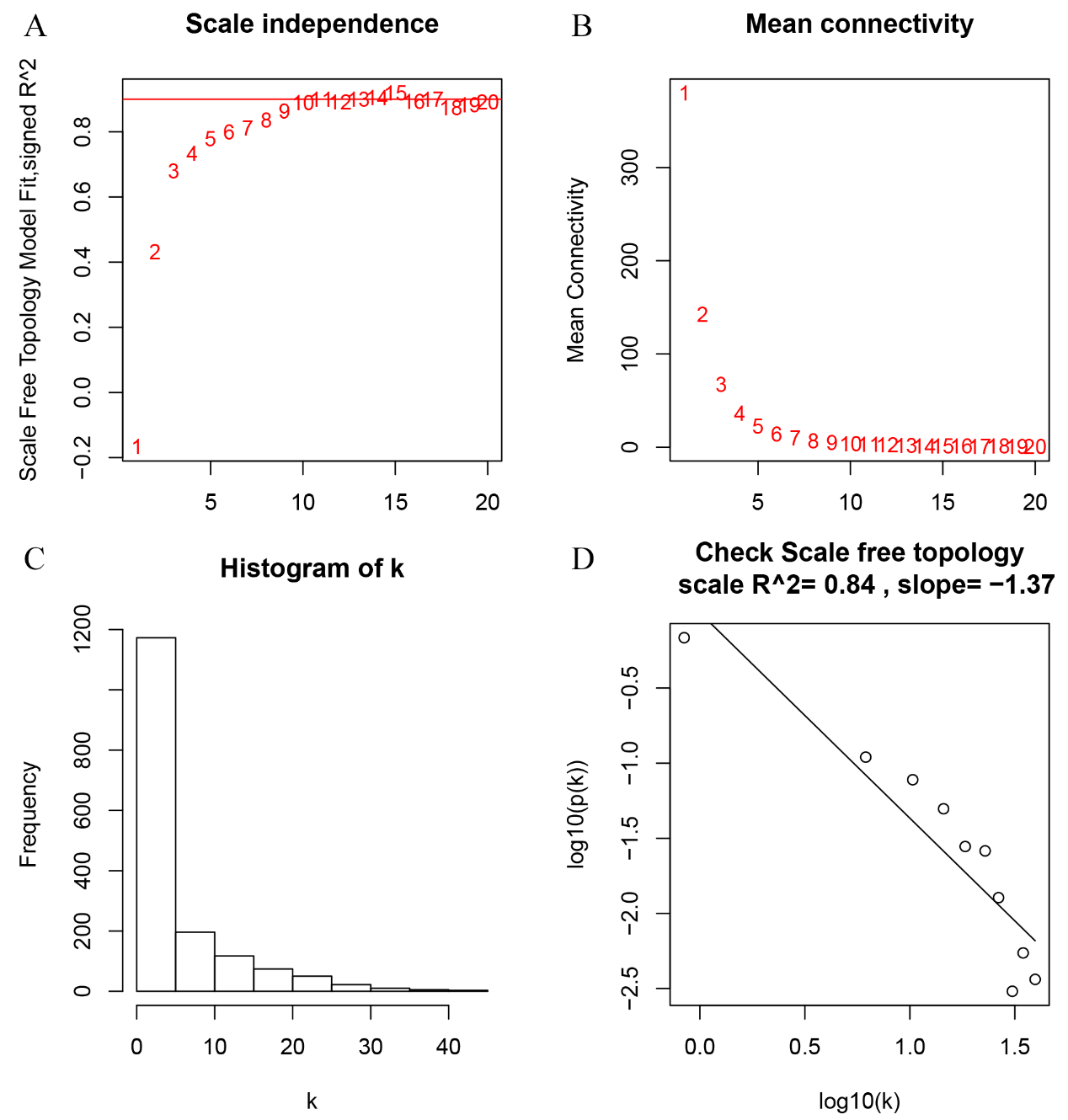


Figure S3: Interaction relationship analysis of co-expression genes and construction of a classical MDS plot. (A) Different colors of horizontal axis and vertical axis represent different modules. The brightness of yellow in the middle represents the degree of connectivity of different modules. There was no significant difference in interactions among different modules, indicating a high-scale independence degree among these modules. (B) Classical MDS plot whose input is the TOM dissimilarity. Each dot (gene) is colored by the module assignment.

**
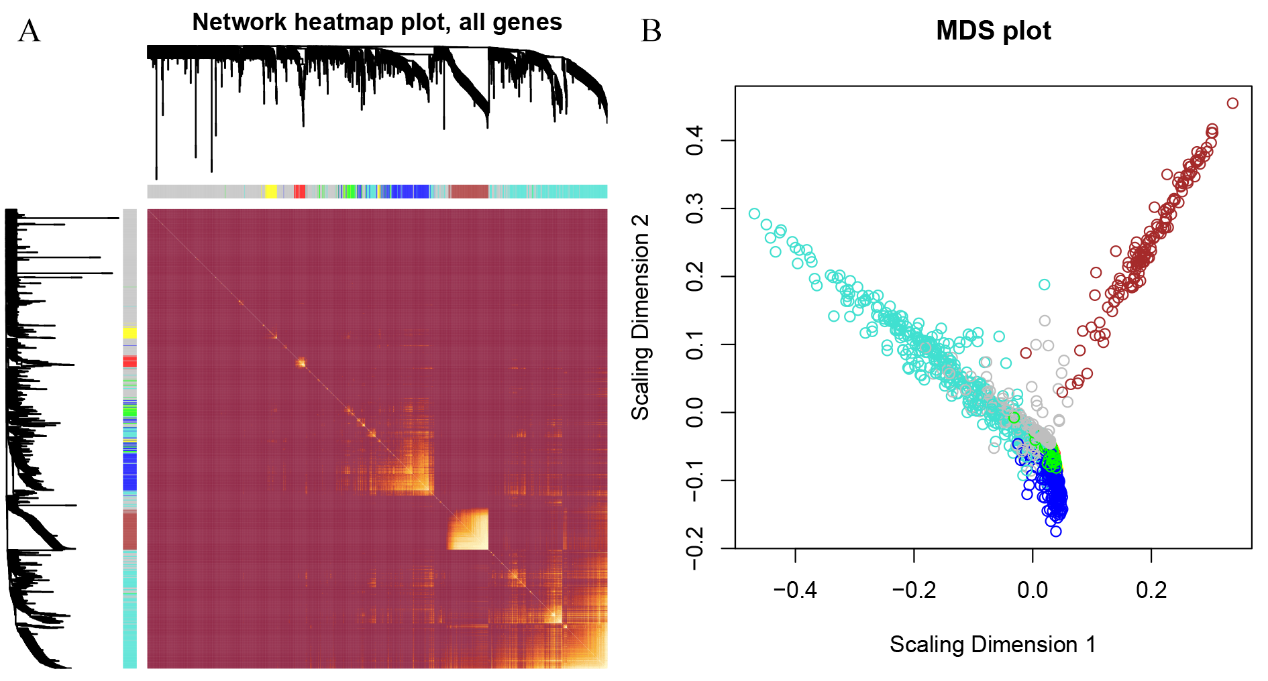
**

Table S1: Differentially expressed immune-related genes between normal samples and PAAD samples (based on MMD and TCGA).

| Gene symbol | MMD |  | TCGA |  |
| --- | --- | --- | --- | --- |
|  | logFC | adj.P.Val | logFC | adj.P.Val |
| AREG | 1.293775 | 1.16E-07 | 2.792383 | 0.136344 |
| CLDN4 | 1.208732 | 1.24E-14 | 2.015727 | 0.197594 |
| CST4 | 1.084928 | 5.38E-14 | 2.355923 | 0.264397 |
| CTSE | 2.881631 | 1.47E-19 | 3.327006 | 0.296242 |
| DMBT1 | 1.232904 | 3.16E-05 | 4.263011 | 0.197594 |
| EREG | 1.200051 | 7.24E-07 | 3.885215 | 0.156039 |
| MET | 1.642453 | 5.45E-22 | 1.428403 | 0.339969 |
| MIA | 1.250189 | 1.73E-11 | 2.739896 | 0.300676 |
| MUC4 | 2.098828 | 3.57E-13 | 2.770191 | 0.268537 |
| MUC5AC | 1.349705 | 2.02E-07 | 6.227065 | 0.097292 |
| PI3 | 1.657046 | 1.55E-14 | 2.701636 | 0.311039 |
| PTGS2 | 1.288225 | 7.61E-06 | 2.012032 | 0.297698 |
| S100A10 | 1.33459 | 1.81E-21 | 1.242578 | 0.245936 |
| S100A14 | 1.21981 | 1.35E-11 | 2.360837 | 0.334313 |
| S100A16 | 1.192128 | 5.32E-21 | 1.391449 | 0.268537 |
| S100A6 | 1.995106 | 2.02E-23 | 1.821862 | 0.156039 |
| S100P | 3.826254 | 2.81E-26 | 5.729119 | 0.027322 |
| SDC1 | 1.469016 | 5.32E-21 | 1.912016 | 0.156039 |
| SLPI | 2.347133 | 1.07E-26 | 2.539486 | 0.156039 |
| TNFRSF21 | 1.464369 | 2.45E-18 | 1.004369 | 0.339969 |
| ULBP2 | 1.161448 | 5.55E-14 | 1.459191 | 0.341679 |
| WFDC2 | 1.356818 | 5.11E-18 | 1.754512 | 0.311039 |

Table S2: Table S2: Differentially expressed genes (DEGs) between normal samples and PAAD samples (based on MMD).

| DEGs | logFC | AveExpr | t | P.Value | adj.P.Val | B |
| --- | --- | --- | --- | --- | --- | --- |
| LAMC2 | 2.592258 | 8.080519 | 13.80133 | 5.75E-30 | 8.13E-26 | 57.37527 |
| SLPI | 2.347133 | 9.081723 | 13.77377 | 6.91E-30 | 8.13E-26 | 57.19358 |
| S100P | 3.826254 | 9.157541 | 13.52313 | 3.72E-29 | 2.92E-25 | 55.54099 |
| ITGA2 | 2.300414 | 8.081181 | 13.23742 | 2.54E-28 | 1.35E-24 | 53.65597 |
| PHLDA2 | 2.512735 | 7.766305 | 13.21883 | 2.88E-28 | 1.35E-24 | 53.5333 |
| GPRC5A | 2.73979 | 8.769418 | 13.15612 | 4.39E-28 | 1.36E-24 | 53.11947 |
| KRT19 | 3.487771 | 9.355476 | 13.14333 | 4.78E-28 | 1.36E-24 | 53.03511 |
| AHNAK2 | 2.329356 | 7.320763 | 13.13159 | 5.18E-28 | 1.36E-24 | 52.95764 |
| GJB2 | 3.100292 | 6.985824 | 13.13074 | 5.21E-28 | 1.36E-24 | 52.95204 |
| ANXA2P2 | 1.046817 | 10.25582 | 12.87077 | 2.99E-27 | 7.02E-24 | 51.23663 |
| SFN | 2.758138 | 8.84639 | 12.7644 | 6.10E-27 | 1.31E-23 | 50.53495 |
| ANXA2 | 1.194805 | 12.22021 | 12.7174 | 8.37E-27 | 1.64E-23 | 50.22494 |
| ANO1 | 2.32789 | 6.966649 | 12.56914 | 2.27E-26 | 4.10E-23 | 49.24729 |
| TRIM29 | 2.367604 | 7.660882 | 12.49678 | 3.68E-26 | 5.96E-23 | 48.77033 |
| S100A6 | 1.995106 | 11.27079 | 12.48358 | 4.02E-26 | 5.96E-23 | 48.68334 |
| FGD6 | 1.262678 | 6.430235 | 12.48035 | 4.11E-26 | 5.96E-23 | 48.66206 |
| LAMB3 | 1.968276 | 8.133843 | 12.47327 | 4.31E-26 | 5.96E-23 | 48.61539 |
| SLC6A14 | 3.360204 | 5.884798 | 12.38433 | 7.83E-26 | 1.01E-22 | 48.02941 |
| INHBA | 3.40467 | 8.84352 | 12.37841 | 8.15E-26 | 1.01E-22 | 47.99047 |
| MYOF | 1.716186 | 9.557141 | 12.27094 | 1.67E-25 | 1.97E-22 | 47.28275 |
| S100A11 | 1.586134 | 10.33255 | 12.21198 | 2.49E-25 | 2.78E-22 | 46.89463 |
| ASAP2 | 1.18924 | 8.477623 | 12.19662 | 2.76E-25 | 2.95E-22 | 46.79357 |
| MBOAT2 | 1.383212 | 7.009285 | 12.12859 | 4.35E-25 | 4.44E-22 | 46.34602 |
| KRT7 | 2.590533 | 7.925674 | 12.06086 | 6.84E-25 | 6.70E-22 | 45.90061 |
| S100A11P1 /// S100A11P1 | 1.271022 | 9.583861 | 12.02367 | 8.78E-25 | 8.26E-22 | 45.65617 |
| NHS | 1.615128 | 6.297168 | 12.01563 | 9.26E-25 | 8.32E-22 | 45.60333 |
| NQO1 | 2.337752 | 8.220363 | 12.01095 | 9.56E-25 | 8.32E-22 | 45.57258 |
| SERPINB5 | 2.769272 | 6.702561 | 11.97802 | 1.19E-24 | 1.00E-21 | 45.35617 |
| FN1 | 2.425717 | 11.34734 | 11.94038 | 1.53E-24 | 1.24E-21 | 45.10892 |
| TMPRSS4 | 2.319004 | 7.475765 | 11.91187 | 1.85E-24 | 1.41E-21 | 44.92167 |
| MET | 1.642453 | 8.87415 | 11.88783 | 2.18E-24 | 1.60E-21 | 44.76382 |
| SOX4 | 1.368466 | 8.188825 | 11.79601 | 4.02E-24 | 2.87E-21 | 44.16125 |
| COL10A1 | 3.489307 | 8.071262 | 11.74915 | 5.50E-24 | 3.80E-21 | 43.85396 |
| S100A10 | 1.33459 | 11.74032 | 11.68664 | 8.35E-24 | 5.45E-21 | 43.44417 |
| LOC102725271 /// NTM | 2.616218 | 7.76561 | 11.62036 | 1.30E-23 | 8.11E-21 | 43.01002 |
| CAPG | 1.572365 | 8.392336 | 11.61901 | 1.31E-23 | 8.11E-21 | 43.00116 |
| MSLN | 2.313995 | 6.292607 | 11.58379 | 1.66E-23 | 9.99E-21 | 42.77062 |
| IGFBP3 | 2.358914 | 10.22616 | 11.56128 | 1.93E-23 | 1.13E-20 | 42.6233 |
| C15orf48 | 2.206278 | 8.700389 | 11.55097 | 2.06E-23 | 1.18E-20 | 42.55579 |
| S100A16 | 1.192128 | 9.919737 | 11.50551 | 2.79E-23 | 1.56E-20 | 42.25842 |
| COL11A1 | 3.503552 | 7.76744 | 11.49272 | 3.04E-23 | 1.64E-20 | 42.17478 |
| SDC1 | 1.469016 | 8.057839 | 11.48514 | 3.20E-23 | 1.64E-20 | 42.12522 |
| BIK | 1.53125 | 7.550213 | 11.48502 | 3.20E-23 | 1.64E-20 | 42.12447 |
| HOXB3 | 1.687841 | 7.895942 | 11.48267 | 3.25E-23 | 1.64E-20 | 42.1091 |
| HOXB7 | 1.434779 | 7.035488 | 11.48074 | 3.29E-23 | 1.64E-20 | 42.09648 |
| SULF1 | 2.92148 | 9.036567 | 11.47809 | 3.35E-23 | 1.64E-20 | 42.07912 |
| SDR16C5 | 2.694274 | 6.09419 | 11.46957 | 3.55E-23 | 1.70E-20 | 42.02345 |
| PMEPA1 | 1.57441 | 9.472644 | 11.45582 | 3.89E-23 | 1.83E-20 | 41.93355 |
| IFI27 | 2.106689 | 9.386835 | 11.44776 | 4.10E-23 | 1.89E-20 | 41.88088 |
| LAMA3 | 2.173751 | 7.901964 | 11.44016 | 4.31E-23 | 1.95E-20 | 41.83121 |
| ECT2 | 2.013907 | 6.409817 | 11.40056 | 5.61E-23 | 2.49E-20 | 41.57251 |
| C19orf33 | 2.816257 | 8.293025 | 11.39687 | 5.75E-23 | 2.50E-20 | 41.54843 |
| DCBLD1 | 1.279213 | 7.237207 | 11.39227 | 5.93E-23 | 2.54E-20 | 41.51837 |
| GJB3 | 1.10289 | 6.872031 | 11.34739 | 7.99E-23 | 3.36E-20 | 41.22534 |
| LY6E | 1.350114 | 7.878835 | 11.33264 | 8.81E-23 | 3.61E-20 | 41.1291 |
| NFE2L3 | 1.421288 | 5.861751 | 11.331 | 8.91E-23 | 3.61E-20 | 41.11843 |
| ITGB5 | 1.031478 | 9.523025 | 11.24722 | 1.55E-22 | 6.20E-20 | 40.57199 |
| KLK10 | 2.310607 | 7.089533 | 11.23493 | 1.69E-22 | 6.61E-20 | 40.49189 |
| PGM2L1 | 1.291484 | 7.610822 | 11.21838 | 1.88E-22 | 7.26E-20 | 40.38403 |
| CTHRC1 | 2.882794 | 10.12316 | 11.20683 | 2.03E-22 | 7.71E-20 | 40.30883 |
| FHL2 | 1.648119 | 9.281249 | 11.20244 | 2.09E-22 | 7.81E-20 | 40.28021 |
| PKM | 1.251514 | 9.554653 | 11.16943 | 2.60E-22 | 9.57E-20 | 40.06524 |
| SHISA2 | 1.995705 | 6.200017 | 11.13236 | 3.33E-22 | 1.20E-19 | 39.82392 |
| ITGB6 /// LOC100505984 | 2.643295 | 8.390162 | 11.11879 | 3.64E-22 | 1.28E-19 | 39.73569 |
| TFAP2A | 1.675941 | 5.888956 | 11.09577 | 4.24E-22 | 1.47E-19 | 39.58595 |
| TMPRSS3 | 1.885009 | 6.066436 | 11.066 | 5.17E-22 | 1.76E-19 | 39.39237 |
| SGPP2 | 1.802616 | 6.722121 | 11.03997 | 6.14E-22 | 2.05E-19 | 39.22318 |
| NOX4 | 2.101055 | 6.248947 | 11.03851 | 6.20E-22 | 2.05E-19 | 39.21372 |
| TSPAN1 | 1.960957 | 8.320007 | 11.01911 | 7.05E-22 | 2.27E-19 | 39.08767 |
| HS3ST1 | 2.00174 | 7.187259 | 10.97651 | 9.34E-22 | 2.97E-19 | 38.81107 |
| MMP11 | 1.948352 | 8.598568 | 10.97334 | 9.54E-22 | 2.99E-19 | 38.79047 |
| RTP4 | 1.186671 | 7.107319 | 10.95244 | 1.09E-21 | 3.39E-19 | 38.65483 |
| COL1A2 | 2.562936 | 11.64646 | 10.94929 | 1.12E-21 | 3.39E-19 | 38.63443 |
| CTSE | 2.881631 | 9.772533 | 10.94835 | 1.12E-21 | 3.39E-19 | 38.62827 |
| ADAM9 | 1.46946 | 9.817812 | 10.92317 | 1.33E-21 | 3.95E-19 | 38.465 |
| HK2 | 1.580046 | 7.403031 | 10.91244 | 1.43E-21 | 4.15E-19 | 38.39536 |
| ADAMTS12 | 1.664463 | 7.356693 | 10.91223 | 1.43E-21 | 4.15E-19 | 38.39401 |
| TPBG | 1.118476 | 8.85167 | 10.90952 | 1.45E-21 | 4.17E-19 | 38.37643 |
| CXCL5 | 3.461492 | 7.625502 | 10.89614 | 1.59E-21 | 4.50E-19 | 38.28973 |
| CLIC1 | 1.076996 | 10.52244 | 10.88748 | 1.68E-21 | 4.71E-19 | 38.23361 |
| MIR6787 /// SLC16A3 | 2.061894 | 6.964399 | 10.88572 | 1.70E-21 | 4.71E-19 | 38.22214 |
| HOXA3 | 1.65796 | 6.27284 | 10.86703 | 1.92E-21 | 5.26E-19 | 38.10103 |
| KCNK1 | 1.754729 | 8.618793 | 10.84778 | 2.18E-21 | 5.89E-19 | 37.97635 |
| CSTB | 1.11852 | 10.85785 | 10.84637 | 2.21E-21 | 5.89E-19 | 37.96723 |
| MICAL2 | 1.182099 | 9.485476 | 10.81059 | 2.79E-21 | 7.30E-19 | 37.73554 |
| ARNTL2 | 1.42034 | 5.656958 | 10.76121 | 3.87E-21 | 9.88E-19 | 37.41612 |
| CEACAM6 | 3.490697 | 9.728306 | 10.71783 | 5.14E-21 | 1.30E-18 | 37.13573 |
| MTMR11 | 1.70934 | 7.638572 | 10.71559 | 5.22E-21 | 1.31E-18 | 37.12126 |
| FERMT1 | 1.702753 | 7.251307 | 10.71338 | 5.30E-21 | 1.31E-18 | 37.10698 |
| FXYD3 | 2.08503 | 9.042141 | 10.67656 | 6.75E-21 | 1.65E-18 | 36.86915 |
| LGALS3BP | 1.360882 | 9.48769 | 10.65572 | 7.74E-21 | 1.86E-18 | 36.73463 |
| PRKCI | 1.053246 | 7.832072 | 10.6323 | 9.02E-21 | 2.14E-18 | 36.58354 |
| COL5A1 | 2.216557 | 8.050517 | 10.62168 | 9.68E-21 | 2.28E-18 | 36.51506 |
| DKK1 | 2.293468 | 6.225956 | 10.59836 | 1.13E-20 | 2.60E-18 | 36.36469 |
| MXRA5 | 1.708429 | 9.760795 | 10.57915 | 1.28E-20 | 2.89E-18 | 36.2409 |
| CDH3 | 1.514706 | 7.366861 | 10.53244 | 1.74E-20 | 3.89E-18 | 35.94007 |
| SLC6A6 | 1.751425 | 8.713916 | 10.50955 | 2.02E-20 | 4.48E-18 | 35.79279 |
| AMIGO2 | 1.504023 | 8.601683 | 10.50658 | 2.06E-20 | 4.53E-18 | 35.77368 |
| SMAGP | 1.114909 | 7.618854 | 10.47272 | 2.57E-20 | 5.55E-18 | 35.5559 |
| IL1RN | 1.952859 | 7.332614 | 10.47261 | 2.57E-20 | 5.55E-18 | 35.55521 |
| TNFRSF21 | 1.464369 | 7.342898 | 10.46509 | 2.70E-20 | 5.70E-18 | 35.50684 |
| ISG15 | 1.604859 | 8.674087 | 10.46434 | 2.72E-20 | 5.70E-18 | 35.50202 |
| CST1 | 2.596009 | 6.461904 | 10.4617 | 2.76E-20 | 5.75E-18 | 35.48504 |
| NMU | 2.018777 | 5.90002 | 10.45914 | 2.81E-20 | 5.80E-18 | 35.46862 |
| LGALS3 | 1.228185 | 11.81188 | 10.44003 | 3.18E-20 | 6.51E-18 | 35.34585 |
| MPZL2 | 1.347915 | 7.972326 | 10.43832 | 3.22E-20 | 6.53E-18 | 35.33481 |
| TMC5 | 2.644162 | 7.869361 | 10.42605 | 3.49E-20 | 7.01E-18 | 35.25603 |
| EPPK1 | 1.855417 | 7.203993 | 10.40761 | 3.94E-20 | 7.81E-18 | 35.1376 |
| FZD2 | 1.006159 | 6.111278 | 10.40688 | 3.95E-20 | 7.81E-18 | 35.13296 |
| CD9 | 1.188766 | 10.32748 | 10.3928 | 4.34E-20 | 8.43E-18 | 35.04255 |
| THBS2 | 2.341766 | 9.86376 | 10.38358 | 4.60E-20 | 8.88E-18 | 34.98342 |
| KIF26B | 1.994692 | 6.540643 | 10.35416 | 5.58E-20 | 1.07E-17 | 34.79474 |
| MMP28 | 1.633349 | 5.719014 | 10.34179 | 6.05E-20 | 1.14E-17 | 34.71539 |
| WFDC2 | 1.356818 | 7.60525 | 10.34118 | 6.07E-20 | 1.14E-17 | 34.71149 |
| POSTN | 3.256346 | 10.20241 | 10.33885 | 6.17E-20 | 1.14E-17 | 34.69656 |
| FAT1 | 1.176434 | 9.048629 | 10.33864 | 6.18E-20 | 1.14E-17 | 34.69523 |
| CKLF | 1.174245 | 7.548728 | 10.33695 | 6.24E-20 | 1.15E-17 | 34.68436 |
| APOL1 | 1.000782 | 7.938422 | 10.32827 | 6.61E-20 | 1.20E-17 | 34.62875 |
| MLPH | 1.642537 | 8.636443 | 10.31494 | 7.21E-20 | 1.29E-17 | 34.54339 |
| COL5A2 | 2.353535 | 8.969248 | 10.30141 | 7.87E-20 | 1.40E-17 | 34.45668 |
| ITGB4 | 1.295244 | 7.934785 | 10.29272 | 8.33E-20 | 1.47E-17 | 34.40108 |
| OSBPL3 | 1.410276 | 6.662067 | 10.29079 | 8.44E-20 | 1.47E-17 | 34.38868 |
| SLC2A1 | 1.531727 | 7.755019 | 10.29034 | 8.46E-20 | 1.47E-17 | 34.38585 |
| HN1 | 1.445121 | 8.14253 | 10.28103 | 8.99E-20 | 1.56E-17 | 34.3262 |
| ITGA3 | 1.268223 | 7.124415 | 10.26444 | 1.00E-19 | 1.72E-17 | 34.22006 |
| CBFA2T3 | -1.02255 | 5.690394 | -10.23 | 1.25E-19 | 2.14E-17 | 33.99949 |
| COL1A1 | 2.611083 | 8.106293 | 10.21431 | 1.39E-19 | 2.35E-17 | 33.89953 |
| ANLN | 1.968664 | 5.383459 | 10.21272 | 1.40E-19 | 2.36E-17 | 33.88932 |
| STEAP1 | 1.582357 | 7.767837 | 10.21117 | 1.42E-19 | 2.36E-17 | 33.87944 |
| PFKP | 1.252397 | 8.475962 | 10.20082 | 1.52E-19 | 2.51E-17 | 33.81332 |
| TMSB10 | 1.060789 | 12.6972 | 10.17618 | 1.78E-19 | 2.93E-17 | 33.65595 |
| NREP | 1.466896 | 8.840288 | 10.15942 | 1.99E-19 | 3.24E-17 | 33.54897 |
| CAPN8 | 2.341827 | 6.704301 | 10.15849 | 2.00E-19 | 3.24E-17 | 33.54302 |
| COL12A1 | 2.17989 | 6.839235 | 10.13546 | 2.32E-19 | 3.74E-17 | 33.39614 |
| LOC102724257 /// TMC5 | 2.664045 | 8.799998 | 10.12848 | 2.43E-19 | 3.86E-17 | 33.35162 |
| MGLL | 1.491815 | 8.461683 | 10.08467 | 3.23E-19 | 5.06E-17 | 33.07243 |
| CEACAM5 | 3.594974 | 8.228688 | 10.07389 | 3.46E-19 | 5.39E-17 | 33.00375 |
| EFNB2 | 1.634261 | 8.572028 | 10.05189 | 3.99E-19 | 6.17E-17 | 32.8637 |
| MARCKSL1 | 1.229743 | 9.551936 | 10.04235 | 4.24E-19 | 6.52E-17 | 32.803 |
| JUP /// KRT17 | 2.206214 | 6.74567 | 10.00244 | 5.50E-19 | 8.34E-17 | 32.54923 |
| RHBDL2 | 1.332009 | 4.533148 | 9.998679 | 5.63E-19 | 8.49E-17 | 32.52533 |
| CD55 | 1.743939 | 9.310128 | 9.97644 | 6.50E-19 | 9.74E-17 | 32.38406 |
| PPAPDC1A | 1.799061 | 6.176513 | 9.938048 | 8.34E-19 | 1.23E-16 | 32.14036 |
| COL8A1 | 2.766896 | 8.395573 | 9.932346 | 8.65E-19 | 1.26E-16 | 32.10419 |
| ITPR3 | 1.034309 | 8.704976 | 9.92533 | 9.05E-19 | 1.31E-16 | 32.05969 |
| KCNN4 | 1.284398 | 6.499379 | 9.92308 | 9.19E-19 | 1.31E-16 | 32.04542 |
| ACVR1 | 1.008267 | 8.463172 | 9.914906 | 9.68E-19 | 1.37E-16 | 31.99359 |
| ANTXR1 | 1.818966 | 9.663206 | 9.910687 | 9.95E-19 | 1.40E-16 | 31.96685 |
| MYRIP | -1.35569 | 5.1655 | -9.90098 | 1.06E-18 | 1.48E-16 | 31.90532 |
| C1GALT1 | 1.285807 | 6.889753 | 9.896466 | 1.09E-18 | 1.52E-16 | 31.87672 |
| LINC00511 /// LINC00673 | 1.302313 | 6.648682 | 9.891588 | 1.13E-18 | 1.56E-16 | 31.84581 |
| PTK6 | 1.570364 | 6.298936 | 9.884358 | 1.18E-18 | 1.62E-16 | 31.80001 |
| FOXL1 | 1.201639 | 5.658094 | 9.88036 | 1.21E-18 | 1.65E-16 | 31.77468 |
| IRS1 | 1.012417 | 5.692348 | 9.868489 | 1.31E-18 | 1.78E-16 | 31.69951 |
| FAM19A5 | 1.565111 | 5.453127 | 9.858876 | 1.39E-18 | 1.88E-16 | 31.63865 |
| AGR2 | 2.705781 | 9.905512 | 9.842362 | 1.55E-18 | 2.08E-16 | 31.53414 |
| EDNRA | 2.122636 | 6.786556 | 9.836157 | 1.61E-18 | 2.15E-16 | 31.49489 |
| CLDN23 | 1.777851 | 6.126294 | 9.83503 | 1.62E-18 | 2.15E-16 | 31.48776 |
| FOXQ1 | 1.930183 | 9.055042 | 9.825181 | 1.73E-18 | 2.27E-16 | 31.42547 |
| XAF1 | 1.496434 | 8.491567 | 9.825155 | 1.73E-18 | 2.27E-16 | 31.42531 |
| LCN2 | 2.610678 | 9.622054 | 9.811743 | 1.88E-18 | 2.45E-16 | 31.34051 |
| KIAA1217 | 1.014194 | 8.110598 | 9.799913 | 2.03E-18 | 2.63E-16 | 31.26575 |
| OSBPL10 | 1.591296 | 7.397156 | 9.7922 | 2.14E-18 | 2.73E-16 | 31.21702 |
| EFNA5 | 1.363784 | 6.420452 | 9.774513 | 2.39E-18 | 3.04E-16 | 31.10531 |
| LOC100506403 /// LOC101928269 /// RUNX1 | 1.353738 | 8.665151 | 9.772757 | 2.42E-18 | 3.06E-16 | 31.09422 |
| STYK1 | 1.624445 | 4.683213 | 9.76284 | 2.58E-18 | 3.23E-16 | 31.03162 |
| VCAN | 2.397907 | 9.01176 | 9.747324 | 2.85E-18 | 3.54E-16 | 30.93371 |
| ABHD17C | 1.526004 | 7.685285 | 9.74687 | 2.86E-18 | 3.54E-16 | 30.93084 |
| SAMD9 | 1.49379 | 7.318705 | 9.74552 | 2.88E-18 | 3.55E-16 | 30.92232 |
| OAS3 | 1.120485 | 7.360932 | 9.744067 | 2.91E-18 | 3.57E-16 | 30.91316 |
| EPS8L1 | 1.255336 | 7.452301 | 9.741735 | 2.96E-18 | 3.60E-16 | 30.89845 |
| CD109 | 1.649854 | 6.934125 | 9.740905 | 2.97E-18 | 3.60E-16 | 30.89321 |
| RRAS | 1.008132 | 7.822163 | 9.737469 | 3.04E-18 | 3.65E-16 | 30.87154 |
| NET1 | 1.094364 | 8.950097 | 9.735668 | 3.07E-18 | 3.67E-16 | 30.86018 |
| SFTA2 | 1.914459 | 6.126301 | 9.73358 | 3.11E-18 | 3.67E-16 | 30.84701 |
| ETV1 | 1.565699 | 7.241786 | 9.707521 | 3.68E-18 | 4.25E-16 | 30.68274 |
| LOXL2 | 1.43638 | 7.99352 | 9.681224 | 4.36E-18 | 4.93E-16 | 30.5171 |
| TM4SF1 | 1.42857 | 9.810249 | 9.673603 | 4.58E-18 | 5.15E-16 | 30.46913 |
| TMEM158 | 1.552182 | 6.755544 | 9.659812 | 5.00E-18 | 5.57E-16 | 30.38234 |
| SULF2 | 1.672077 | 8.69743 | 9.636514 | 5.81E-18 | 6.41E-16 | 30.23581 |
| FBXO32 | 1.593948 | 6.072466 | 9.629766 | 6.06E-18 | 6.66E-16 | 30.19339 |
| CTTNBP2NL | 1.03433 | 8.424572 | 9.62686 | 6.18E-18 | 6.76E-16 | 30.17512 |
| C1orf106 | 1.695195 | 8.688674 | 9.622044 | 6.37E-18 | 6.94E-16 | 30.14485 |
| RALA | 1.011256 | 7.958834 | 9.620509 | 6.43E-18 | 6.97E-16 | 30.1352 |
| ACTN1 | 1.017512 | 8.961618 | 9.610629 | 6.85E-18 | 7.33E-16 | 30.07313 |
| TACSTD2 | 2.234581 | 10.57157 | 9.61048 | 6.86E-18 | 7.33E-16 | 30.07219 |
| TIMP1 | 1.689446 | 11.72083 | 9.58858 | 7.89E-18 | 8.36E-16 | 29.93466 |
| TNFSF4 | 1.271512 | 5.908196 | 9.582059 | 8.23E-18 | 8.68E-16 | 29.89374 |
| KLF5 | 1.976513 | 7.356374 | 9.57936 | 8.37E-18 | 8.79E-16 | 29.8768 |
| GALNT5 | 1.474821 | 5.474468 | 9.56899 | 8.95E-18 | 9.35E-16 | 29.81173 |
| SYTL2 | 1.497238 | 8.477558 | 9.54296 | 1.06E-17 | 1.09E-15 | 29.6485 |
| ANXA3 | 1.444363 | 8.56014 | 9.540774 | 1.07E-17 | 1.11E-15 | 29.6348 |
| HOXC6 | 1.68912 | 6.685185 | 9.537046 | 1.10E-17 | 1.13E-15 | 29.61143 |
| SESTD1 | 1.003434 | 6.440289 | 9.529543 | 1.15E-17 | 1.17E-15 | 29.56442 |
| MINOS1-NBL1 /// NBL1 | 1.205812 | 9.179353 | 9.529219 | 1.15E-17 | 1.17E-15 | 29.56238 |
| GABRP | 2.873205 | 6.858399 | 9.501266 | 1.38E-17 | 1.39E-15 | 29.38734 |
| ECM1 | 1.189162 | 7.862102 | 9.494393 | 1.44E-17 | 1.44E-15 | 29.34432 |
| PON2 | 1.023459 | 8.414331 | 9.475918 | 1.62E-17 | 1.62E-15 | 29.22874 |
| LTBP1 | 1.121857 | 8.773561 | 9.464484 | 1.74E-17 | 1.72E-15 | 29.15724 |
| PLEK2 | 1.127749 | 7.761496 | 9.449652 | 1.92E-17 | 1.87E-15 | 29.06454 |
| KLF7 | 1.044195 | 6.443707 | 9.435974 | 2.09E-17 | 2.02E-15 | 28.97909 |
| TRIM59 | 1.208806 | 5.355951 | 9.431611 | 2.15E-17 | 2.07E-15 | 28.95185 |
| JUP | 1.100452 | 8.742277 | 9.431417 | 2.15E-17 | 2.07E-15 | 28.95063 |
| BLACAT1 | 1.279133 | 5.545344 | 9.424455 | 2.25E-17 | 2.14E-15 | 28.90717 |
| KYNU | 1.958489 | 7.087695 | 9.411123 | 2.45E-17 | 2.31E-15 | 28.82395 |
| SQLE | 1.27258 | 7.577106 | 9.380514 | 2.98E-17 | 2.79E-15 | 28.63305 |
| NPC1 | 1.146856 | 7.783142 | 9.356427 | 3.47E-17 | 3.19E-15 | 28.48297 |
| KRT23 | 2.001166 | 6.231898 | 9.345509 | 3.72E-17 | 3.37E-15 | 28.41498 |
| CKLF /// CKLF-CMTM1 | 1.198154 | 8.457982 | 9.344753 | 3.73E-17 | 3.38E-15 | 28.41028 |
| DPY19L1 | 1.147663 | 7.082707 | 9.343643 | 3.76E-17 | 3.39E-15 | 28.40337 |
| MST1R | 1.252931 | 6.737994 | 9.337563 | 3.91E-17 | 3.49E-15 | 28.36552 |
| AHR | 1.219214 | 9.230685 | 9.302441 | 4.88E-17 | 4.29E-15 | 28.14707 |
| PLD1 | 1.200838 | 5.463762 | 9.28365 | 5.50E-17 | 4.76E-15 | 28.03029 |
| NBL1 | 1.15861 | 8.747229 | 9.273107 | 5.88E-17 | 5.05E-15 | 27.96482 |
| AK4 /// LOC100507855 | 1.556699 | 7.279909 | 9.260255 | 6.38E-17 | 5.44E-15 | 27.88504 |
| LPCAT4 | 1.140692 | 7.391393 | 9.25199 | 6.72E-17 | 5.69E-15 | 27.83375 |
| RP11-391M1.4 | -1.06627 | 6.303369 | -9.24972 | 6.82E-17 | 5.75E-15 | 27.81963 |
| COL3A1 | 1.957978 | 8.525071 | 9.236386 | 7.42E-17 | 6.21E-15 | 27.73696 |
| SRPX2 | 1.526322 | 6.592157 | 9.228709 | 7.79E-17 | 6.49E-15 | 27.68936 |
| LOC100130872 /// SPON2 | 1.176291 | 8.617258 | 9.209072 | 8.82E-17 | 7.22E-15 | 27.56768 |
| SERPINH1 | 1.261309 | 8.491627 | 9.202574 | 9.19E-17 | 7.50E-15 | 27.52743 |
| ZNF703 | 1.005201 | 7.259398 | 9.171308 | 1.12E-16 | 9.01E-15 | 27.33391 |
| OASL | 1.1169 | 6.025223 | 9.16797 | 1.14E-16 | 9.13E-15 | 27.31326 |
| CD58 | 1.204294 | 8.311559 | 9.167564 | 1.15E-16 | 9.13E-15 | 27.31075 |
| SGIP1 | 1.02767 | 4.893454 | 9.165442 | 1.16E-16 | 9.23E-15 | 27.29763 |
| WISP1 | 2.328101 | 6.902055 | 9.157008 | 1.22E-16 | 9.70E-15 | 27.24548 |
| GPX8 | 1.438887 | 6.788018 | 9.132584 | 1.43E-16 | 1.12E-14 | 27.09455 |
| SPARC | 1.738335 | 11.67085 | 9.095381 | 1.81E-16 | 1.39E-14 | 26.86494 |
| KAL1 | 1.274949 | 6.635597 | 9.079549 | 1.99E-16 | 1.52E-14 | 26.76733 |
| LEF1 | 1.93297 | 7.320431 | 9.075435 | 2.05E-16 | 1.56E-14 | 26.74197 |
| RAI14 | 1.070313 | 8.421465 | 9.064842 | 2.19E-16 | 1.65E-14 | 26.6767 |
| C11orf80 | 1.265269 | 6.057569 | 9.061463 | 2.23E-16 | 1.68E-14 | 26.65589 |
| AP1S3 | 1.174266 | 4.621492 | 9.057555 | 2.29E-16 | 1.72E-14 | 26.63182 |
| CEMIP | 2.135806 | 6.829983 | 9.054185 | 2.34E-16 | 1.74E-14 | 26.61107 |
| CLDN4 | 1.208732 | 7.123422 | 9.024547 | 2.82E-16 | 2.03E-14 | 26.42868 |
| WNT5A | 1.245049 | 7.091405 | 9.009965 | 3.09E-16 | 2.21E-14 | 26.33903 |
| EXPH5 | 1.023721 | 5.091905 | 9.001547 | 3.25E-16 | 2.33E-14 | 26.28731 |
| CEACAM1 | 1.706157 | 7.324959 | 8.994862 | 3.39E-16 | 2.41E-14 | 26.24624 |
| PLAT | 1.819287 | 9.282111 | 8.992647 | 3.44E-16 | 2.43E-14 | 26.23263 |
| ELOVL6 | 1.384286 | 6.130424 | 8.986265 | 3.58E-16 | 2.51E-14 | 26.19344 |
| LMO7 | 1.240324 | 8.145767 | 8.98357 | 3.64E-16 | 2.54E-14 | 26.17689 |
| OCIAD2 | 1.028163 | 9.713315 | 8.979749 | 3.73E-16 | 2.58E-14 | 26.15343 |
| PI3 | 1.657046 | 6.400805 | 8.977749 | 3.78E-16 | 2.61E-14 | 26.14115 |
| RUNX2 | 1.926964 | 7.484176 | 8.957075 | 4.30E-16 | 2.93E-14 | 26.0143 |
| DIO2 | 1.345445 | 6.76771 | 8.948367 | 4.54E-16 | 3.08E-14 | 25.96091 |
| PRC1 | 1.100822 | 6.465291 | 8.946771 | 4.59E-16 | 3.10E-14 | 25.95112 |
| GPX2 | 2.137466 | 8.638432 | 8.942342 | 4.71E-16 | 3.18E-14 | 25.92398 |
| CAMK2N1 | 1.378486 | 9.159903 | 8.940947 | 4.76E-16 | 3.20E-14 | 25.91542 |
| ANKRD22 | 1.544283 | 7.126818 | 8.920516 | 5.40E-16 | 3.56E-14 | 25.79026 |
| NPR3 | 1.606926 | 5.839331 | 8.916988 | 5.52E-16 | 3.63E-14 | 25.76866 |
| UNC5B | 1.022662 | 6.814635 | 8.916459 | 5.54E-16 | 3.63E-14 | 25.76542 |
| RAB31 | 1.683897 | 9.189834 | 8.909235 | 5.80E-16 | 3.78E-14 | 25.7212 |
| ZC3HAV1L | 1.009552 | 7.92813 | 8.903398 | 6.01E-16 | 3.89E-14 | 25.68547 |
| RSAD2 | 1.498413 | 6.555594 | 8.903126 | 6.02E-16 | 3.89E-14 | 25.68381 |
| FAP | 1.89624 | 7.919502 | 8.900953 | 6.11E-16 | 3.92E-14 | 25.67051 |
| HOPX | 1.993768 | 8.966944 | 8.892987 | 6.42E-16 | 4.08E-14 | 25.62178 |
| ENO2 | 1.296914 | 6.434934 | 8.891804 | 6.46E-16 | 4.10E-14 | 25.61454 |
| EFNA1 | 1.05447 | 8.442512 | 8.888906 | 6.58E-16 | 4.16E-14 | 25.59682 |
| EDIL3 | 1.684495 | 9.230253 | 8.874062 | 7.22E-16 | 4.50E-14 | 25.50607 |
| MAL2 | 1.761433 | 9.196633 | 8.873585 | 7.24E-16 | 4.51E-14 | 25.50316 |
| LEMD1 | 1.503374 | 5.255307 | 8.866585 | 7.57E-16 | 4.67E-14 | 25.46038 |
| CRIP1 | 1.535226 | 8.27784 | 8.861909 | 7.79E-16 | 4.78E-14 | 25.43181 |
| COL16A1 | 1.032989 | 8.29533 | 8.844789 | 8.67E-16 | 5.25E-14 | 25.32728 |
| RACGAP1 | 1.223443 | 6.809678 | 8.834728 | 9.23E-16 | 5.56E-14 | 25.26588 |
| LOC100129406 | 1.054728 | 5.395799 | 8.83292 | 9.33E-16 | 5.61E-14 | 25.25485 |
| MEGF6 | 1.005166 | 6.706946 | 8.832374 | 9.36E-16 | 5.62E-14 | 25.25152 |
| SPAG1 | 1.004231 | 5.803481 | 8.8053 | 1.11E-15 | 6.45E-14 | 25.08645 |
| SLC16A10 | -2.0288 | 5.161737 | -8.80068 | 1.14E-15 | 6.62E-14 | 25.05832 |
| GCNT3 | 2.058903 | 8.131388 | 8.794068 | 1.19E-15 | 6.87E-14 | 25.01802 |
| STS | 1.041932 | 6.408001 | 8.79027 | 1.22E-15 | 7.01E-14 | 24.9949 |
| PCDH7 | 1.978538 | 7.239607 | 8.79001 | 1.22E-15 | 7.01E-14 | 24.99331 |
| SLC9B2 | 1.055001 | 6.705907 | 8.787056 | 1.24E-15 | 7.10E-14 | 24.97533 |
| P2RX1 | -1.38863 | 7.090102 | -8.78244 | 1.28E-15 | 7.28E-14 | 24.94724 |
| B4GALT5 | 1.029009 | 8.521882 | 8.779174 | 1.30E-15 | 7.39E-14 | 24.92734 |
| IQGAP3 | 1.115785 | 5.893825 | 8.773474 | 1.35E-15 | 7.60E-14 | 24.89265 |
| SLC4A11 | 1.215364 | 5.717593 | 8.771527 | 1.37E-15 | 7.67E-14 | 24.88081 |
| FXYD5 | 1.33675 | 8.931797 | 8.767773 | 1.40E-15 | 7.83E-14 | 24.85797 |
| NUAK1 | 1.180056 | 7.975074 | 8.761082 | 1.46E-15 | 8.13E-14 | 24.81728 |
| CST4 | 1.084928 | 7.366514 | 8.757597 | 1.49E-15 | 8.27E-14 | 24.79608 |
| SLC44A4 | 1.916285 | 7.443912 | 8.757574 | 1.49E-15 | 8.27E-14 | 24.79594 |
| MFAP2 | 1.241251 | 8.054499 | 8.750444 | 1.56E-15 | 8.58E-14 | 24.7526 |
| ULBP2 | 1.161448 | 5.014758 | 8.747547 | 1.59E-15 | 8.71E-14 | 24.73498 |
| DDX60 | 1.272287 | 7.779761 | 8.743271 | 1.63E-15 | 8.91E-14 | 24.709 |
| PLAUR | 1.292302 | 8.114264 | 8.742952 | 1.63E-15 | 8.91E-14 | 24.70706 |
| DKK3 | 1.260965 | 7.034728 | 8.731962 | 1.75E-15 | 9.49E-14 | 24.6403 |
| FAM46C | -1.28496 | 8.66308 | -8.72863 | 1.78E-15 | 9.66E-14 | 24.62009 |
| LPCAT2 | 1.087873 | 6.95452 | 8.720377 | 1.88E-15 | 1.01E-13 | 24.56996 |
| ANTXR2 | 1.345657 | 8.671429 | 8.712415 | 1.97E-15 | 1.06E-13 | 24.52163 |
| MMP7 | 2.286043 | 10.18182 | 8.70828 | 2.02E-15 | 1.08E-13 | 24.49654 |
| TOP2A | 1.864681 | 6.185694 | 8.706942 | 2.04E-15 | 1.09E-13 | 24.48842 |
| ECHDC3 | -1.00739 | 6.817238 | -8.70694 | 2.04E-15 | 1.09E-13 | 24.4884 |
| SLC16A4 | 1.479129 | 6.696541 | 8.70489 | 2.07E-15 | 1.10E-13 | 24.47598 |
| ADAMTS2 | 1.634717 | 8.010119 | 8.697364 | 2.16E-15 | 1.15E-13 | 24.43033 |
| PAIP2B | -1.55785 | 6.30635 | -8.69177 | 2.24E-15 | 1.18E-13 | 24.3964 |
| FSCN1 | 1.349339 | 5.881911 | 8.690274 | 2.26E-15 | 1.19E-13 | 24.38735 |
| IGF2BP3 | 1.846082 | 5.233983 | 8.679774 | 2.41E-15 | 1.26E-13 | 24.32371 |
| CDH11 | 2.068817 | 9.319709 | 8.678663 | 2.43E-15 | 1.26E-13 | 24.31698 |
| IFI44 | 1.335828 | 7.909756 | 8.674284 | 2.50E-15 | 1.29E-13 | 24.29045 |
| MXRA8 | 1.057811 | 8.584592 | 8.665803 | 2.63E-15 | 1.35E-13 | 24.23909 |
| CCDC69 | -1.03869 | 6.865857 | -8.66195 | 2.69E-15 | 1.37E-13 | 24.21576 |
| CALD1 | 1.359695 | 7.030799 | 8.654253 | 2.83E-15 | 1.43E-13 | 24.16916 |
| NT5DC2 | 1.001995 | 6.552408 | 8.654043 | 2.83E-15 | 1.43E-13 | 24.16789 |
| S100A4 | 1.568481 | 10.05577 | 8.638127 | 3.12E-15 | 1.57E-13 | 24.07161 |
| SLC24A3 | 1.123079 | 6.837785 | 8.601214 | 3.92E-15 | 1.94E-13 | 23.84858 |
| PPL | 1.038086 | 8.189048 | 8.596901 | 4.03E-15 | 1.99E-13 | 23.82254 |
| EPHX1 | -1.11855 | 8.794291 | -8.57556 | 4.59E-15 | 2.25E-13 | 23.69382 |
| COL6A3 | 1.503661 | 11.18449 | 8.571918 | 4.69E-15 | 2.28E-13 | 23.67184 |
| WNT2 | 1.081776 | 6.685067 | 8.564448 | 4.92E-15 | 2.37E-13 | 23.62682 |
| TCN1 | 2.521818 | 8.801341 | 8.561907 | 4.99E-15 | 2.40E-13 | 23.6115 |
| OAS1 | 1.29127 | 6.671692 | 8.548247 | 5.43E-15 | 2.56E-13 | 23.52922 |
| CLDN18 | 3.237182 | 5.806794 | 8.54796 | 5.44E-15 | 2.56E-13 | 23.52749 |
| CENPF | 1.140138 | 6.79047 | 8.547525 | 5.45E-15 | 2.57E-13 | 23.52487 |
| MDK | 1.098641 | 7.807204 | 8.540351 | 5.70E-15 | 2.67E-13 | 23.48168 |
| FNDC1 | 1.906163 | 6.957067 | 8.534549 | 5.91E-15 | 2.75E-13 | 23.44676 |
| CDK1 | 1.436767 | 6.900442 | 8.533049 | 5.96E-15 | 2.77E-13 | 23.43774 |
| MIR612 /// NEAT1 | 1.029271 | 10.49015 | 8.506673 | 7.01E-15 | 3.22E-13 | 23.27913 |
| PLAU | 1.503558 | 7.014068 | 8.500725 | 7.27E-15 | 3.33E-13 | 23.24339 |
| HIST1H2BD | 1.262883 | 7.723556 | 8.49118 | 7.71E-15 | 3.50E-13 | 23.18605 |
| BGN | 1.515556 | 9.350133 | 8.473374 | 8.60E-15 | 3.87E-13 | 23.07918 |
| EPSTI1 | 1.328265 | 7.850111 | 8.472763 | 8.63E-15 | 3.87E-13 | 23.07551 |
| SPOCK1 | 1.341973 | 7.262907 | 8.47228 | 8.66E-15 | 3.88E-13 | 23.07262 |
| LOXL1 | 1.342421 | 7.697936 | 8.459495 | 9.36E-15 | 4.17E-13 | 22.99593 |
| TMEM173 | 1.062798 | 7.041453 | 8.449895 | 9.93E-15 | 4.39E-13 | 22.9384 |
| VDR | 1.251429 | 7.386509 | 8.449237 | 9.97E-15 | 4.40E-13 | 22.93445 |
| ARPC1B | 1.167073 | 9.431209 | 8.444597 | 1.03E-14 | 4.50E-13 | 22.90664 |
| ITGB1 | 1.157095 | 9.889442 | 8.437889 | 1.07E-14 | 4.66E-13 | 22.86647 |
| AEBP1 | 1.595678 | 9.416323 | 8.43256 | 1.10E-14 | 4.78E-13 | 22.83456 |
| IFT80 /// TRIM59 | 1.143374 | 6.157648 | 8.431841 | 1.11E-14 | 4.79E-13 | 22.83025 |
| FAM83H | 1.040091 | 6.961288 | 8.425399 | 1.15E-14 | 4.96E-13 | 22.79168 |
| MX1 | 1.270888 | 8.699962 | 8.422065 | 1.18E-14 | 5.05E-13 | 22.77173 |
| MELK | 1.240307 | 6.685172 | 8.418403 | 1.20E-14 | 5.15E-13 | 22.74982 |
| NUSAP1 | 1.336691 | 6.918953 | 8.412065 | 1.25E-14 | 5.32E-13 | 22.7119 |
| FKBP11 | -1.14574 | 8.96781 | -8.41079 | 1.26E-14 | 5.34E-13 | 22.7043 |
| BHLHE40 | 1.083204 | 10.16453 | 8.40256 | 1.33E-14 | 5.58E-13 | 22.65506 |
| SCEL | 1.613851 | 4.595636 | 8.400032 | 1.35E-14 | 5.65E-13 | 22.63995 |
| PALLD | 1.380933 | 10.16961 | 8.386732 | 1.46E-14 | 6.05E-13 | 22.56048 |
| CERS6 | 1.022687 | 5.750753 | 8.385999 | 1.47E-14 | 6.05E-13 | 22.5561 |
| CST2 | 1.228718 | 6.882274 | 8.385362 | 1.47E-14 | 6.05E-13 | 22.55229 |
| MUC4 | 2.098828 | 5.872219 | 8.383458 | 1.49E-14 | 6.10E-13 | 22.54092 |
| OLR1 | 1.875905 | 6.077737 | 8.374484 | 1.57E-14 | 6.37E-13 | 22.48733 |
| LINC00152 /// LOC101930489 /// MIR4435-1HG | 1.110542 | 8.796407 | 8.351726 | 1.81E-14 | 7.22E-13 | 22.35155 |
| TFF1 | 2.896796 | 9.02288 | 8.343327 | 1.90E-14 | 7.54E-13 | 22.30148 |
| BTG2 | -1.10943 | 9.758939 | -8.33987 | 1.94E-14 | 7.68E-13 | 22.28085 |
| OLFML2B | 1.647119 | 7.602086 | 8.337794 | 1.97E-14 | 7.75E-13 | 22.2685 |
| F5 | 1.572248 | 6.894154 | 8.33071 | 2.05E-14 | 8.08E-13 | 22.2263 |
| MTUS2 | -1.51865 | 5.019341 | -8.32713 | 2.10E-14 | 8.20E-13 | 22.20496 |
| ASPH | 1.057757 | 8.295418 | 8.317158 | 2.23E-14 | 8.66E-13 | 22.14561 |
| COL4A1 | 1.349383 | 8.758339 | 8.314385 | 2.27E-14 | 8.78E-13 | 22.12911 |
| STAT1 | 1.32183 | 7.736318 | 8.309336 | 2.34E-14 | 9.03E-13 | 22.09906 |
| DCBLD2 | 1.125141 | 7.323671 | 8.302121 | 2.44E-14 | 9.36E-13 | 22.05614 |
| TGM2 | 1.369647 | 8.304964 | 8.300555 | 2.47E-14 | 9.44E-13 | 22.04683 |
| TMEM200A | 1.666855 | 7.206601 | 8.290265 | 2.63E-14 | 1.00E-12 | 21.98565 |
| SLC39A10 | 1.145933 | 7.676595 | 8.287072 | 2.68E-14 | 1.01E-12 | 21.96667 |
| LIPH | 1.020263 | 5.595099 | 8.284754 | 2.72E-14 | 1.03E-12 | 21.9529 |
| CEP55 | 1.291146 | 5.304792 | 8.27746 | 2.84E-14 | 1.07E-12 | 21.90956 |
| NDC80 | 1.327546 | 5.923911 | 8.273199 | 2.91E-14 | 1.09E-12 | 21.88426 |
| COL4A2 | 1.271155 | 10.51552 | 8.271108 | 2.95E-14 | 1.10E-12 | 21.87184 |
| DGKH | 1.081617 | 5.669548 | 8.268216 | 3.00E-14 | 1.12E-12 | 21.85467 |
| SCD | 1.434945 | 8.209573 | 8.262377 | 3.11E-14 | 1.15E-12 | 21.82001 |
| PMAIP1 | 1.402404 | 6.676969 | 8.258852 | 3.18E-14 | 1.18E-12 | 21.79909 |
| TPRG1 | 1.257019 | 5.200071 | 8.25447 | 3.26E-14 | 1.20E-12 | 21.7731 |
| TGFBI | 1.286661 | 9.963106 | 8.253848 | 3.28E-14 | 1.20E-12 | 21.7694 |
| MUC20 | 1.412052 | 6.346102 | 8.251924 | 3.31E-14 | 1.21E-12 | 21.75799 |
| IGFBP5 | 1.612432 | 10.20199 | 8.243967 | 3.48E-14 | 1.27E-12 | 21.7108 |
| COMP | 2.134602 | 7.319519 | 8.240226 | 3.56E-14 | 1.29E-12 | 21.68862 |
| MSMO1 | 1.024729 | 8.672874 | 8.238297 | 3.60E-14 | 1.30E-12 | 21.67719 |
| SLC25A45 | -1.05767 | 3.934867 | -8.22743 | 3.84E-14 | 1.37E-12 | 21.61282 |
| LY75 | 1.607227 | 7.635549 | 8.207078 | 4.35E-14 | 1.54E-12 | 21.4923 |
| TRIB2 | 1.161388 | 7.937578 | 8.205873 | 4.38E-14 | 1.55E-12 | 21.48517 |
| LGALS1 | 1.393526 | 11.09574 | 8.204737 | 4.41E-14 | 1.56E-12 | 21.47844 |
| SEMA3C | 1.837587 | 8.268154 | 8.191419 | 4.78E-14 | 1.67E-12 | 21.39968 |
| GALE | 1.002865 | 7.456459 | 8.187941 | 4.88E-14 | 1.70E-12 | 21.37911 |
| CENPU | 1.400189 | 6.802681 | 8.175505 | 5.26E-14 | 1.82E-12 | 21.30563 |
| ZG16B | 1.491641 | 6.674879 | 8.17282 | 5.35E-14 | 1.85E-12 | 21.28977 |
| PDK4 | -1.62773 | 6.91785 | -8.16729 | 5.53E-14 | 1.90E-12 | 21.25714 |
| KITLG | 1.079911 | 8.631857 | 8.165154 | 5.60E-14 | 1.92E-12 | 21.2445 |
| CTA-445C9.15 | -1.68505 | 6.789781 | -8.14432 | 6.35E-14 | 2.16E-12 | 21.12154 |
| MMP1 | 2.74599 | 7.608021 | 8.142816 | 6.41E-14 | 2.17E-12 | 21.1127 |
| TGFB1I1 | 1.235713 | 8.145528 | 8.133217 | 6.79E-14 | 2.28E-12 | 21.05611 |
| CA4 | -1.22957 | 5.307127 | -8.12272 | 7.23E-14 | 2.40E-12 | 20.99426 |
| CCL20 | 2.037721 | 7.245314 | 8.121863 | 7.27E-14 | 2.40E-12 | 20.98922 |
| TRNP1 | 1.192441 | 7.61155 | 8.118888 | 7.40E-14 | 2.44E-12 | 20.9717 |
| MALL | 1.542659 | 7.918336 | 8.105878 | 8.01E-14 | 2.63E-12 | 20.89512 |
| TMEM133 | 1.179515 | 5.919811 | 8.09624 | 8.48E-14 | 2.76E-12 | 20.83842 |
| TPM4 | 1.033647 | 8.248388 | 8.094804 | 8.56E-14 | 2.78E-12 | 20.82997 |
| IFI44L | 1.557837 | 7.693952 | 8.0941 | 8.59E-14 | 2.78E-12 | 20.82583 |
| TRHDE | -1.84695 | 5.363748 | -8.08363 | 9.15E-14 | 2.94E-12 | 20.76426 |
| ENC1 | 1.104391 | 8.044632 | 8.077018 | 9.52E-14 | 3.04E-12 | 20.72543 |
| KDELC1 | 1.042508 | 6.075525 | 8.062679 | 1.04E-13 | 3.26E-12 | 20.64123 |
| CKS2 | 1.532172 | 7.325145 | 8.059877 | 1.06E-13 | 3.31E-12 | 20.62478 |
| PTPN12 | 1.064243 | 6.03582 | 8.043631 | 1.16E-13 | 3.61E-12 | 20.52948 |
| PERP | 1.299329 | 9.232493 | 8.038398 | 1.20E-13 | 3.72E-12 | 20.4988 |
| ELF4 | 1.083769 | 7.083037 | 8.030163 | 1.26E-13 | 3.88E-12 | 20.45054 |
| IFI6 | 1.217153 | 8.193747 | 8.026183 | 1.29E-13 | 3.95E-12 | 20.42722 |
| ZNF532 | 1.054925 | 7.029313 | 8.020214 | 1.34E-13 | 4.09E-12 | 20.39226 |
| DOCK5 | 1.022767 | 7.561205 | 8.019468 | 1.34E-13 | 4.09E-12 | 20.38789 |
| NCEH1 | 1.111475 | 6.627993 | 8.017734 | 1.36E-13 | 4.13E-12 | 20.37774 |
| NEK2 | 1.26306 | 5.285354 | 8.012064 | 1.41E-13 | 4.25E-12 | 20.34455 |
| RAPH1 | 1.081473 | 7.821377 | 8.01195 | 1.41E-13 | 4.25E-12 | 20.34388 |
| GRHL1 | 1.100683 | 6.306919 | 7.998764 | 1.52E-13 | 4.58E-12 | 20.26673 |
| HEPH | 1.761612 | 7.399437 | 7.987581 | 1.63E-13 | 4.85E-12 | 20.20135 |
| CMTM3 | 1.012912 | 8.174428 | 7.981864 | 1.68E-13 | 5.00E-12 | 20.16794 |
| BST2 | 1.08153 | 7.926699 | 7.976392 | 1.74E-13 | 5.13E-12 | 20.13598 |
| PNMA1 | 1.05692 | 8.043269 | 7.970567 | 1.80E-13 | 5.29E-12 | 20.10196 |
| LINC00342 | 1.129813 | 7.234475 | 7.963048 | 1.88E-13 | 5.51E-12 | 20.05807 |
| LRRN1 | 1.245562 | 4.887839 | 7.928827 | 2.31E-13 | 6.57E-12 | 19.85855 |
| ERO1L | 1.142108 | 8.145567 | 7.927932 | 2.32E-13 | 6.59E-12 | 19.85333 |
| ASPM | 1.495361 | 5.50651 | 7.925233 | 2.36E-13 | 6.67E-12 | 19.83762 |
| AFAP1-AS1 | 1.02581 | 6.247481 | 7.922678 | 2.40E-13 | 6.76E-12 | 19.82274 |
| GABBR1 /// UBD | 1.815199 | 9.004058 | 7.92113 | 2.42E-13 | 6.81E-12 | 19.81373 |
| C1orf116 | 1.346127 | 7.378506 | 7.913705 | 2.53E-13 | 7.08E-12 | 19.77051 |
| ID1 | 1.336184 | 8.767293 | 7.895615 | 2.81E-13 | 7.82E-12 | 19.6653 |
| HIST2H2AA3 /// HIST2H2AA4 | 1.104332 | 8.833188 | 7.89382 | 2.84E-13 | 7.89E-12 | 19.65487 |
| CCDC110 | -1.4715 | 4.448028 | -7.88268 | 3.04E-13 | 8.33E-12 | 19.59013 |
| COL8A2 | 1.240605 | 6.581307 | 7.860574 | 3.47E-13 | 9.31E-12 | 19.46183 |
| EPHA4 | 1.23895 | 5.365467 | 7.858294 | 3.51E-13 | 9.39E-12 | 19.44861 |
| RNF213 | 1.077165 | 7.448847 | 7.846178 | 3.77E-13 | 9.99E-12 | 19.37837 |
| TPM2 | 1.517946 | 9.303473 | 7.844515 | 3.81E-13 | 1.01E-11 | 19.36873 |
| KIF14 | 1.000009 | 5.625471 | 7.82824 | 4.20E-13 | 1.10E-11 | 19.27447 |
| MT1G | -1.22788 | 10.78753 | -7.82367 | 4.31E-13 | 1.13E-11 | 19.24804 |
| ADAM12 | 1.971326 | 6.415005 | 7.823576 | 4.31E-13 | 1.13E-11 | 19.24747 |
| GOLM1 | 1.198469 | 9.227285 | 7.818843 | 4.44E-13 | 1.16E-11 | 19.22009 |
| DACT1 | 1.33531 | 6.876296 | 7.81666 | 4.50E-13 | 1.17E-11 | 19.20746 |
| OAS2 | 1.209831 | 6.770542 | 7.812271 | 4.61E-13 | 1.19E-11 | 19.18207 |
| IGFL2 | 1.031437 | 6.293195 | 7.809797 | 4.68E-13 | 1.21E-11 | 19.16776 |
| CORO2A | 1.370998 | 6.432827 | 7.807176 | 4.75E-13 | 1.22E-11 | 19.15261 |
| TNFAIP6 | 1.548849 | 6.959081 | 7.805876 | 4.79E-13 | 1.23E-11 | 19.1451 |
| KLB | -1.22386 | 4.017637 | -7.79982 | 4.97E-13 | 1.27E-11 | 19.11007 |
| SEL1L | -1.82155 | 6.008041 | -7.7945 | 5.12E-13 | 1.31E-11 | 19.07935 |
| THY1 | 1.287537 | 8.333863 | 7.792587 | 5.18E-13 | 1.32E-11 | 19.0683 |
| AX746823 | 1.00787 | 6.73955 | 7.790337 | 5.25E-13 | 1.33E-11 | 19.05531 |
| KIAA1211 | 1.503441 | 6.461974 | 7.787785 | 5.33E-13 | 1.35E-11 | 19.04057 |
| HIST2H2BE | 1.053363 | 6.544769 | 7.777112 | 5.68E-13 | 1.43E-11 | 18.97896 |
| IFIT2 | 1.168849 | 7.052252 | 7.775689 | 5.73E-13 | 1.44E-11 | 18.97075 |
| LOC102723493 | -1.21552 | 4.889495 | -7.77551 | 5.73E-13 | 1.44E-11 | 18.96973 |
| TFPI | 1.202417 | 8.139367 | 7.759359 | 6.31E-13 | 1.55E-11 | 18.87658 |
| FA2H | 1.284543 | 6.973104 | 7.758365 | 6.34E-13 | 1.56E-11 | 18.87085 |
| ZWINT | 1.126653 | 7.198055 | 7.753549 | 6.53E-13 | 1.60E-11 | 18.84309 |
| CTNND2 | -1.26489 | 4.109309 | -7.74726 | 6.77E-13 | 1.65E-11 | 18.80688 |
| PRR11 | 1.184977 | 6.659118 | 7.736352 | 7.22E-13 | 1.74E-11 | 18.74406 |
| LOC100129129 | -1.02477 | 6.465794 | -7.72624 | 7.66E-13 | 1.83E-11 | 18.68588 |
| FLJ38379 | -1.38888 | 4.474663 | -7.72565 | 7.69E-13 | 1.83E-11 | 18.68248 |
| FAM129A | -1.06513 | 9.294065 | -7.72529 | 7.71E-13 | 1.83E-11 | 18.68042 |
| CXCL8 | 2.135448 | 8.656851 | 7.720263 | 7.94E-13 | 1.87E-11 | 18.6515 |
| GPR126 | 1.203081 | 6.942742 | 7.717062 | 8.09E-13 | 1.90E-11 | 18.63309 |
| LGALS2 | -1.63009 | 8.019658 | -7.71467 | 8.20E-13 | 1.92E-11 | 18.61937 |
| ASPHD2 | 1.04548 | 6.182155 | 7.69752 | 9.07E-13 | 2.09E-11 | 18.52083 |
| ATP4A | -1.08584 | 5.021368 | -7.69563 | 9.18E-13 | 2.11E-11 | 18.51 |
| S100A14 | 1.21981 | 7.53739 | 7.695179 | 9.20E-13 | 2.11E-11 | 18.50739 |
| MGC24103 | 1.323122 | 6.058644 | 7.686361 | 9.69E-13 | 2.21E-11 | 18.45678 |
| LRRC15 | 1.265339 | 6.594392 | 7.686182 | 9.70E-13 | 2.21E-11 | 18.45576 |
| DPYSL3 | 1.26532 | 8.865423 | 7.66775 | 1.08E-12 | 2.43E-11 | 18.35007 |
| SUGCT | 1.006631 | 6.345289 | 7.665508 | 1.09E-12 | 2.46E-11 | 18.33722 |
| ISM1 | 1.352385 | 6.87486 | 7.656664 | 1.15E-12 | 2.57E-11 | 18.28657 |
| RHPN2 | 1.275497 | 8.040025 | 7.653894 | 1.17E-12 | 2.61E-11 | 18.27071 |
| GAMT | -1.15894 | 6.688726 | -7.65054 | 1.20E-12 | 2.65E-11 | 18.25149 |
| MIA | 1.250189 | 6.78529 | 7.645354 | 1.23E-12 | 2.72E-11 | 18.22183 |
| TPX2 | 1.011219 | 6.264867 | 7.645059 | 1.23E-12 | 2.72E-11 | 18.22014 |
| CP | 1.803412 | 6.284076 | 7.637211 | 1.29E-12 | 2.83E-11 | 18.17525 |
| SKAP2 | 1.055958 | 6.646145 | 7.63193 | 1.33E-12 | 2.91E-11 | 18.14505 |
| CCNB1 | 1.240705 | 4.862382 | 7.629145 | 1.35E-12 | 2.94E-11 | 18.12913 |
| F11 | -1.08137 | 6.253403 | -7.62132 | 1.42E-12 | 3.05E-11 | 18.08441 |
| GPHA2 | -1.37983 | 6.534013 | -7.61227 | 1.50E-12 | 3.20E-11 | 18.03275 |
| TPST2 | -1.41824 | 9.236749 | -7.61147 | 1.50E-12 | 3.21E-11 | 18.02819 |
| DPP10 | -1.56173 | 3.989028 | -7.583 | 1.77E-12 | 3.72E-11 | 17.86576 |
| MAP4K4 | 1.025957 | 5.914489 | 7.580557 | 1.80E-12 | 3.76E-11 | 17.85185 |
| NRP2 | 1.230882 | 5.955167 | 7.574508 | 1.86E-12 | 3.87E-11 | 17.8174 |
| FAM83B | 1.095339 | 5.909237 | 7.556755 | 2.07E-12 | 4.22E-11 | 17.71635 |
| CTSB | 1.22639 | 9.121248 | 7.552871 | 2.11E-12 | 4.29E-11 | 17.69426 |
| F2R | 1.248121 | 7.981143 | 7.551711 | 2.13E-12 | 4.32E-11 | 17.68766 |
| FRMD6 | 1.198069 | 8.581382 | 7.547221 | 2.19E-12 | 4.41E-11 | 17.66214 |
| CTSK | 1.448387 | 9.401006 | 7.54552 | 2.21E-12 | 4.45E-11 | 17.65246 |
| LOX | 1.765085 | 7.970031 | 7.543985 | 2.23E-12 | 4.48E-11 | 17.64374 |
| MXD1 | 1.283505 | 7.250929 | 7.543049 | 2.24E-12 | 4.50E-11 | 17.63842 |
| MATN3 | 1.64111 | 4.506054 | 7.542345 | 2.25E-12 | 4.51E-11 | 17.63442 |
| SH3KBP1 | 1.074851 | 8.094189 | 7.542023 | 2.25E-12 | 4.52E-11 | 17.63259 |
| GPR137B | 1.082021 | 7.712539 | 7.536997 | 2.32E-12 | 4.63E-11 | 17.60404 |
| CRNDE | 1.311936 | 6.573754 | 7.536651 | 2.32E-12 | 4.63E-11 | 17.60207 |
| MIR4680 /// PDCD4 | -1.11662 | 7.807661 | -7.52882 | 2.43E-12 | 4.81E-11 | 17.55758 |
| MT1M | -1.46083 | 7.783323 | -7.52204 | 2.53E-12 | 4.98E-11 | 17.51912 |
| GALNT4 /// POC1B-GALNT4 | 1.126384 | 8.149371 | 7.512962 | 2.67E-12 | 5.23E-11 | 17.46761 |
| ANXA10 | 2.166597 | 8.534873 | 7.510471 | 2.71E-12 | 5.28E-11 | 17.45348 |
| ST6GALNAC1 | 1.844634 | 6.607812 | 7.510246 | 2.71E-12 | 5.29E-11 | 17.45221 |
| TDH | -1.45335 | 3.542833 | -7.49748 | 2.92E-12 | 5.65E-11 | 17.37988 |
| PLXDC2 | 1.359737 | 6.33174 | 7.493308 | 2.99E-12 | 5.77E-11 | 17.35622 |
| SPX | -2.50263 | 4.91493 | -7.48188 | 3.19E-12 | 6.09E-11 | 17.29153 |
| PM20D1 | -1.76858 | 5.003691 | -7.48176 | 3.20E-12 | 6.09E-11 | 17.29081 |
| DTL | 1.163679 | 5.636918 | 7.481493 | 3.20E-12 | 6.09E-11 | 17.28933 |
| CORIN | 1.290546 | 4.608205 | 7.475136 | 3.32E-12 | 6.28E-11 | 17.25336 |
| SPACA3 | -1.04552 | 4.565142 | -7.46833 | 3.45E-12 | 6.51E-11 | 17.21485 |
| TNFSF10 | 1.013711 | 7.969259 | 7.466132 | 3.50E-12 | 6.58E-11 | 17.20244 |
| DUOX2 | 2.141633 | 7.048482 | 7.463657 | 3.55E-12 | 6.67E-11 | 17.18845 |
| ISLR | 1.362793 | 7.59318 | 7.458275 | 3.66E-12 | 6.83E-11 | 17.15804 |
| TMEM45B | 1.796865 | 6.141056 | 7.450047 | 3.84E-12 | 7.09E-11 | 17.11156 |
| KRT6B | 1.878991 | 6.409772 | 7.446781 | 3.91E-12 | 7.19E-11 | 17.09312 |
| TWIST1 | 1.348119 | 6.209252 | 7.440648 | 4.05E-12 | 7.40E-11 | 17.0585 |
| PSAT1 | -1.71551 | 7.455325 | -7.42983 | 4.32E-12 | 7.84E-11 | 16.99749 |
| PGK1 | 1.007327 | 9.200733 | 7.428929 | 4.34E-12 | 7.87E-11 | 16.9924 |
| ANXA1 | 1.294254 | 10.37694 | 7.425849 | 4.42E-12 | 8.00E-11 | 16.97503 |
| ERO1LB | -1.67621 | 7.866875 | -7.41487 | 4.70E-12 | 8.44E-11 | 16.91316 |
| LAMA4 | 1.39047 | 7.690257 | 7.40679 | 4.93E-12 | 8.81E-11 | 16.86766 |
| KIAA0101 | 1.131173 | 8.102182 | 7.40642 | 4.94E-12 | 8.82E-11 | 16.86558 |
| VILL | 1.134643 | 7.08799 | 7.401268 | 5.09E-12 | 9.03E-11 | 16.83659 |
| VSIG2 | 1.242199 | 6.539033 | 7.393755 | 5.31E-12 | 9.35E-11 | 16.79432 |
| FAM72A /// FAM72B /// FAM72C /// FAM72D | 1.20137 | 6.050659 | 7.38871 | 5.47E-12 | 9.59E-11 | 16.76594 |
| BAIAP2L1 | 1.236437 | 7.947624 | 7.387868 | 5.50E-12 | 9.63E-11 | 16.76121 |
| BACE2 | 1.123161 | 6.693761 | 7.379376 | 5.77E-12 | 1.00E-10 | 16.71348 |
| GUCA1C | -1.96678 | 4.727841 | -7.37918 | 5.78E-12 | 1.00E-10 | 16.71236 |
| MAD2L1 | 1.24302 | 5.904864 | 7.378567 | 5.80E-12 | 1.00E-10 | 16.70894 |
| DNASE1 | -1.689 | 5.984679 | -7.37168 | 6.03E-12 | 1.04E-10 | 16.67025 |
| LINC01094 | 1.09653 | 4.958853 | 7.370449 | 6.08E-12 | 1.04E-10 | 16.66334 |
| S100A2 | 1.670091 | 6.537388 | 7.370067 | 6.09E-12 | 1.04E-10 | 16.66119 |
| DLGAP5 | 1.21783 | 5.002724 | 7.36652 | 6.22E-12 | 1.06E-10 | 16.64128 |
| EPHX2 | -1.02321 | 6.570003 | -7.36357 | 6.32E-12 | 1.08E-10 | 16.62472 |
| CDCA7 | 1.534956 | 7.215267 | 7.355401 | 6.63E-12 | 1.12E-10 | 16.57889 |
| PXDN | 1.166248 | 8.579311 | 7.345936 | 7.00E-12 | 1.18E-10 | 16.52582 |
| ADAM28 | 1.360928 | 6.65698 | 7.344656 | 7.05E-12 | 1.18E-10 | 16.51865 |
| TNFAIP2 | 1.027752 | 8.196019 | 7.340694 | 7.21E-12 | 1.21E-10 | 16.49644 |
| SLCO4A1 | 1.048196 | 6.743438 | 7.334835 | 7.46E-12 | 1.24E-10 | 16.46362 |
| KRT8 | 1.181259 | 9.62423 | 7.332975 | 7.54E-12 | 1.26E-10 | 16.4532 |
| NARR /// RAB34 | 1.07956 | 7.958024 | 7.32489 | 7.89E-12 | 1.31E-10 | 16.40794 |
| C5 | -1.33117 | 6.417827 | -7.32408 | 7.93E-12 | 1.31E-10 | 16.40339 |
| CEP170 /// CEP170P1 | 1.008931 | 8.570477 | 7.305285 | 8.83E-12 | 1.44E-10 | 16.29829 |
| HMGA2 | 1.243464 | 4.86359 | 7.303515 | 8.92E-12 | 1.46E-10 | 16.2884 |
| GPR110 | 1.121126 | 4.243883 | 7.303125 | 8.94E-12 | 1.46E-10 | 16.28623 |
| SERPINA1 | 1.502255 | 10.31917 | 7.300144 | 9.10E-12 | 1.48E-10 | 16.26957 |
| LUM | 1.490765 | 7.847794 | 7.284401 | 9.95E-12 | 1.60E-10 | 16.18167 |
| SOX9 | 1.321307 | 8.781904 | 7.284184 | 9.97E-12 | 1.60E-10 | 16.18046 |
| CENPK | 1.273043 | 4.838098 | 7.278735 | 1.03E-11 | 1.65E-10 | 16.15006 |
| PDPN | 1.081699 | 6.221723 | 7.276799 | 1.04E-11 | 1.66E-10 | 16.13926 |
| IL18 | 1.038008 | 6.651662 | 7.273221 | 1.06E-11 | 1.70E-10 | 16.11931 |
| SLC12A2 | 1.102809 | 7.653461 | 7.270116 | 1.08E-11 | 1.72E-10 | 16.102 |
| CDKN2B | 1.53536 | 6.72198 | 7.254285 | 1.18E-11 | 1.87E-10 | 16.01381 |
| MT1F | -1.07773 | 9.764825 | -7.25171 | 1.20E-11 | 1.89E-10 | 15.99945 |
| ITGBL1 | 1.919921 | 7.521463 | 7.251429 | 1.20E-11 | 1.90E-10 | 15.99791 |
| SERPINI1 | -1.1245 | 6.999316 | -7.24793 | 1.23E-11 | 1.93E-10 | 15.97843 |
| IFIT3 | 1.109225 | 8.365482 | 7.246783 | 1.23E-11 | 1.94E-10 | 15.97205 |
| CCKBR | -1.281 | 5.347046 | -7.23015 | 1.36E-11 | 2.09E-10 | 15.87953 |
| PRR15 | 1.202595 | 6.374675 | 7.220783 | 1.43E-11 | 2.19E-10 | 15.82751 |
| GREM1 | 2.168101 | 8.13863 | 7.216989 | 1.46E-11 | 2.23E-10 | 15.80644 |
| SLC16A1 | 1.14024 | 5.736347 | 7.214169 | 1.49E-11 | 2.25E-10 | 15.79078 |
| SLC39A5 | -1.16443 | 7.881446 | -7.20918 | 1.53E-11 | 2.31E-10 | 15.76308 |
| ITGB8 | 1.137077 | 9.06833 | 7.203328 | 1.58E-11 | 2.38E-10 | 15.73063 |
| GNMT | -2.14944 | 6.009864 | -7.20099 | 1.60E-11 | 2.40E-10 | 15.71768 |
| RARRES3 | 1.191818 | 8.236429 | 7.195931 | 1.65E-11 | 2.46E-10 | 15.68962 |
| INPP4B | 1.154617 | 6.775649 | 7.195607 | 1.65E-11 | 2.46E-10 | 15.68782 |
| ARL4C | 1.34192 | 8.790007 | 7.181975 | 1.78E-11 | 2.63E-10 | 15.61229 |
| EGLN3 | 1.240813 | 6.329575 | 7.181742 | 1.79E-11 | 2.63E-10 | 15.611 |
| ANXA8 /// ANXA8L1 | 1.322507 | 7.11763 | 7.178929 | 1.82E-11 | 2.66E-10 | 15.59543 |
| LINC01133 | 1.576695 | 6.125301 | 7.177703 | 1.83E-11 | 2.68E-10 | 15.58864 |
| SERPINB2 | 1.635537 | 5.500631 | 7.176329 | 1.84E-11 | 2.70E-10 | 15.58104 |
| GBP1 | 1.457131 | 8.394526 | 7.173014 | 1.88E-11 | 2.73E-10 | 15.56269 |
| PTPRR | 1.131389 | 4.46905 | 7.159529 | 2.03E-11 | 2.92E-10 | 15.4881 |
| CFB | 1.201849 | 8.09334 | 7.157051 | 2.06E-11 | 2.95E-10 | 15.47441 |
| KCNJ5 | -1.32653 | 6.405344 | -7.15515 | 2.08E-11 | 2.98E-10 | 15.4639 |
| DPP10-AS1 | -1.03728 | 5.387213 | -7.14783 | 2.17E-11 | 3.09E-10 | 15.42345 |
| GBP2 | 1.205824 | 7.203365 | 7.147454 | 2.17E-11 | 3.09E-10 | 15.42138 |
| FAM83D | 1.364581 | 6.266357 | 7.138397 | 2.28E-11 | 3.23E-10 | 15.37138 |
| IL1R2 | 1.374087 | 7.107725 | 7.137978 | 2.29E-11 | 3.24E-10 | 15.36907 |
| GLS2 | -1.1083 | 5.379977 | -7.1339 | 2.34E-11 | 3.30E-10 | 15.34658 |
| PRLR | -1.16101 | 4.879988 | -7.13276 | 2.36E-11 | 3.32E-10 | 15.34029 |
| VSIG1 | 2.000318 | 5.896384 | 7.12471 | 2.47E-11 | 3.45E-10 | 15.29588 |
| CLDN1 | 1.350262 | 8.780917 | 7.084157 | 3.10E-11 | 4.19E-10 | 15.07266 |
| TREM1 | 1.102615 | 6.612106 | 7.081062 | 3.16E-11 | 4.26E-10 | 15.05565 |
| CDC42EP5 | 1.221627 | 7.658634 | 7.078198 | 3.21E-11 | 4.32E-10 | 15.03992 |
| DHRS9 | 1.527139 | 6.607046 | 7.070615 | 3.35E-11 | 4.48E-10 | 14.99827 |
| 0 | -1.92967 | 7.18624 | -7.06177 | 3.52E-11 | 4.67E-10 | 14.94973 |
| RRM2 | 1.304263 | 7.19209 | 7.054102 | 3.67E-11 | 4.84E-10 | 14.90768 |
| FBN1 | 1.457093 | 9.746908 | 7.052084 | 3.72E-11 | 4.88E-10 | 14.89661 |
| LAMP5 | 1.313318 | 7.13257 | 7.037113 | 4.04E-11 | 5.24E-10 | 14.81459 |
| ACTA2 | 1.237064 | 11.55235 | 7.028122 | 4.25E-11 | 5.46E-10 | 14.76538 |
| BUB1B | 1.073032 | 6.052606 | 7.028057 | 4.25E-11 | 5.46E-10 | 14.76503 |
| PRRX1 | 1.429952 | 8.532695 | 7.019313 | 4.47E-11 | 5.67E-10 | 14.7172 |
| KLK7 | 1.322713 | 6.946192 | 7.017313 | 4.52E-11 | 5.73E-10 | 14.70627 |
| IFIT1 | 1.079754 | 7.8468 | 7.005448 | 4.83E-11 | 6.05E-10 | 14.64144 |
| NUCB2 | -1.22072 | 9.541906 | -6.99719 | 5.06E-11 | 6.31E-10 | 14.59636 |
| LPAR3 | -1.16944 | 4.097348 | -6.99014 | 5.26E-11 | 6.52E-10 | 14.55789 |
| TMEM52 | -1.61348 | 5.300504 | -6.98624 | 5.38E-11 | 6.65E-10 | 14.53662 |
| B3GNT5 | 1.002328 | 8.425747 | 6.975761 | 5.70E-11 | 7.00E-10 | 14.4795 |
| LINC00339 | -1.12153 | 7.058086 | -6.96422 | 6.08E-11 | 7.40E-10 | 14.41665 |
| F8 | -1.04449 | 7.368276 | -6.95848 | 6.28E-11 | 7.59E-10 | 14.38541 |
| BNIP3 | -1.40869 | 9.120504 | -6.95742 | 6.32E-11 | 7.62E-10 | 14.37964 |
| BACE1 | -1.34322 | 8.559762 | -6.95101 | 6.55E-11 | 7.86E-10 | 14.34478 |
| KIF11 | 1.082343 | 4.983382 | 6.944643 | 6.78E-11 | 8.08E-10 | 14.31017 |
| C5orf46 | 1.026828 | 5.908887 | 6.93914 | 7.00E-11 | 8.29E-10 | 14.28027 |
| HOXA10 | 1.078864 | 4.680595 | 6.936329 | 7.11E-11 | 8.40E-10 | 14.265 |
| AOC1 | 1.883418 | 6.629771 | 6.934237 | 7.19E-11 | 8.47E-10 | 14.25364 |
| ENTPD1 | 1.07559 | 6.576097 | 6.909241 | 8.26E-11 | 9.60E-10 | 14.11806 |
| SHROOM3 | 1.082133 | 7.654867 | 6.907322 | 8.35E-11 | 9.68E-10 | 14.10766 |
| ASPN | 1.338726 | 7.521282 | 6.904695 | 8.47E-11 | 9.79E-10 | 14.09343 |
| CLIC3 | 1.113239 | 6.016129 | 6.894055 | 8.99E-11 | 1.03E-09 | 14.03582 |
| PLAC8 | 1.837229 | 8.51548 | 6.890423 | 9.17E-11 | 1.05E-09 | 14.01617 |
| DPCR1 | 1.84946 | 6.377369 | 6.883747 | 9.52E-11 | 1.08E-09 | 13.98006 |
| CXCL3 | 1.43642 | 6.254222 | 6.869871 | 1.03E-10 | 1.16E-09 | 13.90507 |
| AKAP7 | -1.28109 | 8.057063 | -6.86753 | 1.04E-10 | 1.17E-09 | 13.89244 |
| LXN | 1.125081 | 9.159098 | 6.864586 | 1.06E-10 | 1.19E-09 | 13.87654 |
| TNFRSF11B | 1.186944 | 5.574952 | 6.861455 | 1.08E-10 | 1.20E-09 | 13.85964 |
| ABCG1 | 1.152144 | 6.709168 | 6.860513 | 1.08E-10 | 1.21E-09 | 13.85455 |
| CBS | -1.57631 | 5.972527 | -6.8552 | 1.12E-10 | 1.24E-09 | 13.82588 |
| MMP2 | 1.276477 | 9.739954 | 6.853799 | 1.12E-10 | 1.25E-09 | 13.81833 |
| LOC101928916 /// NNMT | 1.419665 | 10.37444 | 6.850168 | 1.15E-10 | 1.27E-09 | 13.79875 |
| BICD1 | 1.058974 | 5.190816 | 6.836153 | 1.24E-10 | 1.36E-09 | 13.72322 |
| DMD | -1.32912 | 7.668595 | -6.8296 | 1.28E-10 | 1.40E-09 | 13.68792 |
| KRT18 | 1.034682 | 10.95696 | 6.825843 | 1.31E-10 | 1.43E-09 | 13.66772 |
| UGT1A1 /// UGT1A10 /// UGT1A3 /// UGT1A4 /// UGT1A5 /// UGT1A6 /// UGT1A7 /// UGT1A8 /// UGT1A9 | 1.780357 | 6.21281 | 6.824869 | 1.32E-10 | 1.43E-09 | 13.66248 |
| SLC1A2 | -1.64993 | 5.434684 | -6.82286 | 1.33E-10 | 1.45E-09 | 13.65168 |
| ABAT | -1.4883 | 8.34167 | -6.82093 | 1.35E-10 | 1.46E-09 | 13.64128 |
| CDC20 | 1.071742 | 6.072216 | 6.806331 | 1.46E-10 | 1.56E-09 | 13.56282 |
| FAM150B | -1.7034 | 6.619112 | -6.80189 | 1.50E-10 | 1.60E-09 | 13.53898 |
| TNFSF11 | 1.197977 | 4.95734 | 6.79811 | 1.53E-10 | 1.63E-09 | 13.51867 |
| SFRP2 | 1.775057 | 9.450082 | 6.79622 | 1.54E-10 | 1.64E-09 | 13.50853 |
| XDH | 1.542472 | 6.657033 | 6.795929 | 1.55E-10 | 1.64E-09 | 13.50697 |
| SLC43A1 | -1.27886 | 7.669851 | -6.79133 | 1.59E-10 | 1.68E-09 | 13.48229 |
| MCOLN3 | -1.05496 | 6.645821 | -6.77596 | 1.73E-10 | 1.80E-09 | 13.39986 |
| ABRACL | 1.023589 | 7.832633 | 6.775676 | 1.73E-10 | 1.80E-09 | 13.39836 |
| FUT2 | 1.016135 | 7.17802 | 6.761867 | 1.87E-10 | 1.92E-09 | 13.32442 |
| RBPJL | -1.73325 | 6.242541 | -6.7475 | 2.02E-10 | 2.06E-09 | 13.24756 |
| GLT8D2 | 1.326548 | 7.653424 | 6.736475 | 2.15E-10 | 2.17E-09 | 13.18869 |
| LIFR | -1.07416 | 5.477188 | -6.72327 | 2.31E-10 | 2.31E-09 | 13.11823 |
| PLS1 | 1.463671 | 8.881854 | 6.720409 | 2.34E-10 | 2.34E-09 | 13.10297 |
| CLEC5A | 1.049704 | 5.272171 | 6.716856 | 2.39E-10 | 2.37E-09 | 13.08403 |
| SLC22A3 | 1.042942 | 4.424462 | 6.71087 | 2.47E-10 | 2.44E-09 | 13.05213 |
| TEX11 | -2.38751 | 6.694481 | -6.7081 | 2.51E-10 | 2.47E-09 | 13.03736 |
| MACROD2 | 1.067908 | 5.490554 | 6.69729 | 2.66E-10 | 2.60E-09 | 12.97983 |
| CTH | -1.12766 | 5.894415 | -6.69376 | 2.71E-10 | 2.65E-09 | 12.96105 |
| TRIM50 | -1.04566 | 5.31434 | -6.68624 | 2.82E-10 | 2.75E-09 | 12.92108 |
| MACC1 | 1.059239 | 5.526066 | 6.662652 | 3.21E-10 | 3.05E-09 | 12.7958 |
| PLCE1 | -1.11867 | 6.641478 | -6.64584 | 3.52E-10 | 3.30E-09 | 12.7067 |
| RAB25 | 1.212264 | 8.345241 | 6.644824 | 3.54E-10 | 3.31E-09 | 12.70131 |
| COCH | -1.44877 | 6.068184 | -6.64271 | 3.58E-10 | 3.34E-09 | 12.69012 |
| MMP12 | 2.077928 | 7.066817 | 6.640082 | 3.63E-10 | 3.38E-09 | 12.6762 |
| CTD-2377D24.6 | 1.122959 | 6.026405 | 6.631193 | 3.81E-10 | 3.52E-09 | 12.62916 |
| TRIM14 | 1.012867 | 7.838776 | 6.617461 | 4.11E-10 | 3.76E-09 | 12.55657 |
| COL6A2 | 1.242433 | 8.933894 | 6.592484 | 4.70E-10 | 4.21E-09 | 12.42477 |
| TSPAN8 | 1.700309 | 11.20608 | 6.584638 | 4.91E-10 | 4.37E-09 | 12.38342 |
| FZD7 | 1.012148 | 7.095271 | 6.581527 | 4.99E-10 | 4.42E-09 | 12.36704 |
| SEMA6D | -1.01986 | 6.559133 | -6.57629 | 5.14E-10 | 4.53E-09 | 12.33948 |
| GPNMB | 1.35959 | 5.575961 | 6.571978 | 5.26E-10 | 4.63E-09 | 12.31678 |
| TOX3 | 1.483484 | 7.121284 | 6.567334 | 5.39E-10 | 4.72E-09 | 12.29236 |
| LGALS4 | 1.797251 | 9.424205 | 6.556147 | 5.73E-10 | 4.98E-09 | 12.23355 |
| GATA3 | 1.066435 | 7.238151 | 6.555875 | 5.73E-10 | 4.98E-09 | 12.23213 |
| NT5E | 1.249089 | 7.277591 | 6.555524 | 5.75E-10 | 4.99E-09 | 12.23029 |
| DSG2 | 1.069869 | 9.700673 | 6.520672 | 6.93E-10 | 5.86E-09 | 12.0475 |
| AQP12A /// AQP12B | -1.91464 | 5.948174 | -6.50851 | 7.40E-10 | 6.20E-09 | 11.98387 |
| ALOX5AP | 1.232788 | 8.989421 | 6.506952 | 7.47E-10 | 6.24E-09 | 11.97571 |
| PDZK1IP1 | 1.279106 | 8.248742 | 6.496273 | 7.91E-10 | 6.54E-09 | 11.91989 |
| MYL9 | 1.390961 | 8.933235 | 6.478143 | 8.71E-10 | 7.10E-09 | 11.82527 |
| EGF | -1.85178 | 5.540619 | -6.46236 | 9.48E-10 | 7.65E-09 | 11.74301 |
| TRIM31 | 1.336117 | 4.808776 | 6.44463 | 1.04E-09 | 8.29E-09 | 11.65077 |
| BC017398 /// CTD-2314B22.3 /// DQ786293 /// DUXAP10 /// LL22NC03-N64E9.1 | 1.067559 | 6.869742 | 6.442493 | 1.05E-09 | 8.37E-09 | 11.63967 |
| IFI16 | 1.168916 | 8.25389 | 6.433056 | 1.11E-09 | 8.72E-09 | 11.59064 |
| AOX1 | -1.616 | 7.679394 | -6.42907 | 1.13E-09 | 8.89E-09 | 11.56996 |
| PSCA | 1.557512 | 5.905875 | 6.423505 | 1.17E-09 | 9.13E-09 | 11.54107 |
| EPS8L3 | 1.281233 | 6.872785 | 6.419483 | 1.19E-09 | 9.29E-09 | 11.52021 |
| SYT13 | 1.218102 | 8.278069 | 6.41931 | 1.19E-09 | 9.30E-09 | 11.51931 |
| COTL1 | 1.034053 | 8.151855 | 6.407705 | 1.27E-09 | 9.80E-09 | 11.45917 |
| GAS2 | -1.16147 | 6.446882 | -6.40123 | 1.31E-09 | 1.01E-08 | 11.42562 |
| TMED6 | -2.42785 | 7.08936 | -6.39821 | 1.34E-09 | 1.02E-08 | 11.41002 |
| ROBO1 | 1.025911 | 7.754719 | 6.395512 | 1.36E-09 | 1.04E-08 | 11.39605 |
| SLC39A8 | -1.09718 | 8.099319 | -6.36887 | 1.56E-09 | 1.17E-08 | 11.2584 |
| GXYLT2 | 1.116193 | 7.708188 | 6.364952 | 1.59E-09 | 1.19E-08 | 11.23817 |
| GBP3 | 1.252177 | 8.654142 | 6.362448 | 1.62E-09 | 1.21E-08 | 11.22526 |
| HMCN1 | 1.126552 | 6.869252 | 6.357354 | 1.66E-09 | 1.24E-08 | 11.199 |
| CSTA | 1.272944 | 7.908184 | 6.35729 | 1.66E-09 | 1.24E-08 | 11.19866 |
| RGN | -1.08064 | 6.797493 | -6.3549 | 1.68E-09 | 1.25E-08 | 11.18635 |
| ACSL5 | 1.300252 | 8.074077 | 6.353754 | 1.69E-09 | 1.26E-08 | 11.18045 |
| SCNN1A | 1.155138 | 8.523155 | 6.338312 | 1.84E-09 | 1.35E-08 | 11.10094 |
| CDS1 | 1.03106 | 7.408965 | 6.327274 | 1.95E-09 | 1.41E-08 | 11.04418 |
| EHF | 1.196632 | 9.51843 | 6.322544 | 2.00E-09 | 1.44E-08 | 11.01988 |
| PHLDA1 | 1.039758 | 8.232876 | 6.322105 | 2.00E-09 | 1.45E-08 | 11.01763 |
| ESRRG | -1.00467 | 5.731 | -6.32208 | 2.00E-09 | 1.45E-08 | 11.01751 |
| PDIA2 | -2.25809 | 7.879969 | -6.3028 | 2.22E-09 | 1.58E-08 | 10.91857 |
| AQP8 | -2.47332 | 7.420745 | -6.29814 | 2.27E-09 | 1.61E-08 | 10.89469 |
| BNC2 | 1.018946 | 6.539074 | 6.288769 | 2.39E-09 | 1.68E-08 | 10.84668 |
| HMMR | 1.016324 | 5.973873 | 6.281168 | 2.48E-09 | 1.74E-08 | 10.80779 |
| PCSK5 | 1.049176 | 7.082922 | 6.278021 | 2.53E-09 | 1.76E-08 | 10.79169 |
| PNMA2 | 1.111056 | 7.10182 | 6.258719 | 2.80E-09 | 1.92E-08 | 10.69308 |
| RARRES1 | 1.570704 | 8.358195 | 6.256736 | 2.82E-09 | 1.94E-08 | 10.68296 |
| RP11-320N7.2 | -1.49285 | 5.28083 | -6.23731 | 3.13E-09 | 2.11E-08 | 10.58391 |
| AF070581 | -1.5239 | 6.466188 | -6.23655 | 3.14E-09 | 2.11E-08 | 10.58004 |
| MGP | 1.40995 | 6.971054 | 6.208861 | 3.63E-09 | 2.40E-08 | 10.43927 |
| MIR143HG | 1.09396 | 6.466214 | 6.198033 | 3.84E-09 | 2.52E-08 | 10.38432 |
| ACADL | -1.36293 | 5.632285 | -6.18932 | 4.02E-09 | 2.62E-08 | 10.34015 |
| PSMB9 | 1.059731 | 8.294885 | 6.186203 | 4.09E-09 | 2.65E-08 | 10.32435 |
| NCF2 | 1.107782 | 7.235233 | 6.178262 | 4.26E-09 | 2.74E-08 | 10.28414 |
| ANPEP | -1.86011 | 8.818725 | -6.17022 | 4.44E-09 | 2.84E-08 | 10.24347 |
| TAGLN | 1.11748 | 10.83336 | 6.160764 | 4.67E-09 | 2.96E-08 | 10.19565 |
| GPR87 | 1.192115 | 4.22148 | 6.154442 | 4.82E-09 | 3.04E-08 | 10.16372 |
| CCDC109B | 1.012413 | 7.336813 | 6.15068 | 4.92E-09 | 3.09E-08 | 10.14473 |
| DPEP1 | -1.08746 | 7.55671 | -6.14821 | 4.98E-09 | 3.13E-08 | 10.13227 |
| ALB | -2.9748 | 6.901923 | -6.12039 | 5.76E-09 | 3.53E-08 | 9.992111 |
| CFH /// CFHR1 | 1.089337 | 9.090464 | 6.110438 | 6.06E-09 | 3.69E-08 | 9.942058 |
| KCNK3 | -1.40244 | 5.900895 | -6.10275 | 6.31E-09 | 3.81E-08 | 9.903451 |
| ARL14 | 1.610696 | 7.522264 | 6.073897 | 7.32E-09 | 4.33E-08 | 9.758777 |
| KIAA1324 | -1.67377 | 8.297216 | -6.05789 | 7.95E-09 | 4.66E-08 | 9.678699 |
| LOC283075 | -1.05985 | 3.838888 | -6.04818 | 8.36E-09 | 4.87E-08 | 9.630203 |
| MUC16 | 1.131734 | 4.894303 | 6.036975 | 8.86E-09 | 5.12E-08 | 9.574318 |
| SUCNR1 | 1.096487 | 5.605234 | 6.032431 | 9.07E-09 | 5.21E-08 | 9.551666 |
| SULT1C2 | 1.63787 | 6.600791 | 6.027658 | 9.29E-09 | 5.33E-08 | 9.527885 |
| ERBB3 | 1.180888 | 9.273949 | 6.02639 | 9.35E-09 | 5.36E-08 | 9.521568 |
| RP3-428L16.2 | 1.227232 | 6.129486 | 6.018167 | 9.76E-09 | 5.56E-08 | 9.480635 |
| HTR2B | 1.240501 | 5.708131 | 6.009456 | 1.02E-08 | 5.77E-08 | 9.437315 |
| BIRC3 | 1.06737 | 8.343993 | 6.00012 | 1.07E-08 | 6.01E-08 | 9.390926 |
| DLEU2 | 1.105201 | 6.847305 | 5.991613 | 1.12E-08 | 6.24E-08 | 9.348704 |
| SFRP4 | 1.675951 | 7.295024 | 5.987125 | 1.14E-08 | 6.37E-08 | 9.326442 |
| C9orf152 | 1.235704 | 6.135093 | 5.98693 | 1.15E-08 | 6.38E-08 | 9.325475 |
| LMO3 | -1.18843 | 7.039146 | -5.97643 | 1.21E-08 | 6.67E-08 | 9.273434 |
| ZG16 | -1.12752 | 5.74586 | -5.95342 | 1.36E-08 | 7.38E-08 | 9.159641 |
| PROM2 | 1.095588 | 7.490937 | 5.943696 | 1.43E-08 | 7.70E-08 | 9.111606 |
| MUC5B | 1.23097 | 6.534286 | 5.928918 | 1.54E-08 | 8.21E-08 | 9.038739 |
| PLXNC1 | 1.033933 | 7.778269 | 5.92258 | 1.59E-08 | 8.44E-08 | 9.007523 |
| SERPINB3 | 1.453113 | 3.876483 | 5.902266 | 1.76E-08 | 9.21E-08 | 8.907629 |
| CXCR4 | 1.499179 | 9.9959 | 5.898508 | 1.80E-08 | 9.37E-08 | 8.889174 |
| ZNF521 | 1.183797 | 6.053443 | 5.895296 | 1.83E-08 | 9.50E-08 | 8.873408 |
| CCL18 | 1.446537 | 8.044246 | 5.894626 | 1.83E-08 | 9.53E-08 | 8.87012 |
| CXCL14 | 1.455261 | 9.090746 | 5.872858 | 2.05E-08 | 1.05E-07 | 8.763423 |
| SPP1 | 1.357079 | 11.33336 | 5.866902 | 2.11E-08 | 1.07E-07 | 8.734277 |
| CLDN2 | 1.082613 | 6.799551 | 5.855105 | 2.24E-08 | 1.13E-07 | 8.676604 |
| MOXD1 | 1.114397 | 7.317212 | 5.854655 | 2.24E-08 | 1.13E-07 | 8.674407 |
| GNB4 | 1.074571 | 8.498177 | 5.822469 | 2.64E-08 | 1.30E-07 | 8.517462 |
| BASP1 | 1.075385 | 8.839233 | 5.820966 | 2.66E-08 | 1.31E-07 | 8.510149 |
| CXCL12 | -1.0502 | 9.659873 | -5.80854 | 2.83E-08 | 1.39E-07 | 8.449729 |
| AREG | 1.293775 | 7.123055 | 5.806432 | 2.86E-08 | 1.40E-07 | 8.439483 |
| RNF186 | -1.17068 | 6.629484 | -5.80328 | 2.90E-08 | 1.42E-07 | 8.424196 |
| SLC1A1 | 1.204096 | 7.356603 | 5.775766 | 3.33E-08 | 1.60E-07 | 8.29078 |
| VEPH1 | -1.00611 | 5.14648 | -5.73555 | 4.08E-08 | 1.91E-07 | 8.096562 |
| CYP3A5 | 1.446063 | 7.909526 | 5.724598 | 4.30E-08 | 2.01E-07 | 8.043854 |
| GDA | 1.353066 | 7.019506 | 5.719209 | 4.42E-08 | 2.05E-07 | 8.01794 |
| KRT6A | 1.427947 | 4.298194 | 5.706561 | 4.71E-08 | 2.17E-07 | 7.957171 |
| ISG20 | 1.043208 | 7.544017 | 5.702226 | 4.81E-08 | 2.21E-07 | 7.936366 |
| MUC5AC | 1.349705 | 6.615405 | 5.679594 | 5.38E-08 | 2.45E-07 | 7.827928 |
| HSD17B6 | 1.073356 | 4.494587 | 5.673016 | 5.56E-08 | 2.52E-07 | 7.796464 |
| SPON1 | 1.297623 | 7.605003 | 5.664354 | 5.80E-08 | 2.62E-07 | 7.755073 |
| CEACAM7 | 1.476959 | 6.476805 | 5.638006 | 6.61E-08 | 2.94E-07 | 7.629436 |
| SCIN | 1.188276 | 5.539726 | 5.63636 | 6.66E-08 | 2.96E-07 | 7.6216 |
| AGR3 | 1.653538 | 8.09053 | 5.625248 | 7.04E-08 | 3.11E-07 | 7.568746 |
| MUC1 | 1.252578 | 9.239948 | 5.619421 | 7.24E-08 | 3.19E-07 | 7.54106 |
| ELF3 | 1.014209 | 7.546568 | 5.61057 | 7.56E-08 | 3.31E-07 | 7.499042 |
| LOC101930067 | -1.36529 | 4.103234 | -5.60304 | 7.85E-08 | 3.42E-07 | 7.463315 |
| MSRB3 | 1.021921 | 8.458012 | 5.59587 | 8.13E-08 | 3.53E-07 | 7.429362 |
| PROM1 | 1.384581 | 8.666635 | 5.584121 | 8.61E-08 | 3.70E-07 | 7.373763 |
| SERPINI2 | -2.8458 | 7.276542 | -5.57937 | 8.81E-08 | 3.78E-07 | 7.351316 |
| ERP27 | -2.35256 | 9.036391 | -5.56051 | 9.67E-08 | 4.10E-07 | 7.26227 |
| KLK1 | -1.96243 | 8.38744 | -5.53992 | 1.07E-07 | 4.48E-07 | 7.165333 |
| OLFM4 | 2.079733 | 10.19726 | 5.50656 | 1.26E-07 | 5.15E-07 | 7.008775 |
| MFAP5 | 1.33296 | 7.202802 | 5.502893 | 1.28E-07 | 5.23E-07 | 6.991609 |
| CTRL | -2.78749 | 8.292045 | -5.48147 | 1.42E-07 | 5.73E-07 | 6.8915 |
| ODAM | 1.022699 | 4.918084 | 5.466561 | 1.53E-07 | 6.12E-07 | 6.821958 |
| LGR5 | 1.069617 | 4.709999 | 5.465232 | 1.54E-07 | 6.16E-07 | 6.815766 |
| VNN1 | 1.549974 | 7.056558 | 5.433503 | 1.79E-07 | 7.06E-07 | 6.66829 |
| CRISPLD1 | 1.076443 | 5.754705 | 5.411261 | 1.99E-07 | 7.77E-07 | 6.565274 |
| FCGR3A /// FCGR3B | 1.195838 | 6.529122 | 5.410488 | 2.00E-07 | 7.79E-07 | 6.561696 |
| FGL1 | -1.72791 | 7.886658 | -5.39438 | 2.16E-07 | 8.34E-07 | 6.487306 |
| EREG | 1.200051 | 4.56658 | 5.390211 | 2.20E-07 | 8.49E-07 | 6.468056 |
| CLDN11 | 1.287636 | 7.086229 | 5.383551 | 2.27E-07 | 8.74E-07 | 6.437351 |
| SYBU | -1.20239 | 7.814382 | -5.38034 | 2.31E-07 | 8.85E-07 | 6.422543 |
| PDZK1 | -1.23378 | 5.55211 | -5.37707 | 2.34E-07 | 8.98E-07 | 6.407502 |
| WDR72 | 1.388289 | 6.990221 | 5.350426 | 2.66E-07 | 1.00E-06 | 6.285057 |
| SPRR1B | 1.192222 | 4.830513 | 5.330875 | 2.92E-07 | 1.09E-06 | 6.195486 |
| F2RL2 | 1.224454 | 6.160824 | 5.320458 | 3.07E-07 | 1.13E-06 | 6.147859 |
| SLC26A9 | 1.114337 | 5.811886 | 5.305947 | 3.29E-07 | 1.21E-06 | 6.081624 |
| TFF2 | 1.790005 | 8.541276 | 5.302912 | 3.34E-07 | 1.22E-06 | 6.067789 |
| STMN2 | 1.071113 | 6.384787 | 5.30197 | 3.35E-07 | 1.23E-06 | 6.063498 |
| AK021804 | 1.002756 | 8.478597 | 5.2933 | 3.49E-07 | 1.27E-06 | 6.024008 |
| IGFBP2 | -1.1286 | 8.495912 | -5.27958 | 3.73E-07 | 1.34E-06 | 5.961607 |
| GATM | -1.49011 | 10.19602 | -5.27835 | 3.75E-07 | 1.35E-06 | 5.956006 |
| AKR1C3 | 1.020789 | 9.125185 | 5.240132 | 4.49E-07 | 1.59E-06 | 5.782874 |
| PAK3 | -1.01738 | 5.372135 | -5.19932 | 5.43E-07 | 1.88E-06 | 5.598976 |
| EPYC | 1.473051 | 4.467381 | 5.174965 | 6.08E-07 | 2.08E-06 | 5.489756 |
| BCL2A1 | 1.216322 | 6.651682 | 5.153199 | 6.73E-07 | 2.28E-06 | 5.392456 |
| SLC2A3 | 1.126119 | 8.107716 | 5.137494 | 7.24E-07 | 2.43E-06 | 5.322435 |
| NR5A2 | -1.47961 | 5.934374 | -5.12635 | 7.62E-07 | 2.54E-06 | 5.272832 |
| TDO2 | 1.145924 | 6.127274 | 5.08544 | 9.21E-07 | 3.01E-06 | 5.091496 |
| NRCAM | -1.04976 | 7.413266 | -5.06217 | 1.02E-06 | 3.32E-06 | 4.988833 |
| LYZ | 1.582198 | 9.11388 | 5.061915 | 1.03E-06 | 3.32E-06 | 4.987696 |
| GSTA1 | -1.45547 | 8.402783 | -5.03817 | 1.14E-06 | 3.67E-06 | 4.883283 |
| IGHG1 /// IGHG2 /// IGHM /// IGHV4-31 /// MIR8071-1 /// MIR8071-2 | 1.742658 | 11.48965 | 5.031153 | 1.18E-06 | 3.77E-06 | 4.852512 |
| LINC00261 | -1.01177 | 6.81392 | -5.02272 | 1.23E-06 | 3.90E-06 | 4.815569 |
| HOMER2 | -1.11265 | 5.652432 | -4.99293 | 1.41E-06 | 4.41E-06 | 4.685393 |
| GIMAP2 | 1.027209 | 7.13342 | 4.986414 | 1.45E-06 | 4.52E-06 | 4.657008 |
| RGS1 | 1.157754 | 8.107844 | 4.947859 | 1.72E-06 | 5.29E-06 | 4.489587 |
| NTRK2 | -1.14315 | 6.375895 | -4.93761 | 1.81E-06 | 5.51E-06 | 4.445229 |
| NPY1R | -1.07228 | 5.516855 | -4.93061 | 1.86E-06 | 5.67E-06 | 4.415014 |
| EPB41L4B | -1.19372 | 9.205699 | -4.87282 | 2.42E-06 | 7.15E-06 | 4.166568 |
| REG4 | 1.950293 | 6.295979 | 4.867858 | 2.47E-06 | 7.29E-06 | 4.145329 |
| RNF128 | 1.083606 | 8.847371 | 4.835034 | 2.86E-06 | 8.31E-06 | 4.00532 |
| PTGS2 | 1.288225 | 6.89051 | 4.830593 | 2.92E-06 | 8.45E-06 | 3.986435 |
| PTGIS | 1.008048 | 8.494769 | 4.816111 | 3.11E-06 | 8.96E-06 | 3.924938 |
| SPRR3 | 1.084393 | 6.086829 | 4.788899 | 3.51E-06 | 9.98E-06 | 3.809765 |
| IMPA2 | -1.07376 | 8.778966 | -4.74653 | 4.22E-06 | 1.18E-05 | 3.631464 |
| SYCN | -2.65187 | 8.818697 | -4.71747 | 4.80E-06 | 1.32E-05 | 3.509866 |
| CHI3L1 | 1.100489 | 6.814337 | 4.714643 | 4.86E-06 | 1.33E-05 | 3.498051 |
| CELA2B | -2.47015 | 8.682682 | -4.70667 | 5.03E-06 | 1.38E-05 | 3.464804 |
| LY96 | 1.0534 | 7.807321 | 4.672041 | 5.85E-06 | 1.58E-05 | 3.320924 |
| EPCAM | 1.047015 | 10.95299 | 4.630786 | 6.99E-06 | 1.86E-05 | 3.150594 |
| KCNJ16 | -1.54889 | 7.10724 | -4.62702 | 7.10E-06 | 1.88E-05 | 3.135108 |
| CYP1B1 | 1.261638 | 8.357881 | 4.621003 | 7.29E-06 | 1.93E-05 | 3.110374 |
| CELP | -2.34157 | 9.094537 | -4.57205 | 8.99E-06 | 2.34E-05 | 2.910139 |
| IAPP | -1.74789 | 7.274786 | -4.53356 | 1.06E-05 | 2.71E-05 | 2.753882 |
| CLRN3 | 1.253454 | 6.061567 | 4.521052 | 1.12E-05 | 2.84E-05 | 2.703316 |
| AKR1B10 | 1.398677 | 7.445217 | 4.51543 | 1.14E-05 | 2.90E-05 | 2.68063 |
| PNLIPRP2 | -2.48163 | 9.247911 | -4.48104 | 1.32E-05 | 3.30E-05 | 2.542326 |
| DMBT1 | 1.232904 | 8.07322 | 4.472248 | 1.37E-05 | 3.42E-05 | 2.507121 |
| DPT | -1.00548 | 7.227513 | -4.45485 | 1.48E-05 | 3.64E-05 | 2.437599 |
| PNLIPRP1 | -2.3974 | 8.926813 | -4.45384 | 1.48E-05 | 3.66E-05 | 2.433577 |
| TMEM97 | -1.14041 | 9.123185 | -4.41058 | 1.78E-05 | 4.31E-05 | 2.261642 |
| CUZD1 | -2.25308 | 8.840132 | -4.35684 | 2.22E-05 | 5.28E-05 | 2.049936 |
| SPRR1A | 1.112957 | 4.742903 | 4.249183 | 3.45E-05 | 7.87E-05 | 1.63219 |
| HPGD | 1.063668 | 6.389788 | 4.245995 | 3.49E-05 | 7.96E-05 | 1.619949 |
| BEX1 | -1.11386 | 8.51342 | -4.23001 | 3.73E-05 | 8.44E-05 | 1.558678 |
| HSD17B2 | 1.062847 | 8.17011 | 4.22646 | 3.78E-05 | 8.55E-05 | 1.545102 |
| CPA2 | -2.2277 | 9.869941 | -4.19382 | 4.31E-05 | 9.64E-05 | 1.420658 |
| IGLV1-44 | 1.122429 | 10.84332 | 4.079638 | 6.79E-05 | 0.000146 | 0.99163 |
| PRSS3 | -1.63337 | 10.50719 | -4.02718 | 8.33E-05 | 0.000176 | 0.797801 |
| GP2 | -2.08512 | 9.485438 | -3.994 | 9.48E-05 | 0.000198 | 0.676269 |
| ADH1B | -1.1076 | 8.28646 | -3.9269 | 0.000123 | 0.000251 | 0.43312 |
| CTRC | -2.08121 | 9.85044 | -3.8992 | 0.000136 | 0.000277 | 0.333723 |
| CLPS | -2.17642 | 9.882772 | -3.8703 | 0.000152 | 0.000306 | 0.230671 |
| AZGP1 | -1.21455 | 8.066667 | -3.86006 | 0.000158 | 0.000318 | 0.194324 |
| CD52 | 1.014349 | 7.617846 | 3.833228 | 0.000175 | 0.000349 | 0.099427 |
| IGLC1 | 1.103378 | 12.01476 | 3.771881 | 0.00022 | 0.00043 | -0.11541 |
| FAM3B | -1.10375 | 8.898585 | -3.76109 | 0.000229 | 0.000447 | -0.15287 |
| DLK1 | -1.08613 | 6.693624 | -3.74149 | 0.000246 | 0.000478 | -0.22073 |
| CEL | -2.13741 | 9.772746 | -3.7124 | 0.000274 | 0.000527 | -0.3209 |
| ACTG1P4 /// AMY1A /// AMY1B /// AMY1C /// AMY2A /// AMY2B | -1.64561 | 10.73348 | -3.66341 | 0.000328 | 0.000621 | -0.48801 |
| CELA2A /// CELA2B | -2.15761 | 9.549331 | -3.55426 | 0.000485 | 0.000891 | -0.85351 |
| PLA2G1B | -2.15079 | 9.698225 | -3.5349 | 0.000519 | 0.000949 | -0.91737 |
| PRSS3P2 | -1.37223 | 11.11034 | -3.52884 | 0.000531 | 0.000968 | -0.93729 |
| CRP | 1.065062 | 5.261867 | 3.48759 | 0.000613 | 0.001107 | -1.07206 |
| PTX3 | -1.00632 | 5.598113 | -3.48687 | 0.000615 | 0.00111 | -1.0744 |
| CELA3B | -1.80996 | 10.26293 | -3.36951 | 0.000922 | 0.001612 | -1.45024 |
| CELA3A | -1.82029 | 10.26085 | -3.26001 | 0.001334 | 0.002264 | -1.79069 |
| CPA1 | -2.00666 | 9.398569 | -3.25142 | 0.001373 | 0.002323 | -1.81697 |
| SLC4A4 | -1.0548 | 9.784514 | -3.18691 | 0.001697 | 0.002826 | -2.0124 |
| REG1P | -1.01758 | 6.718114 | -3.12566 | 0.002071 | 0.003399 | -2.19474 |
| PRSS2 | -1.36852 | 11.11387 | -3.07894 | 0.002405 | 0.003905 | -2.33167 |
| SLC3A1 | -1.07359 | 9.199477 | -3.01431 | 0.00295 | 0.00472 | -2.51805 |
| CTRB1 /// CTRB2 | -1.71914 | 10.52306 | -2.97746 | 0.00331 | 0.005255 | -2.62273 |
| REG1A | -1.36704 | 11.32062 | -2.97661 | 0.003318 | 0.005267 | -2.62511 |
| PNLIP | -1.66728 | 10.56415 | -2.83875 | 0.005054 | 0.007784 | -3.00625 |
| CTRB2 | -1.46776 | 10.90058 | -2.8219 | 0.005315 | 0.008153 | -3.05169 |
| PRSS1 /// PRSS2 | -1.46859 | 10.67876 | -2.73634 | 0.00684 | 0.010287 | -3.27867 |
| CPB1 | -1.48541 | 10.8936 | -2.6343 | 0.009171 | 0.013496 | -3.54095 |
| REG1B | -1.41303 | 9.97555 | -2.57169 | 0.010933 | 0.015878 | -3.6973 |

Table S3: Risk score of each PAAD sample (TCGA-PAAD).

|  | | |
| --- | --- | --- |
| **TCGA-PAAD Samples** | **Risk score** | **Group** |
| TCGA_3A_A9IS_01 | 11.70591 | Low |
| TCGA_3A_A9IO_01 | 12.70588 | Low |
| TCGA_F2_6880_01 | 13.16263 | Low |
| TCGA_3A_A9IV_01 | 13.96503 | Low |
| TCGA_3A_A9IN_01 | 13.98951 | Low |
| TCGA_3A_A9IL_01 | 14.06121 | Low |
| TCGA_3A_A9IR_01 | 14.42718 | Low |
| TCGA_3A_A9IJ_01 | 14.85648 | Low |
| TCGA_2L_AAQM_01 | 14.89851 | Low |
| TCGA_IB_8126_01 | 15.42722 | Low |
| TCGA_F2_A44H_01 | 15.81131 | Low |
| TCGA_IB_AAUM_01 | 16.05112 | Low |
| TCGA_HZ_8003_01 | 16.05689 | Low |
| TCGA_2J_AABV_01 | 16.10894 | Low |
| TCGA_2J_AABT_01 | 16.23374 | Low |
| TCGA_FB_A4P6_01 | 16.28344 | Low |
| TCGA_2J_AABP_01 | 16.36617 | Low |
| TCGA_IB_7897_01 | 16.51051 | Low |
| TCGA_IB_AAUT_01 | 16.51741 | Low |
| TCGA_Z5_AAPL_01 | 16.73473 | Low |
| TCGA_2J_AAB9_01 | 16.84498 | Low |
| TCGA_XD_AAUG_01 | 16.87434 | Low |
| TCGA_HZ_A49H_01 | 16.91727 | Low |
| TCGA_IB_7649_01 | 16.93817 | Low |
| TCGA_FB_A4P5_01 | 16.96964 | Low |
| TCGA_LB_A9Q5_01 | 17.03216 | Low |
| TCGA_IB_AAUV_01 | 17.04894 | Low |
| TCGA_2J_AABR_01 | 17.08383 | Low |
| TCGA_XD_AAUH_01 | 17.08765 | Low |
| TCGA_US_A77J_01 | 17.10565 | Low |
| TCGA_HZ_7923_01 | 17.12737 | Low |
| TCGA_HZ_8002_01 | 17.15271 | Low |
| TCGA_XN_A8T5_01 | 17.17362 | Low |
| TCGA_HZ_8637_01 | 17.2452 | Low |
| TCGA_HZ_7920_01 | 17.25898 | Low |
| TCGA_HZ_7918_01 | 17.27213 | Low |
| TCGA_HZ_A49G_01 | 17.29006 | Low |
| TCGA_RL_AAAS_01 | 17.2916 | Low |
| TCGA_H6_A45N_01 | 17.29891 | Low |
| TCGA_3A_A9IX_01 | 17.34073 | Low |
| TCGA_2J_AABA_01 | 17.37043 | Low |
| TCGA_IB_AAUW_01 | 17.38691 | Low |
| TCGA_Q3_A5QY_01 | 17.39481 | Low |
| TCGA_IB_7891_01 | 17.43396 | Low |
| TCGA_IB_AAUS_01 | 17.46655 | Low |
| TCGA_H8_A6C1_01 | 17.4676 | Low |
| TCGA_F2_7276_01 | 17.47052 | Low |
| TCGA_IB_7888_01 | 17.47353 | Low |
| TCGA_IB_AAUR_01 | 17.48306 | Low |
| TCGA_FB_AAPQ_01 | 17.49409 | Low |
| TCGA_HV_A5A5_01 | 17.5235 | Low |
| TCGA_F2_7273_01 | 17.52809 | Low |
| TCGA_HZ_A77P_01 | 17.56029 | Low |
| TCGA_HZ_A77Q_01 | 17.56649 | Low |
| TCGA_FB_AAPZ_01 | 17.61851 | Low |
| TCGA_IB_A6UG_01 | 17.64017 | Low |
| TCGA_IB_AAUP_01 | 17.659 | Low |
| TCGA_FB_AAPP_01 | 17.69879 | Low |
| TCGA_FB_AAQ6_01 | 17.71684 | Low |
| TCGA_FB_AAPS_01 | 17.7489 | Low |
| TCGA_F2_A7TX_01 | 17.74965 | Low |
| TCGA_HV_A5A3_01 | 17.75362 | Low |
| TCGA_HZ_8519_01 | 17.78117 | Low |
| TCGA_2J_AAB8_01 | 17.78234 | Low |
| TCGA_2J_AABU_01 | 17.81981 | Low |
| TCGA_2J_AABO_01 | 17.83738 | Low |
| TCGA_HZ_A4BH_01 | 17.86214 | Low |
| TCGA_3A_A9I9_01 | 17.86562 | Low |
| TCGA_IB_A5ST_01 | 17.86921 | Low |
| TCGA_S4_A8RP_01 | 17.87202 | Low |
| TCGA_HZ_8317_01 | 17.94579 | Low |
| TCGA_IB_7645_01 | 17.97133 | Low |
| TCGA_FB_A545_01 | 17.97466 | Low |
| TCGA_3A_A9I5_01 | 18.00082 | Low |
| TCGA_3A_A9IZ_01 | 18.04292 | Low |
| TCGA_XN_A8T3_01 | 18.04377 | Low |
| TCGA_FB_A7DR_01 | 18.05667 | Low |
| TCGA_IB_7890_01 | 18.12221 | Low |
| TCGA_HZ_A4BK_01 | 18.17422 | Low |
| TCGA_3E_AAAY_01 | 18.17671 | Low |
| TCGA_Q3_AA2A_01 | 18.18049 | Low |
| TCGA_IB_7652_01 | 18.20613 | Low |
| TCGA_HZ_A49I_01 | 18.21005 | Low |
| TCGA_HV_A7OP_01 | 18.22325 | Low |
| TCGA_IB_7646_01 | 18.23633 | Low |
| TCGA_US_A776_01 | 18.25623 | Low |
| TCGA_HZ_8005_01 | 18.26157 | Low |
| TCGA_HZ_7922_01 | 18.26186 | Low |
| TCGA_HZ_8315_01 | 18.26572 | Low |
| TCGA_HV_A5A4_01 | 18.29518 | High |
| TCGA_US_A77E_01 | 18.32434 | High |
| TCGA_IB_A7M4_01 | 18.32916 | High |
| TCGA_S4_A8RM_01 | 18.33885 | High |
| TCGA_RB_AA9M_01 | 18.33979 | High |
| TCGA_2J_AABE_01 | 18.3416 | High |
| TCGA_YB_A89D_01 | 18.34956 | High |
| TCGA_F2_A44G_01 | 18.37606 | High |
| TCGA_XD_AAUI_01 | 18.38187 | High |
| TCGA_IB_A5SP_01 | 18.41645 | High |
| TCGA_IB_7886_01 | 18.43196 | High |
| TCGA_HZ_A8P0_01 | 18.44322 | High |
| TCGA_HZ_8638_01 | 18.44606 | High |
| TCGA_US_A774_01 | 18.46685 | High |
| TCGA_HZ_A8P1_01 | 18.46768 | High |
| TCGA_HV_AA8V_01 | 18.48279 | High |
| TCGA_IB_7654_01 | 18.48513 | High |
| TCGA_IB_AAUQ_01 | 18.49558 | High |
| TCGA_M8_A5N4_01 | 18.5324 | High |
| TCGA_L1_A7W4_01 | 18.54412 | High |
| TCGA_US_A77G_01 | 18.55312 | High |
| TCGA_IB_7651_01 | 18.55788 | High |
| TCGA_F2_A8YN_01 | 18.55969 | High |
| TCGA_FB_AAPY_01 | 18.57195 | High |
| TCGA_HZ_8001_01 | 18.57259 | High |
| TCGA_2L_AAQE_01 | 18.57409 | High |
| TCGA_HZ_7925_01 | 18.60328 | High |
| TCGA_2J_AABF_01 | 18.61401 | High |
| TCGA_LB_A7SX_01 | 18.62707 | High |
| TCGA_IB_7887_01 | 18.6431 | High |
| TCGA_IB_7644_01 | 18.66226 | High |
| TCGA_FB_AAQ3_01 | 18.6716 | High |
| TCGA_2J_AAB4_01 | 18.73005 | High |
| TCGA_HZ_7919_01 | 18.76531 | High |
| TCGA_IB_AAUN_01 | 18.76747 | High |
| TCGA_2J_AABI_01 | 18.7821 | High |
| TCGA_IB_A5SQ_01 | 18.78917 | High |
| TCGA_HV_A5A6_01 | 18.79809 | High |
| TCGA_3A_A9I7_01 | 18.81524 | High |
| TCGA_IB_A5SS_01 | 18.81639 | High |
| TCGA_FB_AAQ0_01 | 18.82506 | High |
| TCGA_2L_AAQI_01 | 18.82725 | High |
| TCGA_IB_A5SO_01 | 18.84826 | High |
| TCGA_HZ_7924_01 | 18.88607 | High |
| TCGA_IB_AAUO_01 | 18.9063 | High |
| TCGA_FB_AAQ1_01 | 18.91172 | High |
| TCGA_FB_A78T_01 | 18.92499 | High |
| TCGA_FB_A5VM_01 | 18.93772 | High |
| TCGA_HZ_7289_01 | 18.94826 | High |
| TCGA_3E_AAAZ_01 | 19.01548 | High |
| TCGA_2L_AAQL_01 | 19.04868 | High |
| TCGA_YY_A8LH_01 | 19.05598 | High |
| TCGA_IB_7889_01 | 19.08846 | High |
| TCGA_HV_AA8X_01 | 19.0908 | High |
| TCGA_XD_AAUL_01 | 19.11035 | High |
| TCGA_3A_A9IB_01 | 19.12777 | High |
| TCGA_LB_A8F3_01 | 19.13653 | High |
| TCGA_RB_A7B8_01 | 19.15498 | High |
| TCGA_3A_A9IC_01 | 19.16206 | High |
| TCGA_2J_AAB1_01 | 19.16379 | High |
| TCGA_US_A779_01 | 19.17803 | High |
| TCGA_IB_A6UF_01 | 19.19492 | High |
| TCGA_IB_A7LX_01 | 19.19704 | High |
| TCGA_3A_A9IU_01 | 19.21721 | High |
| TCGA_HZ_A77O_01 | 19.22254 | High |
| TCGA_3A_A9J0_01 | 19.26238 | High |
| TCGA_HZ_A9TJ_01 | 19.26812 | High |
| TCGA_2L_AAQA_01 | 19.27717 | High |
| TCGA_2J_AABK_01 | 19.28708 | High |
| TCGA_IB_7893_01 | 19.30519 | High |
| TCGA_IB_AAUU_01 | 19.34057 | High |
| TCGA_S4_A8RO_01 | 19.38972 | High |
| TCGA_HV_A7OL_01 | 19.3926 | High |
| TCGA_H6_8124_01 | 19.41116 | High |
| TCGA_YH_A8SY_01 | 19.44128 | High |
| TCGA_HZ_8636_01 | 19.46138 | High |
| TCGA_PZ_A5RE_01 | 19.50735 | High |
| TCGA_OE_A75W_01 | 19.53987 | High |
| TCGA_IB_8127_01 | 19.55615 | High |
| TCGA_2L_AAQJ_01 | 19.56825 | High |
| TCGA_HZ_7926_01 | 19.60587 | High |
| TCGA_F2_6879_01 | 19.64361 | High |
| TCGA_IB_7885_01 | 19.72019 | High |
| TCGA_3A_A9IH_01 | 19.74842 | High |
| TCGA_2J_AAB6_01 | 19.82595 | High |
| TCGA_FB_AAPU_01 | 19.91688 | High |
| TCGA_FB_AAQ2_01 | 20.06377 | High |
| TCGA_2J_AABH_01 | 20.10768 | High |

Table S4: Risk score of each PAAD sample (MMSD).

|  | | |
| --- | --- | --- |
| **MMSD Samples** | **Risk score** | **Group** |
| GSM536948 | 4.66362 | Low |
| GSM536922 | 4.771166 | Low |
| GSM711938 | 4.837692 | Low |
| GSM1844205 | 4.87391 | Low |
| GSM711984 | 5.104347 | Low |
| GSM1844177 | 5.213428 | Low |
| GSM1844175 | 5.217452 | Low |
| GSM536462 | 5.277852 | Low |
| GSM1844249 | 5.317304 | Low |
| GSM1844215 | 5.445367 | Low |
| GSM536459 | 5.567085 | Low |
| GSM1844212 | 5.642919 | Low |
| GSM536479 | 5.643929 | Low |
| GSM1844232 | 5.653089 | Low |
| GSM536455 | 5.732335 | Low |
| GSM711936 | 5.749453 | Low |
| GSM1844120 | 5.755473 | Low |
| GSM1844165 | 5.774041 | Low |
| GSM1844208 | 5.777734 | Low |
| GSM536897 | 5.814776 | Low |
| GSM536940 | 5.846817 | Low |
| GSM1844114 | 5.9408 | Low |
| GSM536465 | 6.00603 | Low |
| GSM711946 | 6.023699 | Low |
| GSM1844138 | 6.062058 | Low |
| GSM536488 | 6.08676 | Low |
| GSM1844174 | 6.150943 | Low |
| GSM711910 | 6.158298 | Low |
| GSM536946 | 6.17253 | Low |
| GSM1844218 | 6.283521 | Low |
| GSM711944 | 6.320682 | Low |
| GSM536934 | 6.325237 | Low |
| GSM536918 | 6.343198 | Low |
| GSM536926 | 6.355995 | Low |
| GSM536486 | 6.378368 | Low |
| GSM1844136 | 6.382454 | Low |
| GSM536941 | 6.412868 | Low |
| GSM536904 | 6.41855 | Low |
| GSM1844239 | 6.42887 | Low |
| GSM536942 | 6.507686 | Low |
| GSM1844241 | 6.5178 | Low |
| GSM1844161 | 6.532599 | Low |
| GSM1844152 | 6.539513 | Low |
| GSM536487 | 6.542564 | Low |
| GSM1844203 | 6.557681 | Low |
| GSM536895 | 6.56906 | Low |
| GSM1844240 | 6.593642 | Low |
| GSM711990 | 6.632668 | Low |
| GSM536473 | 6.636308 | Low |
| GSM711974 | 6.662336 | Low |
| GSM536490 | 6.671446 | Low |
| GSM1844122 | 6.693844 | Low |
| GSM1844171 | 6.704486 | Low |
| GSM1844191 | 6.726041 | Low |
| GSM536481 | 6.731959 | Low |
| GSM1844226 | 6.755994 | Low |
| GSM536476 | 6.773095 | Low |
| GSM1844143 | 6.778253 | Low |
| GSM536463 | 6.78751 | Low |
| GSM1844234 | 6.788979 | Low |
| GSM711932 | 6.795459 | Low |
| GSM711978 | 6.82197 | Low |
| GSM711926 | 6.870689 | Low |
| GSM1844119 | 6.87961 | Low |
| GSM1844216 | 6.889789 | Low |
| GSM536945 | 6.926816 | Low |
| GSM1844198 | 6.949016 | Low |
| GSM536464 | 6.957476 | Low |
| GSM1844168 | 6.967488 | Low |
| GSM1844202 | 6.967972 | Low |
| GSM1844235 | 6.987701 | Low |
| GSM1844199 | 6.998892 | Low |
| GSM1844227 | 7.013176 | Low |
| GSM711924 | 7.014962 | Low |
| GSM536489 | 7.020523 | Low |
| GSM1844141 | 7.022126 | Low |
| GSM536492 | 7.038917 | Low |
| GSM1844158 | 7.057293 | Low |
| GSM711992 | 7.061661 | Low |
| GSM536461 | 7.087372 | Low |
| GSM536923 | 7.089496 | Low |
| GSM1844201 | 7.104519 | Low |
| GSM536482 | 7.108973 | Low |
| GSM1844121 | 7.114769 | Low |
| GSM1844192 | 7.116285 | Low |
| GSM536944 | 7.11777 | Low |
| GSM1844187 | 7.118801 | Low |
| GSM536938 | 7.124212 | Low |
| GSM1844217 | 7.13342 | Low |
| GSM536935 | 7.137415 | Low |
| GSM536882 | 7.140828 | Low |
| GSM536467 | 7.156548 | Low |
| GSM536893 | 7.159288 | Low |
| GSM536902 | 7.177561 | Low |
| GSM1844229 | 7.185835 | Low |
| GSM711954 | 7.188574 | Low |
| GSM1844222 | 7.189214 | Low |
| GSM536474 | 7.197145 | Low |
| GSM711950 | 7.197247 | Low |
| GSM1844135 | 7.197611 | Low |
| GSM1844123 | 7.208323 | Low |
| GSM711958 | 7.209764 | Low |
| GSM1844220 | 7.217691 | Low |
| GSM1844214 | 7.221016 | Low |
| GSM711948 | 7.223963 | Low |
| GSM711970 | 7.224735 | Low |
| GSM536899 | 7.235181 | Low |
| GSM1844140 | 7.239378 | Low |
| GSM1844167 | 7.247066 | Low |
| GSM536894 | 7.25869 | Low |
| GSM1844149 | 7.26032 | Low |
| GSM1844170 | 7.271119 | Low |
| GSM1844124 | 7.282086 | Low |
| GSM711980 | 7.285132 | Low |
| GSM536891 | 7.292184 | Low |
| GSM536469 | 7.309458 | Low |
| GSM1844196 | 7.314782 | Low |
| GSM536468 | 7.32061 | Low |
| GSM536910 | 7.346827 | Low |
| GSM536456 | 7.355815 | Low |
| GSM711930 | 7.370807 | Low |
| GSM1844157 | 7.379847 | Low |
| GSM1844164 | 7.381706 | Low |
| GSM1844211 | 7.387356 | Low |
| GSM536939 | 7.390892 | Low |
| GSM1844185 | 7.395984 | Low |
| GSM536484 | 7.400363 | Low |
| GSM711922 | 7.401516 | Low |
| GSM711940 | 7.404845 | Low |
| GSM711934 | 7.405394 | Low |
| GSM536937 | 7.419645 | Low |
| GSM711960 | 7.426303 | Low |
| GSM536928 | 7.437147 | Low |
| GSM1844169 | 7.437468 | Low |
| GSM536483 | 7.438161 | Low |
| GSM536907 | 7.441242 | High |
| GSM1844172 | 7.441504 | High |
| GSM536454 | 7.456208 | High |
| GSM1844117 | 7.461174 | High |
| GSM1844134 | 7.468704 | High |
| GSM1844194 | 7.469965 | High |
| GSM1844155 | 7.47187 | High |
| GSM536900 | 7.477306 | High |
| GSM1844207 | 7.483209 | High |
| GSM1844209 | 7.484057 | High |
| GSM536932 | 7.486257 | High |
| GSM1844179 | 7.501783 | High |
| GSM536949 | 7.502429 | High |
| GSM536906 | 7.50331 | High |
| GSM711928 | 7.505882 | High |
| GSM1844237 | 7.514248 | High |
| GSM536947 | 7.515698 | High |
| GSM1844163 | 7.519963 | High |
| GSM1844178 | 7.538257 | High |
| GSM536885 | 7.542071 | High |
| GSM1844115 | 7.547138 | High |
| GSM536460 | 7.550038 | High |
| GSM536883 | 7.559856 | High |
| GSM1844150 | 7.564491 | High |
| GSM536903 | 7.565705 | High |
| GSM1844213 | 7.570318 | High |
| GSM536472 | 7.583397 | High |
| GSM536929 | 7.585693 | High |
| GSM536898 | 7.594864 | High |
| GSM1844236 | 7.599339 | High |
| GSM536889 | 7.602272 | High |
| GSM536458 | 7.610153 | High |
| GSM1844221 | 7.625698 | High |
| GSM1844142 | 7.628149 | High |
| GSM1844223 | 7.633089 | High |
| GSM1844180 | 7.635148 | High |
| GSM1844145 | 7.638184 | High |
| GSM536475 | 7.641626 | High |
| GSM1844228 | 7.65184 | High |
| GSM1844166 | 7.653277 | High |
| GSM1844181 | 7.65825 | High |
| GSM1844133 | 7.683826 | High |
| GSM1844204 | 7.692261 | High |
| GSM711904 | 7.699009 | High |
| GSM1844225 | 7.703128 | High |
| GSM1844125 | 7.711462 | High |
| GSM711976 | 7.714907 | High |
| GSM536892 | 7.717221 | High |
| GSM711968 | 7.728336 | High |
| GSM1844112 | 7.735475 | High |
| GSM536931 | 7.743588 | High |
| GSM711942 | 7.750476 | High |
| GSM1844206 | 7.75495 | High |
| GSM1844200 | 7.755031 | High |
| GSM1844233 | 7.757563 | High |
| GSM711956 | 7.763224 | High |
| GSM1844230 | 7.771766 | High |
| GSM536911 | 7.784849 | High |
| GSM536930 | 7.792549 | High |
| GSM1844182 | 7.79496 | High |
| GSM711916 | 7.795753 | High |
| GSM536943 | 7.79903 | High |
| GSM1844210 | 7.803452 | High |
| GSM536933 | 7.809531 | High |
| GSM711988 | 7.823766 | High |
| GSM536477 | 7.831666 | High |
| GSM536470 | 7.834408 | High |
| GSM536936 | 7.838358 | High |
| GSM1844189 | 7.83991 | High |
| GSM711914 | 7.857385 | High |
| GSM1844146 | 7.87569 | High |
| GSM1844186 | 7.883851 | High |
| GSM536888 | 7.890231 | High |
| GSM536466 | 7.894896 | High |
| GSM711982 | 7.922945 | High |
| GSM536457 | 7.930942 | High |
| GSM1844128 | 7.931383 | High |
| GSM536896 | 7.972235 | High |
| GSM1844162 | 7.974628 | High |
| GSM1844139 | 7.975925 | High |
| GSM1844113 | 7.976944 | High |
| GSM711966 | 8.000156 | High |
| GSM1844190 | 8.010319 | High |
| GSM1844153 | 8.0309 | High |
| GSM1844131 | 8.031308 | High |
| GSM1844148 | 8.04482 | High |
| GSM1844116 | 8.058983 | High |
| GSM1844193 | 8.066336 | High |
| GSM536890 | 8.078413 | High |
| GSM536919 | 8.078754 | High |
| GSM1844137 | 8.08478 | High |
| GSM1844144 | 8.085719 | High |
| GSM1844154 | 8.094889 | High |
| GSM1844132 | 8.095588 | High |
| GSM1844231 | 8.103822 | High |
| GSM536480 | 8.111655 | High |
| GSM1844224 | 8.11374 | High |
| GSM536909 | 8.117934 | High |
| GSM536927 | 8.133237 | High |
| GSM1844160 | 8.14668 | High |
| GSM1844159 | 8.167762 | High |
| GSM1844151 | 8.173542 | High |
| GSM1844118 | 8.189009 | High |
| GSM1844188 | 8.195695 | High |
| GSM1844238 | 8.217182 | High |
| GSM711962 | 8.24222 | High |
| GSM536924 | 8.243114 | High |
| GSM536920 | 8.253504 | High |
| GSM1844173 | 8.312108 | High |
| GSM1844130 | 8.31394 | High |
| GSM711986 | 8.31715 | High |
| GSM536925 | 8.317867 | High |
| GSM1844129 | 8.327208 | High |
| GSM536471 | 8.344226 | High |
| GSM536887 | 8.351944 | High |
| GSM711972 | 8.353225 | High |
| GSM711952 | 8.383257 | High |
| GSM1844126 | 8.405052 | High |
| GSM536921 | 8.413476 | High |
| GSM1844219 | 8.415733 | High |
| GSM536886 | 8.429897 | High |
| GSM536917 | 8.43811 | High |
| GSM536884 | 8.439896 | High |
| GSM536905 | 8.440122 | High |
| GSM711964 | 8.48265 | High |
| GSM711908 | 8.508912 | High |
| GSM1844156 | 8.521074 | High |
| GSM536485 | 8.534157 | High |
| GSM536478 | 8.545299 | High |
| GSM536901 | 8.581262 | High |
| GSM711906 | 8.750848 | High |
| GSM1844147 | 8.778169 | High |
| GSM536908 | 8.841088 | High |
| GSM536491 | 9.060924 | High |

Table S5: Risk score of each PAAD sample (IMvigor210).

| **IMvigor210 Samples** | **Risk score** | **Group** |
| --- | --- | --- |
| SAMb8101c538753 | 16.28084 | High |
| SAM31d9176e11fb | 15.85463 | High |
| SAM30b5c6c54cf7 | 15.70921 | High |
| SAM7bff231634e9 | 15.653 | High |
| SAM1a87df750b9d | 15.5797 | High |
| SAM2b672f4336c7 | 15.55215 | High |
| SAMce39dd79b441 | 15.45448 | High |
| SAMd1bd63734394 | 15.3593 | High |
| SAM09c84ec0cf34 | 15.26779 | High |
| SAM6964a6d7b967 | 15.23943 | High |
| SAMa321770ac31c | 15.20689 | High |
| SAMb3c02294aba7 | 15.13271 | High |
| SAMe41b1e773582 | 15.12393 | High |
| SAM978a587b207e | 15.11612 | High |
| SAM7829a341b9f3 | 15.10333 | High |
| SAM670649e105b5 | 15.09636 | High |
| SAM5767dd75d142 | 15.07832 | High |
| SAM6662f5181f87 | 15.06208 | High |
| SAM4501e41e4751 | 15.05212 | High |
| SAMe5bc41772bc9 | 15.04817 | High |
| SAMaf42c1541269 | 15.02463 | High |
| SAM468a9e1dc821 | 14.98182 | High |
| SAMe7e4f7c076a7 | 14.98076 | High |
| SAMb2f1d0e54ece | 14.97914 | High |
| SAM5a2347c0498a | 14.92777 | High |
| SAM08cce2fa88f2 | 14.91009 | High |
| SAMa90d73f8d891 | 14.90695 | High |
| SAM110501d0eedb | 14.90373 | High |
| SAMa1871f491b02 | 14.9025 | High |
| SAM187e056d6a2a | 14.89759 | High |
| SAM25510f300d79 | 14.89469 | High |
| SAM39eb94fa504d | 14.88724 | High |
| SAM7114d99032ec | 14.88487 | High |
| SAM7538ad9ff524 | 14.88075 | High |
| SAMe3210d3632b4 | 14.87657 | High |
| SAM3a1c9632ff7b | 14.86578 | High |
| SAMcabb6d58ff55 | 14.82822 | High |
| SAM1ac4e3dee297 | 14.82552 | High |
| SAMb15ad09d6e24 | 14.82281 | High |
| SAM943df5cf15df | 14.7961 | High |
| SAM9e11ec6bea80 | 14.79268 | High |
| SAMd98bac0a070f | 14.78457 | High |
| SAM94859b440b1d | 14.78148 | High |
| SAMb4c7a001537d | 14.77361 | High |
| SAM12502d970c10 | 14.76173 | High |
| SAMda4d892fddc8 | 14.74681 | High |
| SAM36851bc8b9ae | 14.7408 | High |
| SAM6cbc10abddb0 | 14.72422 | High |
| SAM45c8e6412c66 | 14.71802 | High |
| SAM3cb94b0d5297 | 14.7143 | High |
| SAM2570ff4aae6e | 14.7114 | High |
| SAM2e9ac0b1b250 | 14.70188 | High |
| SAM6d2ae0c39b96 | 14.6911 | High |
| SAM7c67b05aa109 | 14.67789 | High |
| SAM28687037e4ff | 14.6664 | High |
| SAM52500cabdd36 | 14.59867 | High |
| SAM1f66db567eb5 | 14.59373 | High |
| SAMabc151b01ea3 | 14.59108 | High |
| SAMae02629a97f7 | 14.58827 | High |
| SAMf2aae1443f67 | 14.58073 | High |
| SAMe94c30c30616 | 14.57685 | High |
| SAMa913c6139ec8 | 14.56465 | High |
| SAMbcbc7957c264 | 14.55931 | High |
| SAMc6eff056c89a | 14.52742 | High |
| SAMd86389d0d768 | 14.51916 | High |
| SAM9539a4f19ebc | 14.51639 | High |
| SAM6792d6e98068 | 14.50582 | High |
| SAM61baf919bb01 | 14.4963 | High |
| SAM49f9b2e57aa5 | 14.44563 | High |
| SAM31f41dd0d6ca | 14.42265 | High |
| SAM0a7c2091dd56 | 14.40176 | High |
| SAM0257bbbbd388 | 14.37069 | High |
| SAMeb29625f76a5 | 14.36893 | High |
| SAM8f2275c36e8c | 14.35625 | High |
| SAM26104d5adc89 | 14.35617 | High |
| SAM4305ab968b90 | 14.33437 | High |
| SAMe0c49ea0df5d | 14.33048 | High |
| SAM80c6183220e6 | 14.31899 | High |
| SAM76a431ba6ce1 | 14.31857 | High |
| SAM9aa6a095a9d6 | 14.31557 | High |
| SAM8e469834acc1 | 14.30325 | High |
| SAMe56c96c51190 | 14.30164 | High |
| SAM0f956e757453 | 14.30085 | High |
| SAMfed609955db9 | 14.29005 | High |
| SAM0a0f2bac4b20 | 14.26677 | High |
| SAMb0d11db9aa79 | 14.25252 | High |
| SAMdf3e42c8672a | 14.24908 | High |
| SAM5d1dfd5207f5 | 14.22303 | High |
| SAM2c9586161ce6 | 14.20977 | High |
| SAM557dde1b9f3e | 14.1871 | High |
| SAM5e3bae090b8c | 14.18329 | High |
| SAM8e8ef2368dfa | 14.16939 | High |
| SAM563d6233dfa2 | 14.15034 | High |
| SAM23095936e611 | 14.14494 | High |
| SAM5c139c5c1c4f | 14.11651 | High |
| SAM23aa15d4a0b0 | 14.11528 | High |
| SAM6780ed436b55 | 14.11294 | High |
| SAM54e58f1b0230 | 14.10104 | High |
| SAM5234688806a7 | 14.08536 | High |
| SAM1c0ecfb3eb63 | 14.08398 | High |
| SAM18039827e1b9 | 14.08209 | High |
| SAM5cfa1699bdb7 | 14.07397 | High |
| SAM2e7aa8fa0ab3 | 14.04941 | High |
| SAM3ee5dcd894f0 | 14.03724 | High |
| SAM2070b416069c | 14.03538 | High |
| SAM0ce9c983b20f | 14.02045 | High |
| SAM18bc1078bc15 | 14.01148 | High |
| SAMd2492b2a31bb | 14.00541 | High |
| SAMad83c9c53537 | 14.00369 | High |
| SAM19fec8f3b3bd | 13.98807 | High |
| SAM1f83ebd6be9b | 13.98695 | High |
| SAMd215b503f99a | 13.97509 | High |
| SAM87a8e18eb45b | 13.97376 | High |
| SAM0bdb3428bd13 | 13.97139 | High |
| SAM1e9c4d1d39ae | 13.96917 | High |
| SAMe97af0feefdf | 13.94622 | High |
| SAMe7bf6c015192 | 13.94128 | High |
| SAM28e6031ac18b | 13.92572 | High |
| SAMd4c0837b0997 | 13.91728 | High |
| SAM7fb7a13c096b | 13.91711 | High |
| SAM181b638b8248 | 13.87743 | High |
| SAMfd947610629d | 13.87133 | High |
| SAM753d4bb52dbe | 13.86698 | High |
| SAM1fa6bcb7fc48 | 13.86523 | High |
| SAMe3d4266775a9 | 13.85703 | High |
| SAM29da928587ad | 13.85499 | High |
| SAM7893196e0e89 | 13.85373 | High |
| SAMf82bbdc267c8 | 13.85363 | High |
| SAM9d2494119c05 | 13.84527 | High |
| SAMe9ae8beb82fa | 13.83489 | High |
| SAM4caabd64e7fd | 13.83076 | High |
| SAM14938611a2d3 | 13.82702 | High |
| SAM075e037d95bc | 13.8007 | High |
| SAM59f392864f5d | 13.78755 | High |
| SAMae1690469964 | 13.76929 | High |
| SAM3e04eb914f3d | 13.75456 | High |
| SAM727c0e92a2a7 | 13.74917 | High |
| SAM548551ef782c | 13.74341 | High |
| SAM65afda25b920 | 13.73539 | High |
| SAM6157c8f38b72 | 13.72738 | High |
| SAM14df63a65411 | 13.72417 | High |
| SAMc1251c7bfee2 | 13.72239 | High |
| SAMe50d15fde368 | 13.71787 | High |
| SAMc0d625a50eb8 | 13.71121 | High |
| SAMffa5c7cad0e5 | 13.70573 | High |
| SAMb8070b7937e7 | 13.69854 | High |
| SAMf28c01545593 | 13.6906 | High |
| SAMab8052a03398 | 13.67481 | Low |
| SAM6083aac8db99 | 13.67326 | Low |
| SAM8884fe446d20 | 13.67179 | Low |
| SAM6cb230f208a8 | 13.67072 | Low |
| SAMa535fcdf18a0 | 13.65565 | Low |
| SAM2bba8cb35e48 | 13.654 | Low |
| SAMc919aebc7fdd | 13.65379 | Low |
| SAM18a4dabbc557 | 13.6491 | Low |
| SAM1abf01dd4544 | 13.64754 | Low |
| SAMfb7aec7cb0e2 | 13.64488 | Low |
| SAM1c8b086175ca | 13.64293 | Low |
| SAM75f12d1a55fc | 13.63975 | Low |
| SAM85f0a3ac1c45 | 13.63595 | Low |
| SAMeff2ce356ccb | 13.63579 | Low |
| SAMf3a9bce50099 | 13.62695 | Low |
| SAM3894ac3956a5 | 13.62022 | Low |
| SAM8533e5e261d6 | 13.61922 | Low |
| SAM025b45c27e05 | 13.61023 | Low |
| SAMc57eadb2d82b | 13.61019 | Low |
| SAMcf018fee2acd | 13.60826 | Low |
| SAMb963dda93cfd | 13.6026 | Low |
| SAMe7bcab05402e | 13.60232 | Low |
| SAM47fc46c3d6be | 13.59159 | Low |
| SAMae4da274eded | 13.58876 | Low |
| SAM5fe7a81a39dd | 13.57956 | Low |
| SAM9daccafc18db | 13.56098 | Low |
| SAMaaf505c36f93 | 13.55799 | Low |
| SAMf275eb859a39 | 13.54481 | Low |
| SAM49d48750e294 | 13.53618 | Low |
| SAMa424c75831b4 | 13.52265 | Low |
| SAM58e7832f4e7d | 13.52232 | Low |
| SAM2f228939632f | 13.49847 | Low |
| SAMbfdffb97c446 | 13.48944 | Low |
| SAM675a12a09c15 | 13.45659 | Low |
| SAMe9475f77504b | 13.45082 | Low |
| SAMcc4675f394a1 | 13.44989 | Low |
| SAM8b4b8b0f9e73 | 13.4497 | Low |
| SAM18be5b395318 | 13.44302 | Low |
| SAMdab9ca8fb5de | 13.4336 | Low |
| SAM27299aed7681 | 13.42992 | Low |
| SAM572f19794c96 | 13.42815 | Low |
| SAM2624229effe8 | 13.42439 | Low |
| SAMdad5c29dc105 | 13.40183 | Low |
| SAM75142fcab9df | 13.37385 | Low |
| SAM73663ee4a96e | 13.37298 | Low |
| SAM3b15b4c6311d | 13.36909 | Low |
| SAM6dd7ad1d797d | 13.36626 | Low |
| SAM415f36ad349e | 13.35807 | Low |
| SAM59fda9035d1d | 13.35692 | Low |
| SAMbf1a3ae828e6 | 13.34502 | Low |
| SAM553c3c35b847 | 13.29305 | Low |
| SAMba7176afe070 | 13.28897 | Low |
| SAMe1eb5d988760 | 13.28841 | Low |
| SAM6f2a102a99df | 13.28743 | Low |
| SAM9fb814c22bdb | 13.2834 | Low |
| SAM63b2189c36d7 | 13.25057 | Low |
| SAMbda79f955628 | 13.25052 | Low |
| SAM716f54e468f4 | 13.25012 | Low |
| SAM203dcf14f927 | 13.24321 | Low |
| SAM91c47b054ffb | 13.24008 | Low |
| SAM5d989c86255e | 13.2329 | Low |
| SAMd3601288319e | 13.23035 | Low |
| SAMeaa477a5384b | 13.22145 | Low |
| SAM18b9351e265a | 13.21893 | Low |
| SAMeb587a68006b | 13.20843 | Low |
| SAM166a419a4e5a | 13.20323 | Low |
| SAM30cf07d4874f | 13.19212 | Low |
| SAM568ce160abd9 | 13.17782 | Low |
| SAM7aa01fc49a80 | 13.17247 | Low |
| SAMd697ba701077 | 13.16759 | Low |
| SAMa9ca8536d2b1 | 13.15483 | Low |
| SAM63405b04ab2d | 13.15211 | Low |
| SAM961d04c42bd9 | 13.1283 | Low |
| SAM99a46b9eec27 | 13.11916 | Low |
| SAM61b9d4d84c64 | 13.11332 | Low |
| SAMbcb07ba81cee | 13.11273 | Low |
| SAMee3844cc0b9f | 13.08336 | Low |
| SAMba1a34b5a060 | 13.07688 | Low |
| SAM6ff654a20f98 | 13.06283 | Low |
| SAM4b7ea015fd9e | 13.06123 | Low |
| SAM1dda30f1c5be | 13.02841 | Low |
| SAMcb132b0cdd2c | 13.02321 | Low |
| SAMc1b27bc16435 | 13.01107 | Low |
| SAMef0e3d2415fd | 13.00259 | Low |
| SAM36a9225b0222 | 12.99502 | Low |
| SAMb15ac6e4c4ef | 12.98389 | Low |
| SAM032c642382a7 | 12.94945 | Low |
| SAM065890737112 | 12.93093 | Low |
| SAMcc7a42d87e9c | 12.92933 | Low |
| SAM297c0301e861 | 12.92657 | Low |
| SAM7fb6987514a4 | 12.91515 | Low |
| SAMd636e3461955 | 12.90856 | Low |
| SAMd7d57ee3a863 | 12.8812 | Low |
| SAM9a2cf3c06fb3 | 12.87058 | Low |
| SAMa1e62d323e1d | 12.84598 | Low |
| SAMd135d5867fe3 | 12.84123 | Low |
| SAM4b0175e8db6e | 12.83705 | Low |
| SAM0d855cff64e6 | 12.7714 | Low |
| SAM9410b866974a | 12.76101 | Low |
| SAMbe83eae4026e | 12.74958 | Low |
| SAMdee1011782cd | 12.72606 | Low |
| SAMb470eb8f04be | 12.72477 | Low |
| SAM81b71522417a | 12.70337 | Low |
| SAMbc8dc3a7b54e | 12.70249 | Low |
| SAM7d2dfba6cd84 | 12.70079 | Low |
| SAM771445e92421 | 12.69873 | Low |
| SAM560f23d6a3ad | 12.69078 | Low |
| SAMf20b827dca51 | 12.65788 | Low |
| SAM5cc2d9036053 | 12.64275 | Low |
| SAM9306c5c92444 | 12.63951 | Low |
| SAM9725303dce0c | 12.61894 | Low |
| SAMc2a1820d4e6b | 12.61419 | Low |
| SAM5b57e47fdcb3 | 12.60091 | Low |
| SAMc692536a795a | 12.55779 | Low |
| SAMd43f8933066b | 12.51716 | Low |
| SAMdcae54fcd7fa | 12.51381 | Low |
| SAMc0ef41aa6c8b | 12.49805 | Low |
| SAM62fb1388c871 | 12.45098 | Low |
| SAM7b40007f4aa4 | 12.44322 | Low |
| SAM34430ef08e5b | 12.36313 | Low |
| SAMc0da5d48686d | 12.36046 | Low |
| SAM957378bd907f | 12.3421 | Low |
| SAMb419a8fcbfcd | 12.34125 | Low |
| SAM7f0d9cc7f001 | 12.32231 | Low |
| SAMe07c4560772d | 12.31033 | Low |
| SAMe712352fb82a | 12.2927 | Low |
| SAM9cafb905b36a | 12.21394 | Low |
| SAM0571f17f4045 | 12.18495 | Low |
| SAM822b226466a1 | 12.17778 | Low |
| SAM2eb07dedf07f | 12.08821 | Low |
| SAMd35318127278 | 12.00465 | Low |
| SAM07a93a28f801 | 11.97913 | Low |
| SAM3b1066e5801b | 11.96537 | Low |
| SAM3330c03fdf00 | 11.89453 | Low |
| SAM7d7c54623618 | 11.85108 | Low |
| SAM52e3fa3ad574 | 11.83432 | Low |
| SAM1ab1b28d9f2b | 11.81037 | Low |
| SAMd3bd67996035 | 11.77729 | Low |
| SAMdb3f50c9129c | 11.70377 | Low |
| SAM2dc3f04e45e9 | 11.57935 | Low |
| SAM3e8baff50d7a | 11.56433 | Low |
| SAMd027124354ce | 11.52868 | Low |
| SAM97a00e0929fb | 11.37558 | Low |
| SAM8a1b0e02ee42 | 11.26273 | Low |
| SAMaabf4afe4213 | 11.25185 | Low |
| SAMb2e4a082541a | 11.04294 | Low |
| SAMaec7380f9ab0 | 9.444293 | Low |

Table S6: Risk score of each PAAD sample (GSE78220).

| **GSE78220 Samples** | **Risk score** | **Group** |
| --- | --- | --- |
| Pt19.baseline | 1866.644 | High |
| Pt1.baseline | 1620.474 | High |
| Pt14.baseline | 1466.25 | High |
| Pt13.baseline | 1406.081 | High |
| Pt12.baseline | 1388.836 | High |
| Pt7.baseline | 1261.28 | Low |
| Pt28.baseline | 1215.288 | Low |
| Pt31.baseline | 1048.167 | Low |
| Pt23.baseline | 909.8282 | Low |
| Pt10.baseline | 886.1645 | Low |
| Pt9.baseline | 629.8405 | Low |
| Pt2.baseline | 529.5005 | Low |
| Pt27B.baseline | 444.8311 | Low |
| Pt20.baseline | 427.8 | Low |
| Pt15.baseline | 413.2086 | Low |
| Pt27A.baseline | 401.9485 | Low |
| Pt5.baseline | 370.5701 | Low |
| Pt32.baseline | 358.0087 | Low |
| Pt6.baseline | 342.2576 | Low |
| Pt8.baseline | 276.8809 | Low |
| Pt22.baseline | 266.1063 | Low |
| Pt25.baseline | 261.1839 | Low |
| Pt37.baseline | 184.4373 | Low |
| Pt16.OnTx | 172.9183 | Low |
| Pt35.baseline | 172.4032 | Low |
| Pt29.baseline | 168.9624 | Low |
| Pt38.baseline | 129.7002 | Low |
| Pt4.baseline | 124.5543 | Low |
